# Single-cell atlas of the human brain vasculature across development, adulthood and disease

**DOI:** 10.1038/s41586-024-07493-y

**Published:** 2024-07-10

**Authors:** Thomas Wälchli, Moheb Ghobrial, Marc Schwab, Shigeki Takada, Hang Zhong, Samuel Suntharalingham, Sandra Vetiska, Daymé Rodrigues Gonzalez, Ruilin Wu, Hubert Rehrauer, Anuroopa Dinesh, Kai Yu, Edward L. Y. Chen, Jeroen Bisschop, Fiona Farnhammer, Ann Mansur, Joanna Kalucka, Itay Tirosh, Luca Regli, Karl Schaller, Karl Frei, Troy Ketela, Mark Bernstein, Paul Kongkham, Peter Carmeliet, Taufik Valiante, Peter B. Dirks, Mario L. Suva, Gelareh Zadeh, Viviane Tabar, Ralph Schlapbach, Hartland W. Jackson, Katrien De Bock, Jason E. Fish, Philippe P. Monnier, Gary D. Bader, Ivan Radovanovic

**Affiliations:** 1 Group Brain Vasculature and Perivascular Niche, Division of Experimental and Translational Neuroscience, Krembil Brain Institute, Krembil Research Institute, Toronto Western Hospital, University Health Network, University of Toronto, Toronto, Ontario Canada; 2 Division of Neurosurgery, Department of Surgery, University of Toronto, Toronto, Ontario Canada; 3 Group of CNS Angiogenesis and Neurovascular Link, Neuroscience Center Zurich, University of Zurich and University Hospital Zurich, Zurich, Switzerland; 4 Division of Neurosurgery, University Hospital Zurich, Zurich, Switzerland; 5 Laboratory of Exercise and Health, Institute of Exercise and Health, Department of Health Sciences and Technology; Swiss Federal Institute of Technology (ETH Zurich), Zurich, Switzerland; 6 Institute for Regenerative Medicine, University of Zurich, Zurich, Switzerland; 7 Department of Neurosurgery, Kyoto University Graduate School of Medicine, Kyoto, Japan; 8 Division of Experimental and Translational Neuroscience, Krembil Brain Institute, Krembil Research Institute, Toronto Western Hospital, University Health Network, University of Toronto, Toronto, Ontario Canada; 9 Department of Physiology, Faculty of Medicine, University of Toronto, Toronto, Ontario Canada; 10 Functional Genomics Center Zurich, ETH Zurich/University of Zurich, Zurich, Switzerland; 11 Department of Laboratory Medicine and Pathobiology, University of Toronto, Toronto, Ontario Canada; 12 Toronto General Hospital Research Institute, University Health Network, Toronto, Ontario Canada; 13 The Lunenfeld-Tanenbaum Research Institute, Mount Sinai Health System, Toronto, Ontario Canada; 14 Department of Molecular Genetics, University of Toronto, Toronto, Ontario Canada; 15 Department of Biomedicine, Aarhus University, Aarhus, Denmark; 16 Department of Molecular Cell Biology, Weizmann Institute of Science, Rehovot, Israel; 17 Department of Neurosurgery, University of Geneva Medical Center & Faculty of Medicine, University of Geneva, Geneva, Switzerland; 18 The Donnelly Centre, University of Toronto, Toronto, Ontario Canada; 19 Division of Neurosurgery, Sprott Department of Surgery, University of Toronto, Toronto, Ontario, Canada; 20 MacFeeters-Hamilton Centre for Neuro-Oncology Research, University Health Network, Toronto, Ontario Canada; 21 Laboratory of Angiogenesis and Vascular Metabolism, Center for Cancer Biology, VIB & Department of Oncology, KU Leuven, Leuven, Belgium; 22 State Key Laboratory of Ophthalmology, Zhongshan Ophthalmic Center, Sun Yat-sen University, Guangzhou, P. R. China; 23 Laboratory of Angiogenesis and Vascular Heterogeneity, Department of Biomedicine, Aarhus University, Aarhus, Denmark; 24 Krembil Brain Institute, Division of Clinical and Computational Neuroscience, Krembil Research Institute, Toronto Western Hospital, University Health Network, University of Toronto, Toronto, Ontario Canada; 25 Institute of Biomaterials and Biomedical Engineering and Electrical and Computer Engineering, University of Toronto, Toronto, Ontario Canada; 26 Institute of Medical Science Faculty of Medicine, University of Toronto, Toronto, Ontario Canada; 27 Division of Neurosurgery, Arthur and Sonia Labatt Brain Tumor Research Center, Departments of Surgery and Molecular Genetics, Hospital for Sick Children, Toronto, Ontario Canada; 28 Department of Pathology and Center for Cancer Research, Massachusetts General Hospital and Harvard Medical School, Boston, MA USA; 29 Broad Institute of Harvard and MIT, Cambridge, MA USA; 30 Princess Margaret Cancer Centre, University Health Network, Toronto, Ontario Canada; 31 Department of Neurosurgery, Memorial Sloan Kettering Cancer Center, New York, NY USA; 32 Ontario Institute of Cancer Research, Toronto, Ontario Canada; 33 Peter Munk Cardiac Centre, University Health Network, Toronto, Ontario Canada; 34 Krembil Research Institute, Vision Division, Krembil Discovery Tower, Toronto, Ontario Canada; 35 Department of Ophthalmology and Vision Sciences, Faculty of Medicine, University of Toronto, Toronto, Ontario Canada; 36 Department of Computer Science, University of Toronto, Toronto, Ontario Canada

**Keywords:** Neuro-vascular interactions, Computational biology and bioinformatics, Cancer, Developmental biology, Genetics

## Abstract

A broad range of brain pathologies critically relies on the vasculature, and cerebrovascular disease is a leading cause of death worldwide. However, the cellular and molecular architecture of the human brain vasculature remains incompletely understood^1^. Here we performed single-cell RNA sequencing analysis of 606,380 freshly isolated endothelial cells, perivascular cells and other tissue-derived cells from 117 samples, from 68 human fetuses and adult patients to construct a molecular atlas of the developing fetal, adult control and diseased human brain vasculature. We identify extensive molecular heterogeneity of the vasculature of healthy fetal and adult human brains and across five vascular-dependent central nervous system (CNS) pathologies, including brain tumours and brain vascular malformations. We identify alteration of arteriovenous differentiation and reactivated fetal as well as conserved dysregulated genes and pathways in the diseased vasculature. Pathological endothelial cells display a loss of CNS-specific properties and reveal an upregulation of MHC class II molecules, indicating atypical features of CNS endothelial cells. Cell–cell interaction analyses predict substantial endothelial-to-perivascular cell ligand–receptor cross-talk, including immune-related and angiogenic pathways, thereby revealing a central role for the endothelium within brain neurovascular unit signalling networks. Our single-cell brain atlas provides insights into the molecular architecture and heterogeneity of the developing, adult/control and diseased human brain vasculature and serves as a powerful reference for future studies.

## Abstract (web summary)

Endothelial cells from vascular-dependent central nervous system (CNS) diseases reveal reactivated fetal pathways, display common hallmarks of disease — including a partial loss of arteriovenous specification and CNS-specific properties as well as an upregulation of MHC class II receptors — and play a key role in the human brain neurovascular unit across development, adulthood and disease.

## Main

The brain vasculature is important for both the proper functioning of the normal brain as well as for a variety of vascular-dependent CNS pathologies such as brain tumours, brain vascular malformations, stroke and neurodegenerative diseases^1–9^. A better understanding of the underlying cellular and molecular mechanisms and architecture of the vasculature during brain development, in the healthy adult brain, as well as in vascular-dependent brain diseases, has broad implications for both the biological understanding as well as the therapeutic targeting of the pathological brain vasculature^10–15^. Vascular growth and network formation, involving endothelial cells (ECs) and other cells of the neurovascular unit (NVU), are highly dynamic during brain development, almost quiescent in the healthy adult brain and reactivated in a variety of angiogenesis-dependent brain pathologies, including brain tumours and brain vascular malformations^3,7,16–21^, thereby activating ECs and perivascular cells (PVCs) of the NVU and other tissue-derived cells (hereafter collectively referred to as PVCs). However, it is unclear which molecular signalling cascades are reactivated and how they regulate brain tumour and brain vascular malformation vascularization and growth.

**Fig. 1:**
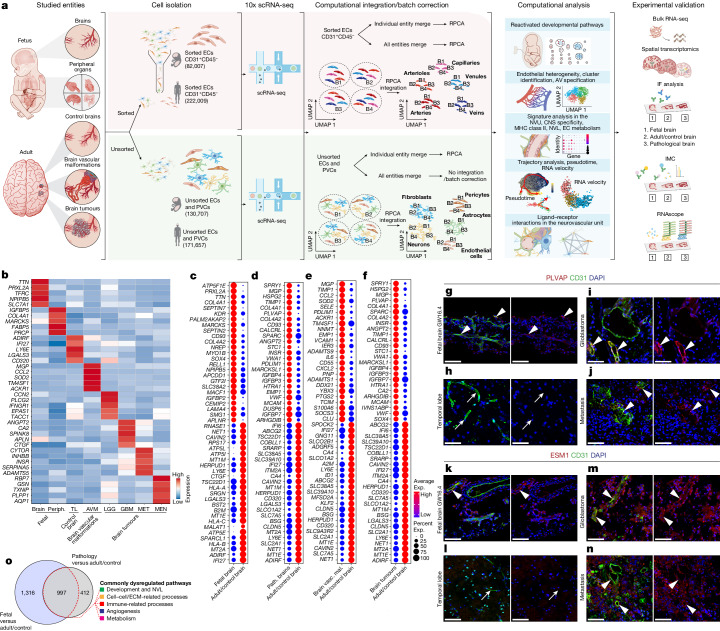
Construction of a molecular sc-atlas of the human brain vasculature and reactivation of fetal programmes in pathological brain vascular ECs. **a**, Schematic of the experimental workflow including scRNA-seq, computational analysis summary and validation experiments. **b**, Expression heat map of the top ranking marker genes in the indicated tissues. For the colour scale, red shows high expression, white shows intermediate expression and blue shows low expression. **c**–**f**, Dotplot heatmap of the fetal versus adult/control brain endothelium (**c**); pathological (path.) versus adult/control brain endothelium (**d**); brain vascular malformations (brain vasc. mal.) versus adult/control brain endothelium (**e**); and brain tumours versus adult/control brain endothelium (**f**) signatures based on differential gene expression analyses. **g**–**n**, IF imaging of tissue sections from the indicated entities, stained for *PLVAP* (red; **g**–**j**), *ESM1* (red; **k**–**n**) and *CD31* (green). Nuclei are stained with DAPI (blue). The arrowheads indicate expression of PLVAP or ESM1 in blood-vessel ECs in the different tissues, and the arrows indicate the absence of expression in blood-vessel ECs in the different tissues. For **g**–**n**, scale bars, 50 μm. **o**, The overlap between the 2,313 significant pathways enriched in fetal brain ECs as compared to adult/control brain ECs and the 1,409 significant gene sets enriched in pathological brain ECs as compared to adult/control brain ECs. ECM, extracellular matrix; NVL, neurovascular link; periph., periphery; RPCA, reciprocal principal component analysis; UMAP, uniform manifold approximation and projection.

**Extended Data Fig. 1:**
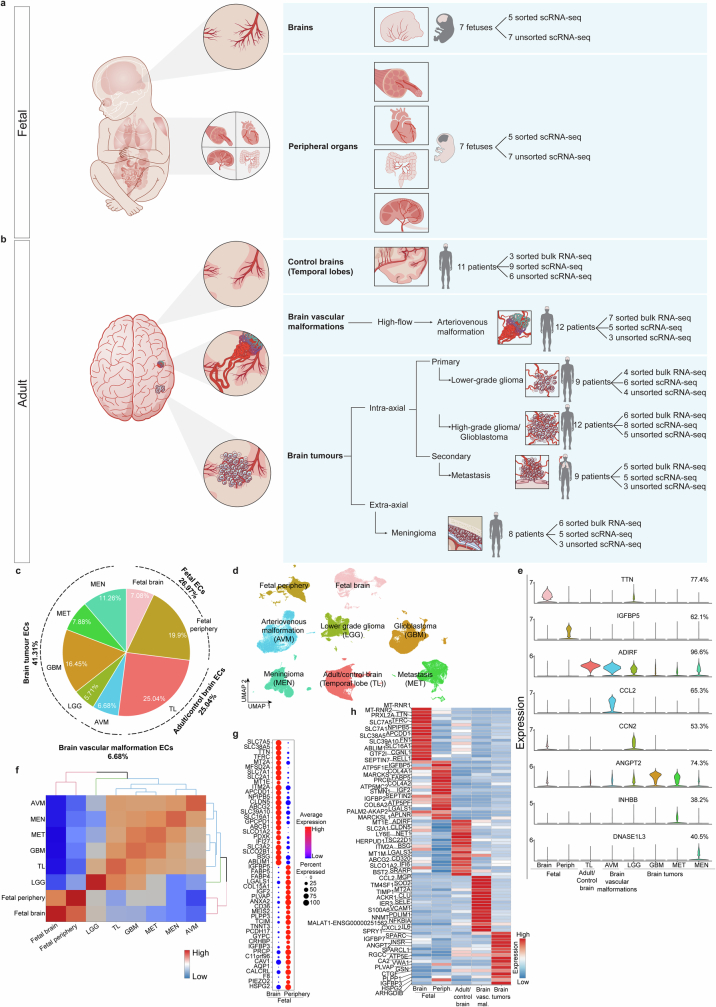
Construction of a molecular single-cell atlas of the human brain vasculature across development, adulthood and disease and of the fetal peripheral vasculature - studied entities and inter-tissue heterogeneity. Figure shows studied entities and inter-tissue heterogeneity of sorted vascular endothelial cells. **a**,**b**, Scheme of the different tissue types present in the study with respective sample/patient numbers of fetal (**a**) and adult (**b**) origins. **c**, Piechart showing relative abundance and percentage of ECs from each tissue collected. **d**, Composite UMAP plots of sorted and in silico-quality checked (ECs, coloured by tissue of origin. **e**, Violin plots of the expression of the top marker genes of each tissue type, percentage of cells expressing the marker gene is indicated on the right. **f**, Endothelial cells transcriptome correlation heatmap and hierarchical clustering of all tissues analysed. **g**, Dotplot heatmap of the fetal brain vs fetal periphery endothelial signature. **h**, Expression heatmap of top ranking marker genes in the indicated tissues. Colour scale: red, high expression; white, intermediate expression; blue, low expression.

The CNS vasculature has unique features such as the blood–brain barrier (BBB) and the NVU^22–24^. During development, various CNS-specific and general signalling pathways drive CNS angiogenesis^3,7,23,25–27^. The brain vasculature also displays an arteriovenous (AV) endothelial hierarchy similar to peripheral vascular beds^28–30^. Developmentally regulated signalling axes in ECs are thought to contribute to the establishment of CNS-specific properties as well as AV specification of the endothelium in the healthy adult brain and to their alteration in disease^13,14^. Over the past years, single-cell transcriptome atlases of human peripheral organs^31–33^, the human brain vasculature^34–37^, and the mouse brain and peripheral vasculature^28,38^ were established. Nevertheless, a landscape of the human brain vasculature at the single-cell level across fetal development, adulthood and various vascular-dependent diseases with a focus on the brain vascular endothelium is lacking. Here we created a comprehensive molecular atlas of the human brain vasculature using single-cell RNA sequencing (scRNA-seq) analysis in the developing, adult/control and diseased human brain (Fig. 1a, Extended Data Fig. 1 and Supplementary Methods). We identified extensive heterogeneity among ECs as well as common hallmarks across a spectrum of multiple brain pathologies, including commonly regulated angiogenic signalling pathways that significantly overlap with the fetal signalling axes, altered AV specification and CNS specificity, upregulation of MHC class II signalling and strong EC–EC/EC–PVC communication networks.

## Constructing a sc-atlas of the human brain vasculature

We constructed a human brain vasculature single-cell atlas (sc-atlas) using samples from fetal as well as adult control (undiseased atlas) and diseased brains, including adult brain vascular malformations and brain tumours (diseased atlas). We acquired freshly isolated cells (both fluorescence-activated cell sorting (FACS)-sorted ECs and unsorted ECs and PVCs; Supplementary Tables 1–4) from 8 individual fetuses^39–41^ and from 61 adult brain samples (from 61 individual patients), covering adult temporal lobe (TL) controls and adult vascular-dependent pathologies, including brain vascular malformations, namely, brain AV malformation (AVM)^36,42^, and brain tumours, notably, lower-grade glioma (LGG)^22,43^, glioblastoma (GBM)^22,44,45^, lung cancer brain metastasis (MET)^22,46^ and meningioma (MEN)^47^ (Fig. 1a, Extended Data Fig. 1a,b and Supplementary Tables 1–4). Brain tissue samples were dissociated into single-cell suspensions, which were either FACS-sorted for ECs (CD31^+^CD45^−^) or processed as unsorted samples to examine all cells of the NVU (Fig. 1a). Single-cell transcriptomes were collected using the 10x Genomics Chromium system^48^ and analysed. CD31^+^CD45^−^ ECs showed consistent expression of classical endothelial markers, such as *CD31*, *VWF* and *CLDN5*, while not expressing PVC markers (Supplementary Fig. 1a–o), thereby confirming the purity of EC isolations. In summary, 606,380 single cells, including 304,016 sorted ECs and 302,364 unsorted ECs and PVCs, passed the quality-control criteria (Fig. 1a and Supplementary Tables 3–5). The number of sorted ECs analysed here substantially exceeds the number of ECs analysed using scRNA-seq^36,37^ or single-nucleus RNA-seq^34,35^ in previous studies, and we directly compared single-cell transcriptomics of sorted ECs from the vasculature of the fetal and adult brain and of various brain pathologies. Notably, we report higher numbers of sorted ECs and of unsorted ECs and PVCs in the different brain entities compared with previous studies^34–37,49^, enabling us to assess EC heterogeneity across development, adulthood and disease at a high resolution.

To address the role of the endothelium within the brain NVU across different entities, fetal, adult/control and pathological unsorted EC and PVC transcriptomes from 31 patients were analysed (Fig. 1a and Supplementary Fig. 2a–f). We identified 18 major brain cell types, including all known vascular, perivascular and other tissue-derived cell types in the human brain. The detected cell type distributions within the NVU differed between the fetal, adult control and pathological brain samples (Supplementary Figs. 2e–g and 3a–n and Supplementary Tables 10–16). Key signatures and differentially expressed genes (DEGs) were validated using bulk RNA-seq, RNAscope, spatial transcriptomics, immunofluorescence (IF) and imaging mass cytometry (IMC) (Fig. 1a).

**Extended Data Fig. 2:**
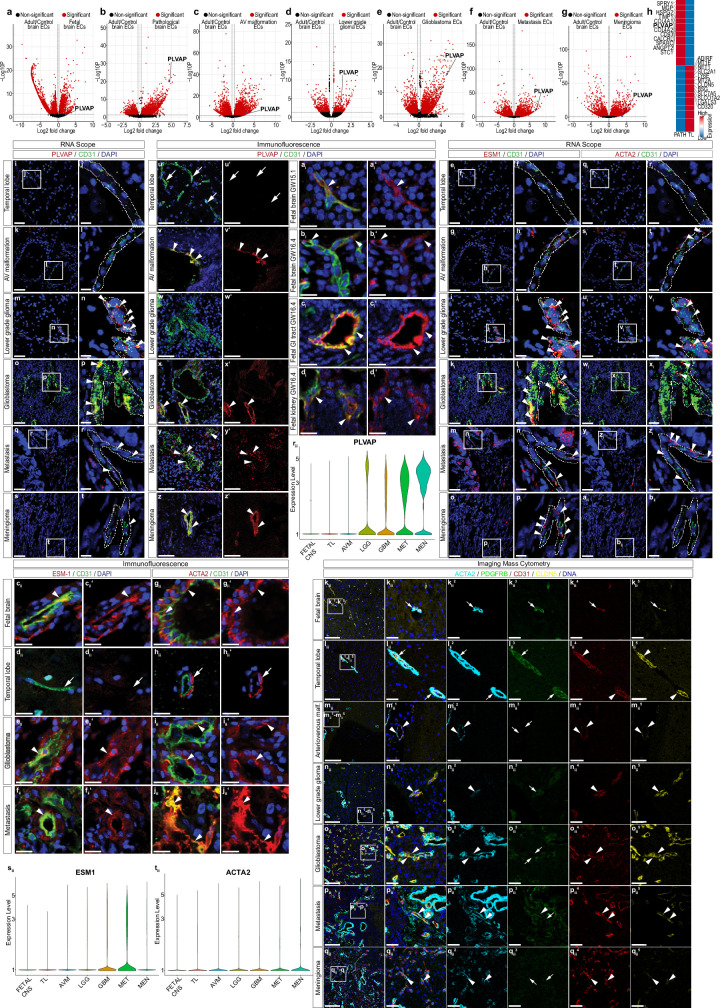
Validation of angiogenic and EndoMT markers in vascular ECs of the fetal, adult and pathological brain vasculature using RNAscope, IF and IMC. **a**-**g**, Volcano plots showing the differential expression analysis comparing endothelial cells from adult/control brains (left) and the indicated entity (right) (Wald test, Benjamini Hochberg correction; ***P***-value < 0.05 and log2FC ≥ 0.25 coloured significant in red, PLVAP dot is coloured blue). Number of individuals analysed is as follows: Fetal brain=5, Adult/control brain (TL) = 9, AV malformations=5, AVM = 5, LGG = 6, GBM = 8, MET = 5, MEN = 5. **h**, Expression heatmap of the top 25 differentially expressed genes in adult/control ECs (TL) vs. pathological brain ECs (PATH). **i-d**_**i**_**‘**, Immunofluorescence (IF) and RNAscope imaging of tissue sections from the indicated entities, stained for PLVAP (red), (RNAscope: **i-t**; IF: **u**-**d**_**i**_**‘**) and CD31 (green). Nuclei are stained with DAPI (blue). Arrowheads indicate ECs expressing PLVAP and dotted lines (RNAscope) delineate vascular structures in the different tissues. Scale bars: 50 μm in overviews (RNAscope); 50 μm in (IF) and 12.5 μm in zooms (RNAscope). **a**_**i**_**-d**_**i**_**‘**, IF of fetal brain tissue sections from the indicated gestational ages, stained for PLVAP (red) and CD31 (green). Nuclei are stained with DAPI (blue). Arrowheads indicate ECs expressing PLVAP. Arrows indicate absence of expression in ECs. Scale bars: 20 μm. **e**_**i**_**-b**_**ii**_, RNAscope imaging of tissue sections from the indicated entities, stained for ESM1 (red; **e**_**i**_**-p**_**i**_), ACTA2 (red; **q**_**i**_**-b**_**ii**_) and CD31 (green). Nuclei are stained with DAPI (blue). Boxed area is magnified on the right; arrowheads indicate ECs expressing ESM1 or ACTA2 and dotted lines delineate vascular structures in the different tissues. Scale bars: 50 μm in overviews and 12.5 μm in zooms. **c**_**ii**_**-j**_**ii**_**‘**, IF of tissue sections from the indicated entities, stained for ESM1 (red; **c**_**ii**_**-f**_**ii**_**‘**), ACTA2 (red; **g**_**ii**_-**j**_**ii**_**‘**) and CD31 (green). Nuclei are stained with DAPI (blue). Boxed area is magnified on the right; arrowheads indicate ECs expressing ESM1 or ACTA2. Arrows indicate absence of expression in ECs. Scale bars: 80 μm in overviews and 20 μm in zooms. **k**_**ii**_-**q‘**^**4**^, Mass cytometry (IMC) imaging of the indicated fetal, adult/control and pathological brain tissue samples visualizing metal-conjugated ACTA2, PDGFRB, CD31 and CLDN5 primary antibodies out of all 39 stained panel (Supplementary Table 24). Overlay of pseudocolor images as well as individual channels of the different markers (white, overlap; cyan, ACTA2; green, PDGFRB; red, CD31; yellow, CLDN5; blue, DNA intercalator), Scale bars: 250 μm in overviews, 50 μm in zooms. Arrowheads identify blood vessels ECs expressing the indicated markers in the different tissues. Arrows identify blood vessel ECs not expressing the indicated markers in the different tissues. **r**_**ii**_**-t**_**ii**_, Violin plots showing the expression of PLVAP (**r**_**ii**_), ESM1 (**s**_**ii**_) and ACTA2 (**t**_**ii**_) in the indicated fetal brain (CNS), adult/control and pathological brain tissues.

We next compared ECs in the sorted samples across entities and found that ECs from different entities exhibited prominent transcriptomic heterogeneity (Extended Data Fig. 1c,f) as well as distinct gene expression signatures (Fig. 1b–f, Extended Data Fig. 1d,e,g,h and Supplementary Fig. 5). We defined major EC signatures, including a human fetal CNS (and peripheral) signature characterizing CNS and periphery-specific markers of the fetal vasculature (Extended Data Fig. 1g and Supplementary Table 6), a human fetal/developmental CNS/brain signature revealing properties of the developing and mature human brain vasculature, and a pathological signature of the diseased brain vasculature including a brain vascular malformation and a brain tumour signature (Fig. 1d–f, Supplementary Fig. 5a–c and Supplementary Table 25). The fetal and pathological brain EC signatures revealed differential expression of the well-known angiogenic markers *PLVAP* and *ESM1*^36,38,50–56^, which we confirmed in the fetal and diseased brain entities using IF analysis^36,38,52–56^ (Fig. 1g–n and Extended Data Fig. 2).

Although all of the entities revealed distinct EC markers, some EC markers were conserved across two or more entities (such as *ADIRF*, *EGR1*, *PLPP1* and *ANGPT2*) (Fig. 1b, Extended Data Fig. 1e,h and Supplementary Tables 8 and 9).

## Reactivation of fetal programmes in pathological brain ECs

We further assessed the differences in ECs across developmental stages and in pathological conditions (Supplementary Tables 7 and 25). DEGs between the fetal and adult/control stage and between the adult/control and pathological brain showed developmental and pathology-specific gene and pathway enrichments (Supplementary Fig. 5d–f), providing insights into functional specialization of the human brain vasculature across development, homeostasis and disease (Supplementary Fig. 5). Using various approaches, including statistical regression, we found no evidence that age and sex^57–59^ are confounders of our findings (Supplementary Tables 13 and 14). The top differentially regulated pathways in both fetal versus adult/control as well as in pathological versus adult/control brain EC signatures belonged to five main groups, including development and neurovascular link^3^, cell–cell/extracellular-matrix-related processes, immune-related processes, angiogenesis and metabolism (Supplementary Fig. 5d–f). Notably, of the 1,409 differentially regulated pathways in pathology versus adult/control brain ECs, more than half (997) also showed differential regulation in fetal versus adult/control brain ECs (Fig. 1o and Supplementary Fig. 5f), highlighting the importance of developmental pathways in vascular-dependent brain pathologies. Bulk RNA-seq analysis confirmed the scRNA-seq findings, including the dysregulated pathways across pathologies (Supplementary Fig. 6 and Supplementary Table 26). Together, these data indicate that signalling axes driving vascular growth during fetal brain development are silenced in the adult control brain and reactivated in the vasculature of brain tumours and brain vascular malformations and that common dysregulated pathways are observed in the pathological brain vascular endothelium across diseases.

## Inter-tissue heterogeneity and AV zonation of brain ECs

**Fig. 2:**
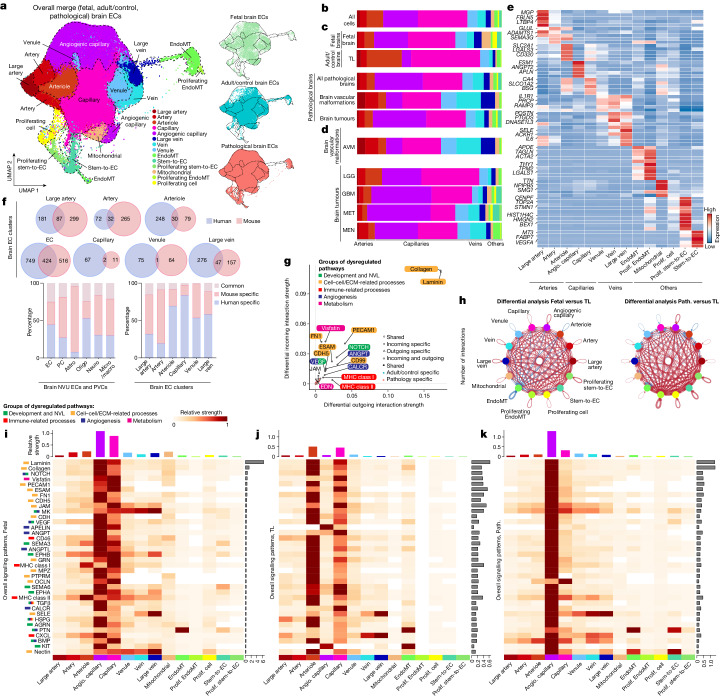
Inter-tissue heterogeneity and AV zonation of brain vascular ECs. **a**, UMAP plot of the 243,521 integrated/batch corrected fetal, adult/control and pathological brain ECs across 5 (fetal), 9 (adult/control) and 29 (pathological) individuals (Supplementary Table 3), colour coded by EC AV specification, and UMAP plots split by tissue of origin: fetal brain (5 individuals), adult/control brain (9 individuals) and brain pathologies (29 individuals). **b**–**d**, The relative abundance of EC subtypes (AV specification cluster) from the indicated tissue of origin. **b**–**d** are coloured according to the colour code in **a** (Supplementary Table 10). The number of individuals analysed was as follows: *n* = 43 (all entities), *n* = 5 (fetal brain), *n* = 9 (adult/control brain (TL)), *n* = 29 (all pathological brains), *n* = 5 (brain vascular malformations), *n* = 24 (brain tumours), *n* = 5 (AVM), *n* = 6 (LGG), *n* = 8 (GBM), *n* = 5 (MET) and *n* = 5 (MEN). **e**, The top ranking marker gene expression levels in different EC subtypes. For the colour scale, red shows high expression, white shows intermediate expression and blue shows low expression. Angio., angiogenic; prolif., proliferating. **f**, The overlap between human and mouse AV specification markers (of large artery, artery, arteriole, capillary, venule and large vein) and endothelial (EC) markers (top). Bottom, the percentage of common, human-specific and mouse-specific cell/AV specification markers. Astro, astrocytes; micro/macro, microglia/macrophages; neuro, neurons; PC, pericytes. **g**, Scatter plot showing the differential incoming and outgoing interaction strength of pathways in angiogenic capillaries, identifying signalling changes in those cells in pathological as compared to the control conditions. **h**, The number of statistically significant ligand–receptor interactions between EC subtypes in fetal versus adult/control brains (left) and pathological (path.) versus adult/control brains (right). The circle plots show a differential analysis of the intercellular signalling interactions; red indicates upregulation and blue indicates downregulation. **i**,**j**,**k**, The overall signalling patterns of different EC subtypes in fetal (**i**), adult/control (**j**) and pathological (**k**) brains. Grey bars indicate signalling strength.

To further address EC heterogeneity across different brain entities at the single-cell level, we pooled, integrated and batch-corrected (using RPCA)^60–63^, clustered and visualized all fetal (21,512), adult/control (76,125) and pathological (145,884) sorted brain EC transcriptomes from 43 patients (Fig. 2a and Supplementary Figs. 7a–e and 9). Brain vascular ECs are organized along the human brain AV axis, referred to as AV zonation^28,64–69^. Endothelial clusters were biologically annotated using DEGs across entities, and we identified 44 EC subclusters (Fig. 2e, Supplementary Fig. 7 and Supplementary Table 18) that were arranged according to AV zonation, which we grouped into 14 major EC clusters for further downstream analysis (Fig. 2a and Supplementary Fig. 7a,h).

We characterized AV zonation markers^28,70^ with arterial (subclustered into large artery, artery, arteriole) and venous (subclustered into large vein, vein, venule) clusters located at the opposite ends of the uniform manifold approximation and projection (UMAP), separated by major capillary clusters (subdivided into capillary and angiogenic capillary) in the fetal, adult and pathological brains (Fig. 2a,e, Supplementary Figs. 7 and 10 and Supplementary Tables 18 and 19), providing unprecedented transcriptional resolution by AV zonation^34–36^.

While we confirmed differential expression of known marker genes of AV specification^28,38^, we also identified AV-zonation markers that have not to our knowledge been identified previously in the human brain: *LTBP4* (large arteries); *ADAMTS1* (arteries); *VSIR*, *AIF1L*, *CD320* and others (arterioles); *SLC38A5*, *BSG*, *SLC16A1* and *SLCO1A2* (capillaries); *JAM2*, *PRCP*, *PRSS23* and *RAMP3* (venules); *PTGDS*, *POSTN* and *DNASE1L3* (veins); *CCL2* (large veins); and *PLVAP*, *ESM1* and *CA2* (angiogenic capillaries) (Fig. 2e, Supplementary Fig. 7i and Supplementary Tables 18 and 19). *PLVAP* and *ESM1* were among the top markers of the angiogenic capillary cluster and we confirmed *PLVAP* and *ESM1* expression in diseased brain entities and in the fetal brain, indicating its role in developmental and pathological vascular growth^50,51^. Indeed, *PLVAP* and *ESM1* exhibited RNA and protein expression in human fetal brain and human brain vascular malformation/tumour ECs on the basis of RNAscope and IF analysis (Extended Data Fig. 2).

**Extended Data Fig. 3:**
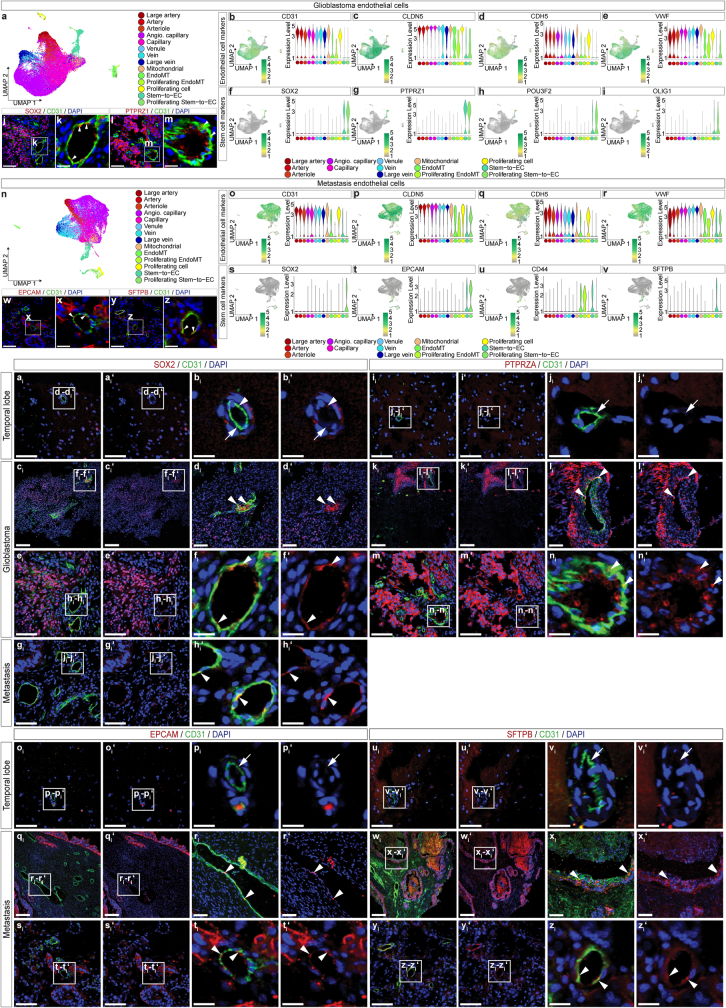
Validation of stem-to-endothelial transdifferentiation in vascular ECs of glioblastoma and of lung cancer brain metastasis using IF. **a**,**n**, UMAP plot of glioblastoma (49,999 ECs from 8 individuals) (GBM) and lung cancer brain metastasis (23,962 ECs from 5 individuals) (MET) endothelial cells, coloured by AV specification. **b**-**i**, **o**-**v**, Violin plots showing the expression of the indicated endothelial- and stem cell specific markers in the different EC subtypes (AV clusters). **j**-**m**, **a**_**i**_**-z**_**i**_**’**, Immunofluorescence staining of *SOX2* (**j**,**k**, **a**_**i**_**-h**_**i**_**‘**), *PTPRZ1* (**l**,**m**, **i**_**i**_**-n**_**i**_**‘**), *EPCAM* (**w**,**x**, **o**_**i**_**-t**_**i**_**‘**), *SFTPB* (**y**,**z**, **u**_**i**_**-z**_**i**_**‘**) (red) and *CD31 *(green) in the indicated tissue samples. Arrowheads identify blood vessels ECs expressing the indicated markers in the different tissues. Arrows identify blood vessel ECs not expressing the indicated markers in the different tissues. Boxed area is magnified on the right. Scale bars: 80 µm in overviews except for **c**_**i**_**-c**_**i**_**‘**,**k**_**i**_**-k**_**i**_**‘**, **q**_**i**_**-q**_**i**_**‘**, **w**_**i**_**-w**_**i**_**‘** (200 µm), 20 µm in zooms except for **d**_**i**_**-d**_**i**_**‘**, **l**_**i**_**-l**_**i**_**‘**, **r**_**i**_**-r**_**i**_**‘**, **x**_**i**_**-x**_**i**_**‘** (50 µm).

We also assigned EC clusters outside AV zonation, notably (proliferating) stem-to-endothelial cell transdifferentiating (stem-to-EC) clusters and (proliferating) endothelial-to-mesenchymal transition (EndoMT) clusters (Fig. 2e and Supplementary Fig. 7a,i), for which we identified specific molecular markers. We found proliferating ECs (such as *TOP2A* and *MKI67*) in the fetal (3.54%), adult (0.4%) and pathological brains (1.37%) (Fig. 2e, Supplementary Fig. 7i and Supplementary Table 19). We identified EndoMT clusters expressing both mesenchymal (such as *APOE*, *ACTA2* and *TAGLN*) and endothelial markers^71^ (Fig. 2e, Supplementary Fig. 7i and Extended Data Fig. 2q_i_–b_ii_,g_ii_–j_ii_′). Notably, we observed two subsets of EndoMT ECs (proliferating EndoMT and EndoMT) that were increased in certain pathologies (MEN > LGG > GBM). Proliferating EndoMT ECs expressed both EndoMT and proliferation markers (for example, *ACTA2* and *MKI67*) (Fig. 2e, Supplementary Fig. 7i and Supplementary Tables 8 and 9). Using RNAscope, IF and IMC, we found ACTA2^+^CD31^+^CLDN5^+^ co-expressing but PDGFRβ^−^ (suggesting no pericyte identity) ECs across pathologies (Extended Data Fig. 2q_i_–b_ii_,g_ii_–j_ii_′,k_ii_–q_ii_^5^), indicating the presence of EndoMT ECs in the diseased vasculature. In GBMs and METs, we observed stem-to-EC clusters that expressed classical EC markers (such as *CD31*, *CLDN5*, *CDH5* and *VWF*) to a lower level compared with other EC clusters, as well as some markers of (tumour) stem cells (Fig. 2e, Extended Data Fig. 3 and Supplementary Fig. 7i), suggesting ECs undergoing stem-to-EC transdifferentiation. In GBMs, we identified a stem-to-EC cluster expressing the GBM stemness markers *SOX2*, *PTPRZ1*, *POUR3F2* and *OLIG1*^72,73^, and EC markers^74,75^ (Supplementary Fig. 7i and Extended Data Fig. 3a–i,a_i_–n_i_′). In METs, we noted a previously undescribed stem-to-EC population that co-expressed EC markers and stem cell markers of lung cancers (for example, *SOX2*, *EPCAM*, *CD44* and *SFTPB*)^76^ (Extended Data Fig. 3n–v,g_i_–z_i_′ and Supplementary Fig. 7i). In GBMs and METs, we identified groups of stem-to-ECs that co-expressed stemness (for example, *SOX2*, *PTPRZ1*, *EPCAM1* and *SFTPB*) and proliferation markers (for example, *MKI67*, *BEX1*, *HMGB2* and *UBE2C*) (Fig. 2e and Extended Data Fig. 3). To validate the stem-to-EC clusters in GBM and MET, we used double immunostaining for EC and stemness markers. In GBM, we found SOX2^+^CD31^+^ and PTPRZ1^+^CD31^+^ co-expressing ECs, whereas, in MET, we observed EPCAM^+^CD31^+^ and SFTBP^+^CD31^+^ co-expressing ECs (Extended Data Fig. 3). The confirmation of tumour stemness marker enrichment in a subset of tumour ECs suggests the presence of stem-to-ECs in GBM and MET vasculature.

We next addressed the distributions of EC clusters between the fetal, adult/control and pathological brains. Capillaries accounted for around 60.5% of ECs, arterial ECs accounted for 18.2% and venous ECs accounted for 16.2%, in agreement with previous studies^3,17^. We further uncovered previously unrecognized EC heterogeneity across a wide range of human brain tissues (Fig. 2b–d, Supplementary Fig. 10 and Supplementary Tables 10–16). Angiogenic capillary proportions were significantly higher in the fetal brain and in brain tumours (GBM > MET > MEN > LGG), illustrating their angiogenic capacity^3,13,22,77^, whereas brain vascular malformations (AVM) revealed significantly elevated proportions of venous clusters, indicating their venous character^78,79^ (Fig. 2c,d, Supplementary Fig. 10 and Supplementary Table 12). We next evaluated whether AV-zonation markers were conserved across species^34–36,49,80^ (Supplementary Fig. 4). Although the overall structure of AV zonation was conserved between human and mouse, the number of conserved AV-zonation genes in the different AV compartments was low. Accordingly, we found the highest proportion of human-specific AV-zonation markers in small > large-calibre and venous > arterial vessel ECs (Fig. 2f, Supplementary Fig. 4z_xxix_,z_xxx_ and Supplementary Table 17), and we validated these human-specific markers referring to the Human Protein Atlas (HPA)^35,81–83^ (Supplementary Fig. 8).

In the brain NVU, mapping of our dataset to the freshly isolated mouse dataset^70^ revealed high transcriptomic similarity between species for ECs and PVCs^84^ (Supplementary Fig. 4z_xvii–xvix_). We further observed that neurons and astrocytes showed the greatest transcriptional divergence (Fig. 2f and Supplementary Table 17), while ECs and oligodendrocytes displayed the highest percentage of human-specific markers, in agreement with previous studies^34–36^. These species-specific differences along AV-zonation suggest fundamental disparities in brain vascular properties, indicating the necessity to directly study sorted/enriched ECs and PVCs of the human brain vasculature at the single-cell level.

As EC clusters reside in close proximity to each other along the AV tree, we next inferred cell–cell communication pathways^85,86^. Differential analysis revealed increased cellular cross-talk among EC clusters in pathological and fetal ECs, highlighting a key role for angiogenic capillaries. Angiogenic capillaries displayed upregulation of several signalling pathways, including the five above-mentioned groups in both the diseased and fetal (versus adult/control) brain (Fig. 2g–k and Supplementary Fig. 11), highlighting this cluster as a major signalling mediator within brain EC networks.

## Alteration of AV specification in pathological brain ECs

**Fig. 3:**
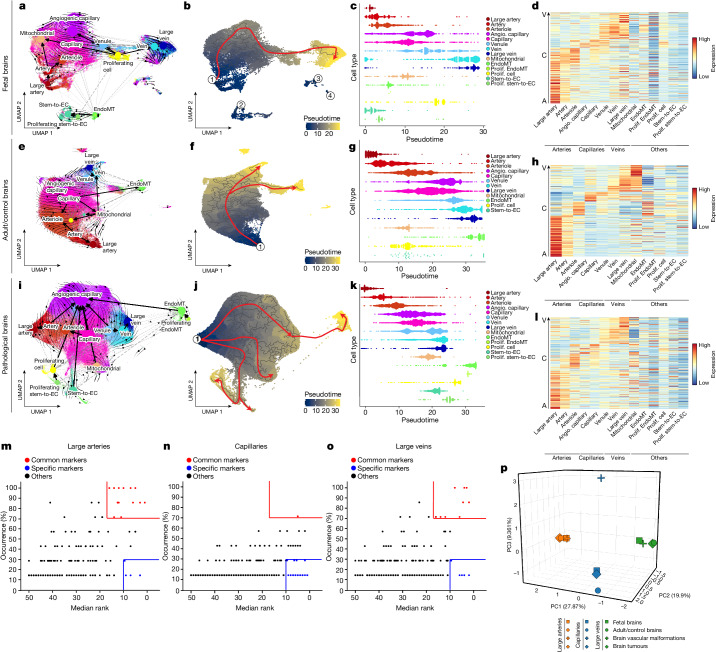
Alteration of AV specification in pathological brain vascular ECs. **a**,**e**,**i**, UMAP plots of human brain ECs isolated from fetal brains (21,512 ECs from 5 individuals; **a**), adult/control brains (76,125 ECs from 9 individuals; **e**) and pathological brains (145,884 ECs from 29 individuals; **i**), coloured by AV specification. RNA velocity streamlines and partition-based graph abstraction (PAGA) vectors extended by velocity-inferred directionality are superimposed onto the UMAPs. **b**,**f**,**j**, UMAP plots of human brain ECs isolated from fetal brains (**b**), adult/control brains (**f**) and pathological brains (**j**), coloured by pseudotime. The red line, which was drawn manually, indicates the major trajectory flow. **c**,**g**,**k**, Pseudotime order of ECs, colour coded according to AV specification from fetal brains (**c**), control adult/control brains (**g**) and pathological brains (**k**). **d**,**h**,**l**, Heat map of adult/control brain EC AV specification signature gene expression in human brain ECs isolated from fetal brains (**d**), adult/control brains (**h**) and pathological brains (**l**). A, arterial; C, capillary; V, venous. **m**,**n**,**o**, Common and tissue-specific markers in ECs from large arteries (**m**), capillaries (**n**) and large veins (**o**) in different tissue types (fetal brain, adult/control brain, brain vascular malformations and brain tumours). The red boxes highlight conserved markers between ECs from different tissues; the blue boxes highlight tissue-specific markers. Dots are coloured as defined in the legend. **p**, Three-dimensional principal component analysis visualization of pairwise Jaccard similarity coefficients between the indicated ECs from the different tissues.

**Extended Data Fig. 4:**
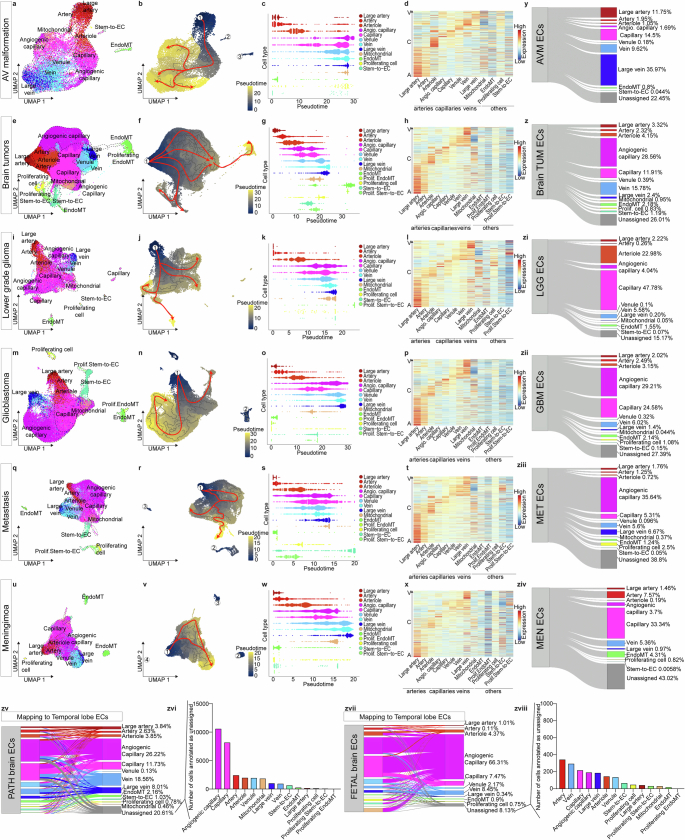
Alteration of AV-specification in pathological brain vascular ECs. UMAP plots of human brain ECs isolated from the brain arteriovenous malformations (20,305 ECs from 5 individuals) (**a**,**b**), all brain tumours (125,579 ECs from 24 individuals) (**e**,**f**), lower-grade glioma (17,373 ECs from 6 individuals) (**i**,**j**) glioblastoma (49,999 ECs from 8 individuals) (**m**,**n**), brain metastasis (23,962 ECs from 5 individuals) (**q**,**r**), and meningioma (34,245 ECs from 5 individuals) (**u**,**v**) coloured by AV specification (**a**,**e**,**i**,**m**,**q**,**u**) and by pseudotime (**b**,**f**,**j**,**n**,**r**,**v**), the red line drawn manually indicates the major trajectory flow. **c**,**g**,**k**,**o**,**s**,**w**, Pseudotime order of ECs colour-coded according to AV specification from all theindicated entities. **d**,**h**,**l**,**p**,**t**,**x**, Heatmap of adult/control brain ECs AV specification signature gene expression in human brain ECs isolated from the indicated entities. **y-ziv**, Sankey plot showing the predicted annotation of the ECs of the indicated entities as mapped to adult/control brain (TL) ECs. Unassigned cells are indicated in grey. **zv**, Sankey plot of pathological brain ECs, showing the predicted annotation (right nodes) – as mapped to adult/control brain (TL) ECs – of the AV clusters assigned based on top cluster marker analysis (left nodes). Unassigned cells are indicated in grey. **zvii**, Sankey plot of fetal brain ECs, showing the predicted annotation (right nodes) – as mapped to adult/control brain (TL) ECs – of the AV clusters assigned based on top cluster marker analysis (left nodes). Unassigned cells are indicated in grey. **zvi**,**zviii**, Barplots showing the composition of unassigned cells – based on mapping to adult/control brain (TL) ECs – in pathological brain ECs (**zv**) and fetal brain ECs (**zvii**).

Failure of proper AV specification in brain vascular malformations and formation of tortuous arteries and veins in brain tumours has been reported^77,87^, but AV specification in brain pathologies and fetal (brain) development remains poorly understood. We ordered ECs along a one-dimensional transcriptional gradient using Monocle^88^ and TSCAN^89^ to examine the AV axis in the different entities. Whereas arterial and venous markers peaked at opposite ends, capillary markers showed peaks in the mid-section throughout all entities (Fig. 3b,f,j, Extended Data Fig. 4b,f,j,n,r,v and Supplementary Fig. 13III), indicating that in silico pseudospace and pseudotime recapitulate in vivo anatomical topography of EC clusters in the human brain vasculature^28,38^. We observed AV zonation throughout the fetal, control and pathological brains, but observed a partial alteration of EC ordering along the AV axis in disease (Fig. 3b,c,f,g,j,k and Extended Data Fig. 4).

We defined an AV signature comprising genes revealing significant expression gradients of ECs along the AV axis (Fig. 3d,h,l and Extended Data Fig. 4d,h,l,p,t,x). The seamless zonation continuum was recapitulated in all entities but again showed alteration in pathologies. While AV markers revealed a clear distinction between AV compartments in the fetal and adult/control brain, showing specific markers of large arteries (for example, *VEGFC*, *FBLN5*), arterioles (such as *LGALS3*, *AIF1L*), capillaries (for example, *SLC35A5*, *MFSD2A*), angiogenic capillaries (such as *ESM1*, *ANGPT2*), venules (for example, *JAM2* and *PRCP*) and large veins (such as *SELE* and *SELP*), some zonation markers showed a less-specific presence in pathologies (Figs. 2 and 3d,h,l and Extended Data Fig. 4d,h,l,p,t,x).

Whereas almost all fetal and pathological ECs were quite similar to temporal-lobe EC clusters^60^, small-calibre vessel ECs were more different compared with their healthy temporal lobe counterparts (Extended Data Fig. 4z_v_–z_viii_), probably pertaining to a higher vulnerability of small-calibre vessel ECs to alterations in the local tissue microenvironment^38^.

**Extended Data Fig. 5:**
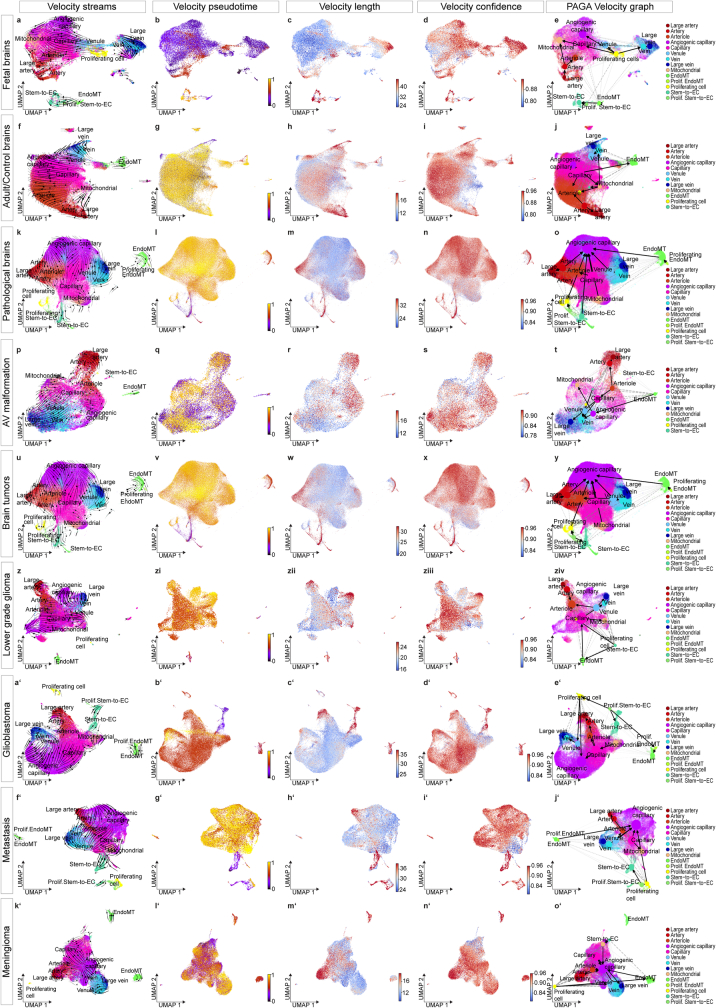
Trajectory analysis of fetal, adult and pathological human brain vascular ECs via RNA velocity along the AV-zonation/specification axis. **a**,**f**,**k**,**p**,**u**,**z**,**a‘**,**f‘**,**k‘**, UMAP plots of human brain ECs isolated from the indicated entities (fetal brains (21,512 ECs from 5 individuals) (**a**), adult/control brains (76,125 ECs from 9 individuals) (**f**), pathological brains (145,884 ECs from 29 individuals) (**k**), brain arteriovenous malformations (20,305 ECs from 5 individuals) (**p**), all brain tumours (125,579 ECs from 24 individuals) (**u**), lower-grade glioma (17,373 ECs from 6 individuals) (**z**) glioblastoma (49,999 ECs from 8 individuals) (**a‘**), brain metastasis (23,962 ECs from 5 individuals) (**f‘**), and meningioma (34,245 ECs from 5 individuals) (**k‘**)) coloured by AV specification, onto the UMAPs are superimposed RNA velocity streamlines extended by velocity-inferred directionality. **b**,**g**,**l**,**q**,**v**,**zi**,**b‘**,**g‘**,**l‘**, UMAP plots of the indicated entities, coloured by RNA velocity pseudotime. **c**,**h**,**m**,**r**,**w**,**zii**,**c‘**,**h‘**,**m‘**, UMAP plots of the indicated entities, coloured by RNA velocity length. **d,i,n,s,x,ziii,d‘,i‘,n‘**, UMAP plots of the indicated entities, coloured by RNA velocity confidence. **e**,**j**,**o**,**t**,**y**,**ziv**,**e‘**,**j‘**,**o‘**, UMAP plots of the indicated entities, coloured by AV specification, onto the UMAPs are superimposed RNA velocity PAGA vectors extended by velocity-inferred directionality.

To further address lineage relationships in AV specification, we referred to RNA velocity^90,91^ and diffusion map^92^, revealing vectors from multiple EC clusters towards the angiogenic capillary cluster in angiogenic entities (tumours > fetal brain > vascular malformations), whereas, in vascular malformations, we observed vectors from various EC clusters towards venous clusters (Fig. 3a,e,i, Extended Data Fig. 5 and Supplementary Fig. 13). These results indicate that RNA velocity can suggest timeline relationships among human brain vascular ECs^23,29^.

We next addressed whether EC markers of AV clusters were conserved between vascular beds or expressed in a more tissue-specific manner^38^. While we identified multiple conserved markers for large arteries and large veins, capillaries were more tissue/entity specific, indicating a more pronounced transcriptional heterogeneity of the capillary bed across the different brain tissues^38^. Accordingly, capillaries showed more tissue-specific markers than large-calibre vessels (Fig. 3m–p), indicating a higher susceptibility of capillary ECs to the local tissue microenvironment.

## Alteration of CNS specificity in pathological brain ECs

**Extended Data Fig. 6:**
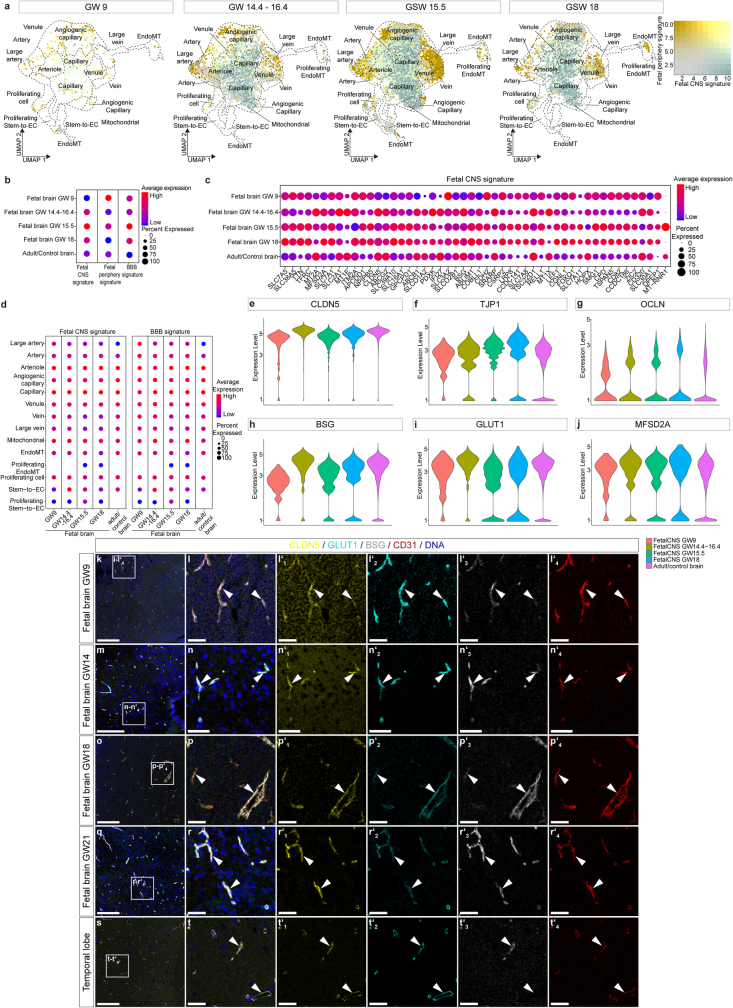
Establishment of CNS, peripheral and BBB signatures in vascular ECs from the developing fetal to the adult brain vasculature. **a**, UMAP plots of the ECs from fetal brains in ascending fetal age ranging from gestational week 9 (444 ECs), gestational week 14.4 and 16.4 (3762 ECs), gestational week 15.5 (18,314 ECs) to gestational week 18 (3601 ECs); plots are colour-coded for fetal CNS signature (green) and fetal peripheral signature (yellow). **b**, Dotplot heatmap of fetal CNS, fetal peripheral, and BBB signatures’ expression in fetal brains of indicated fetal age and adult/control brains (temporal lobes). **c**, Dotplot heatmap of fetal CNS signature genes’ expression in fetal brains of indicated fetal age and adult/control brains (temporal lobes). **d**, Dotplot heatmaps of fetal CNS and BBB signatures expression at the level of AV specification for the indicated entities. Colour scale: red, high expression; blue, low expression, whereas the dot size represents the percentage expression within the indicated entity. **e**-**j**, Violin plots showing the expression of selected CNS specific and BBB marker genes in fetal brains of indicated fetal age and adult/control brains (TL). Markers shown were further validated by IF and/or IMC. **k-t’**_**4**_ Mass cytometry (IMC) imaging of fetal brains of fetal age GW9 (**k-l’**_**4**_), GW14 (**m-n’**_**4**_), GW18 (**o-p’**_**4**_), GW21 (**q-r’**_**4**_) and adult/control brains (temporal lobe) (**s-t’**_**4**_) tissue samples using metal-conjugated antibodies. Representative pseudocolor images of combined and individual channels of the different markers (white, overlap; yellow, CLDN5; cyan, GLUT1; grey, BSG; red, CD31; blue, DNA intercalator), Scale bars: 250 μm in overviews, 50 μm in zooms. Shown are 5 antibodies stained for (Supplementary Table 24). Arrowheads identify blood vessels ECs expressing the indicated markers in the different tissues. Arrows identify blood vessel ECs not expressing the indicated markers in the different tissues.

We next examined CNS-specific properties distinguishing brain ECs from ECs outside the CNS^3,5^. Bulk RNA-seq analysis in mice revealed a BBB-enriched transcriptome^93^, but how the human brain EC CNS properties differ at the single-cell level and whether they are heterogeneous across developmental stages and in disease remains largely unclear. The development of the human fetal BBB (occurring between gestational week 8 and 18)^39,40,94^ is controversial^41,95^. We therefore studied human fetal BBB development at a high resolution. Referring to a human/mouse BBB signature (Supplementary Table 20 and Extended Data Fig. 6a–j), we observed an increased BBB signature expression with increased gestational age (Extended Data Fig. 6b–j), in agreement with a previous study^96^. Along the AV compartments, the BBB signature revealed a higher expression in small- versus large-calibre vessels across developmental stages (Extended Data Fig. 6d), in agreement with previous findings^97^ and probably pertaining to the susceptibility of capillaries to the local microenvironment^38^.

**Fig. 4:**
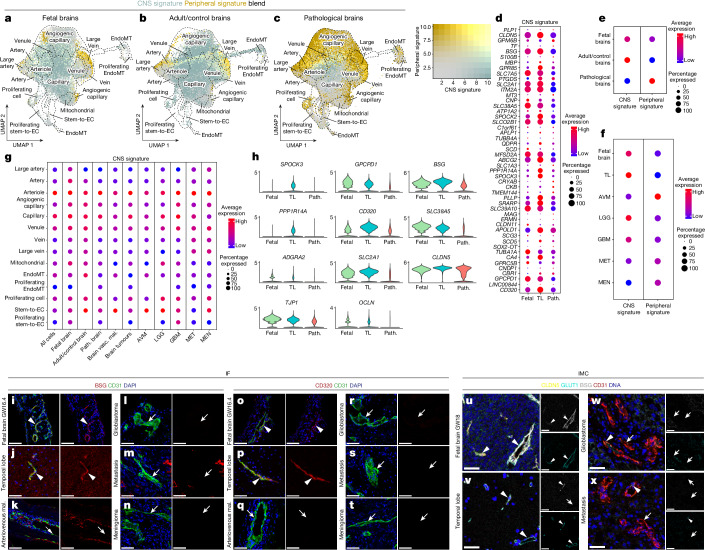
Alteration of CNS specificity in pathological brain vascular ECs. **a**–**c**, UMAP plots of the ECs from fetal brains (21,512 ECs from 5 individuals; **a**), adult/control brains (76,125 ECs from 9 individuals; **b**) and pathological brains (145,884 ECs from 29 individuals; **c**). Plots are colour coded for CNS signature (green) and peripheral signature (yellow). **d**, CNS signature genes expression in fetal brain, adult/control brains (temporal lobes) and pathological brain ECs. **e**,**f**, CNS and peripheral signature expression in fetal brain, adult/control brain and pathological brain ECs (**e**) and in each individual entity (**f**). **g**, The CNS signature at the level of AV specification for the indicated entities. For the colour scale, red shows high expression and blue shows low expression. The dot size represents the percentage expression within the indicated entity. **h**, The expression of representative CNS-specific and BBB marker genes for fetal brain versus adult/control brain versus pathological brain ECs. **i**–**t**, IF images for the protein expression of *BSG* (**i**–**n**) and *CD320* (**o**–**t**) in fetal brain (**i** and **o**), adult/control brain (TL; **j** and **p**), brain AVMs (**k** and **q**), GBM/high-grade glioma (**l** and **r**), metastasis (**m** and **s**) and meningioma (**n** and **t**). For **i**–**t**, scale bars, 80 μm (fetal brain) and 50 μm (adult control and pathological brains). **u**–**x**, IMC imaging of fetal brain (**u**), adult/control brain (TL; **v**), GBM/high-grade glioma (**w**) and metastasis (**x**) tissue samples visualizing five metal-conjugated antibodies (Supplementary Table 24). Representative pseudocolour images of *CLDN5*, *GLUT1*, *BSG* and *CD31* combined, and individual *GLUT1* and *BSG* channels are shown; white, overlap; yellow, *CLDN5*; cyan, *GLUT1*; grey, *BSG*; red, *CD31*; blue, DNA intercalator. For **u**–**x**, scale bars, 50 μm. The arrowheads indicate blood-vessel ECs expressing the indicated markers in the different tissues. The arrows indicate blood-vessel ECs not expressing the indicated markers in the different tissue.

Next, to address molecular differences of CNS and peripheral ECs at the single-cell level, we defined a human adult and fetal CNS signature (Fig. 4a–f, Supplementary Fig. 14l–q and Supplementary Table 20). These include known BBB (*MFSD2A*^98^ and *CLDN5*^99^) and capillary markers (*CA4*^28,100^ and *SPOCK2*^38^), as well as novel genes enriched in the CNS vasculature such as *SPOCK3*, *BSG* and *CD320* (Supplementary Fig. 14b,e and Supplementary Table 20). The adult and fetal CNS signatures showed elevated expression with increasing gestational age and in small- versus large-calibre vessels across developmental stages, reminiscent of the BBB signature expression pattern (Fig. 4g and Extended Data Fig. 6d).

CNS properties were observed in the fetal and adult brains, whereas alteration of the CNS signature and concomitant acquisition of the peripheral signature was seen in pathologies (Fig. 4a–f and Supplementary Figs. 14 and 15). Comparing the CNS signature between pathological and adult/control brain ECs, we found downregulation of *SLC2A1*, which is dysregulated in neurodegenerative conditions^69^; the lipid transporter *MFSD2A*, which is expressed in brain ECs and restricts caveolae-mediated transcytosis at the BBB^98,101,102^; and the BBB marker *CLDN5* (Fig. 4d–f and Supplementary Table 20), therefore suggesting BBB alteration in pathologies^22^. The CNS signature was highest in the temporal lobe, followed by intra-axial primary brain tumours and fetal brain (LGG > fetal brain > GBM), brain vascular malformations, intra-axial secondary brain tumour MET and extra-axial brain tumour MEN, whereas the peripheral signature followed an inversed pattern (Fig. 4e,f and Supplementary Fig. 14p,q).

We next addressed CNS-specific properties along the AV axis. In the fetal and adult brain, the CNS signature was mainly expressed by small-calibre vessels, while the peripheral signature was predominantly present in large arteries and large veins. We observed a similar pattern in pathological brains with, however, a notable decrease in cells expressing the CNS signature (most pronounced for angiogenic capillaries > capillaries), paralleled by an increase in cells expressing the peripheral signature predominantly for angiogenic capillaries and large-calibre vessels (Fig. 4g and Supplementary Fig. 14h).

We next examined how the CNS, BBB and peripheral signatures changed along the AV axis in each pathological entity. The CNS and BBB signatures were downregulated in every pathology with a similar pattern to that described above and reaching the highest baseline values of CNS specificity at the capillary and arteriole levels, with the capillaries being the cluster mostly affected by pathologies^38^ (Fig. 4g and Supplementary Fig. 14h), probably pertaining to the influence of the local microenvironment for small-calibre vessels. The peripheral signature was upregulated in disease, peaking for AVM > MEN > MET > GBM and lower expression for LGG, predominantly affecting large-calibre vessels and angiogenic capillaries (Fig. 4h and Supplementary Figs. 14 and 15). These data indicate that CNS ECs acquire CNS-specific properties during fetal-to-adult transition and take on a peripheral EC signature in disease conditions.

The CNS and BBB signatures are tightly linked to a functional BBB in vivo^103^ and BBB dysfunction affects the CNS properties of ECs^93^. We therefore investigated the human and mouse BBB dysfunction modules, with the latter being upregulated in CNS ECs after various disease triggers in the mouse brain, shifting CNS ECs into peripheral EC-like states^93^. We found that the human and mouse BBB dysfunction modules were upregulated in human brain tumours and brain vascular malformations as well as in the fetal brain (Supplementary Fig. 16a–n), probably due to pathways related to BBB dysfunction^93^. Both the human and mouse BBB dysfunction modules were highest in AVM > GBM > MET > MEN (Supplementary Fig. 16a–n). The BBB dysfunction modules expression along the AV axis revealed enrichment in large-calibre vessels and angiogenic capillaries, mimicking the peripheral signature expression, again indicating that pathological CNS ECs take on a peripheral endothelial gene expression^93^ (Supplementary Fig. 16h–n). Comparison to BBB dysfunction modules in human Alzheimer’s disease^35^, Huntington’s disease^34^ and AVMs^36^ revealed some overlap with the human and mouse BBB dysfunction modules^93^ (Supplementary Fig. 16o–s and Supplementary Table 21), indicating common and distinct features among brain diseases.

We confirmed decreased expression of the CNS-signature genes *SPOCK3*, *BSG*, *CD320*, *PPP1R14A* and *SLC38A5* in all brain tumours and brain vascular malformations (Fig. 4h–x and Extended Data Figs. 6k–t′_4_, 9 and 10) using IF and IMC, thereby highlighting the alteration of CNS properties in the diseased human cerebrovasculature.

## Upregulation of MHC class II in pathological brain ECs

We identified EC populations expressing the MHC class II genes *CD74*, *HLA-DRB5*, *HLA-DMA*, *HLA-DPA1* and *HLA-DRA* in pathological CNS tissues. This antigen-presenting signature, indicating possible immune functions of human brain ECs, prompted us to investigate the heterogeneity of MHC class II transcripts between tissues at the single-cell level.

**Fig. 5:**
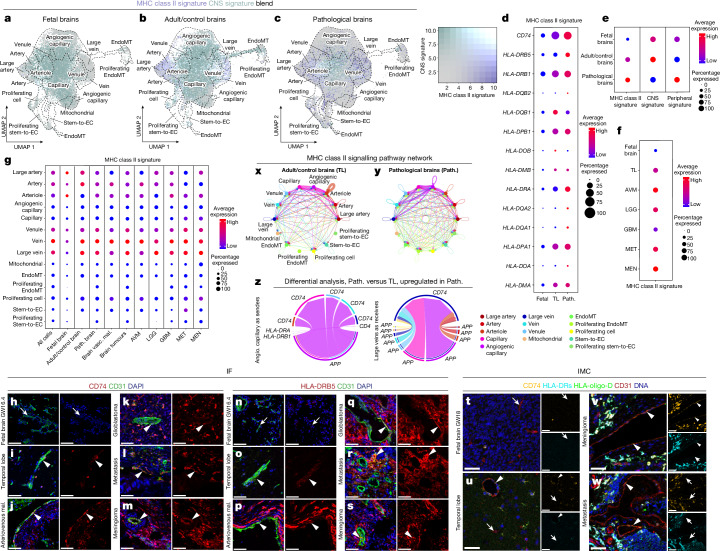
Upregulation of MHC class II receptors in pathological brain vascular ECs. **a**–**c**, UMAP plots of ECs from fetal (21,512 ECs from 5 individuals; **a**), adult/control (76,125 ECs from 9 individuals; **b**) and pathological brains (145,884 ECs from 29 individuals;**c**). Plots are colour coded for MHC class II (violet) and CNS (green) signatures. **d**, MHC class II signature gene expression in fetal, adult/control (temporal lobes) and pathological brain ECs. **e**,**f**, MHC class II, CNS and peripheral signature expression in fetal, adult/control and pathological brain ECs (**e**), and MHC class II signature expression in each individual entity (**f**). **g**, The MHC class II signature at the level of AV specification for the indicated entities. For the colour scale, red shows high expression and blue shows low expression. The dot size represents the percentage expression within the indicated entity. **h**–**s**, IF images for the protein expression of *CD74* and *HLA-DRB5* in the fetal brain (**h** and **n**) and the adult/control brain (TL; **i** and **o**), in brain AVMs (**j** and **p**), in GBM/high-grade glioma (**k** and **q**), in metastasis (**l** and **r**) and in meningioma (**m** and **s**). For **h**–**s**, scale bars, 80 μm (fetal brain) and 50 μm (adult control and pathological brains). **t**–**w**, IMC imaging of fetal brain (**t**), adult/control brain (**u**), meningioma (**v**) and metastasis (**w**) tissue samples visualizing metal-conjugated *CD74*, pan-HLA-DR, oligo-HLA-D and *CD31* primary antibodies. An overlay of pseudocolour images as well as individual channels for *CD74* and pan-HLA-DR are shown; white, overlap; orange, *CD74*; cyan, pan-HLA-DR; green, oligo-HLA-D; red, *CD31*; blue, DNA intercalator. For **t**–**w**, scale bars, 50 μm. **x**,**y**, The strength of MHC class II signalling interactions between the different EC subtypes of the adult/control brain (**x**) and pathological brain (**y**) ECs at the AV specification level. **z**, Differential analysis of MHC class II ligand–receptor pairs. Chord/circos plots showing upregulated MHC class II signalling in angiogenic capillaries as the source and all other cell clusters as targets (left), and large veins as receivers (right). The edge thickness represents its weight. The edge colour indicates the sender cell type. The arrowheads indicate ECs expressing the indicated markers. The arrows indicate ECs not expressing the indicated markers.

**Extended Data Fig. 9:**
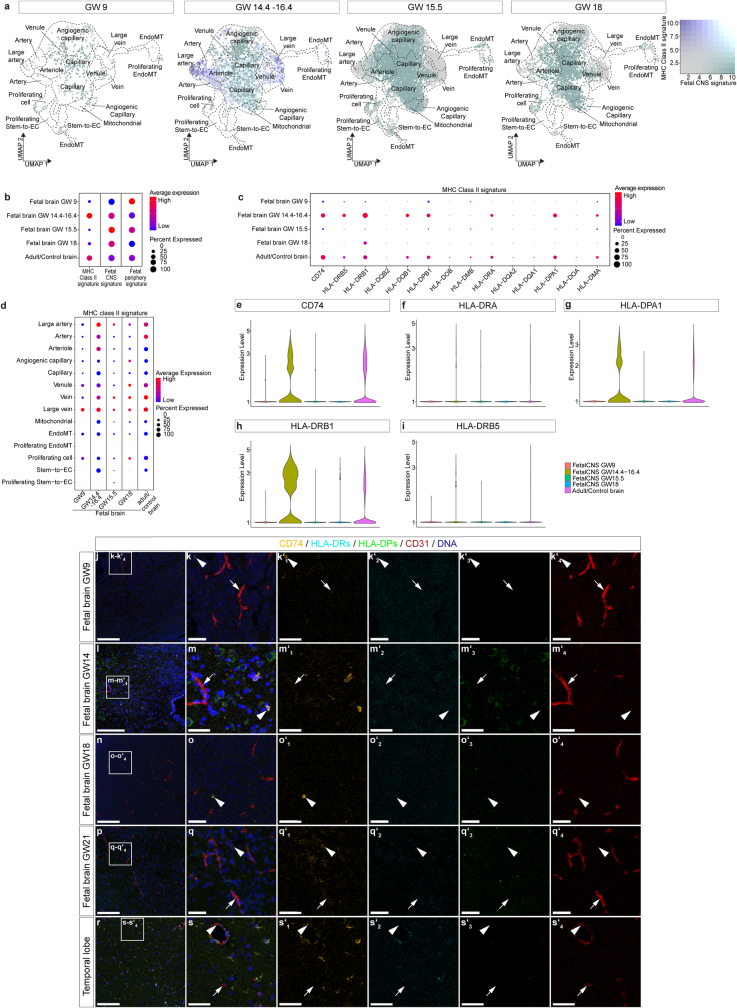
Establishment of MHC class II and CNS signatures in vascular ECs from the developing fetal to the adult brain vasculature. **a**, UMAP plots of the ECs from fetal brains in ascending fetal age ranging from gestational week 9 (444 ECs), gestational week 14.4 and 16.4 (3762 ECs), gestational week 15.5 (18,314 ECs) to gestational week 18 (3601 ECs); plots are colour-coded for fetal CNS signature (green) and MHC class II signature (blue). **b**, Dotplot heatmap of MHC class II, fetal CNS and peripheral signatures expression in fetal brains of indicated fetal age and adult/control brains (temporal lobes). **c**, Dotplot heatmap of MHC class II signature genes’ expression in fetal brains of indicated fetal age and adult/control brains (temporal lobes). **d**, Dotplot heatmaps of MHC class II signature expression at the level of AV specification for the indicated entities. Colour scale: red, high expression; blue, low expression, whereas the dot size represents the percentage expression within the indicated entity. **e**-**i**, Violin plots showing the expression of MHC class II marker genes in fetal brains of indicated fetal age and adult/control brains. Markers shown were further validated by IF and/or IMC. **j-s’**_**4**_ Mass cytometry (IMC) imaging of fetal brains of fetal age GW9 (**j-k’**_**4**_), GW14 (**l-m’**_**4**_), GW18 (**n-o’**_**4**_), GW21 (**p-q’**_**4**_) and adult/control brain (**r-s’**_**4**_) tissue samples using metal-conjugated primary and secondary antibodies. Representative pseudocolour images of combined and individual channels of the different markers (white, overlap; orange, CD74; cyan, pan-HLA-DR; green, oligo-HLA-D; red, CD31; blue, DNA intercalator), Scale bars: 250 μm in overviews, 50 μm in zooms. Shown are 5 antibodies stained for (Supplementary Table 24). Arrowheads identify blood vessels ECs expressing the indicated markers in the different tissues. Arrows identify blood vessel ECs not expressing the indicated markers in the different tissues.

scRNA-seq identified endothelial MHC class II expression in peripheral human and mouse tissues^31,100^, but assessment of MHC class II expression in human brain vascular beds at the single-cell level is lacking. To assess MHC class II gene expression across brain development and disease, we defined a human MHC class II signature including MHC class II receptors (Fig. 5d and Supplementary Table 22). The MHC class II signature was upregulated in pathologies, and low in the fetal brain (Fig. 5a–f and Extended Data Fig. 9), in agreement with a previous study^31^. We found that the MHC class II signature was highest in AVM > MEN > MET, followed by LGG > GBM, the temporal lobe and the fetal brain, grossly following the peripheral signature expression gradient (Fig. 5f). We examined MHC class II signature expression patterns according to AV zonation. Whereas, in the fetal brain, only very few ECs (large arteries and arterioles) expressed the MHC class II signature, mainly large-calibre vessel (large arteries and large veins) ECs expressed a signature of genes involved in MHC class II-mediated antigen presentation in the adult brain (Fig. 5a–c,g).

The MHC class II signature was upregulated in all pathologies according to the pattern described above^38^ (Fig. 5a–c,g–z). We observed a partial overlap of the MHC class II and peripheral signatures and of the BBB dysfunction module with a common predominance in large-calibre vessels, but a more widespread/stronger expression of the peripheral signature and BBB dysfunction module in angiogenic capillaries consistent with previous findings^69^. These data suggest that pathological CNS ECs upregulate MHC class II receptors in brain tumours and brain vascular malformations.

**Extended Data Fig. 10:**
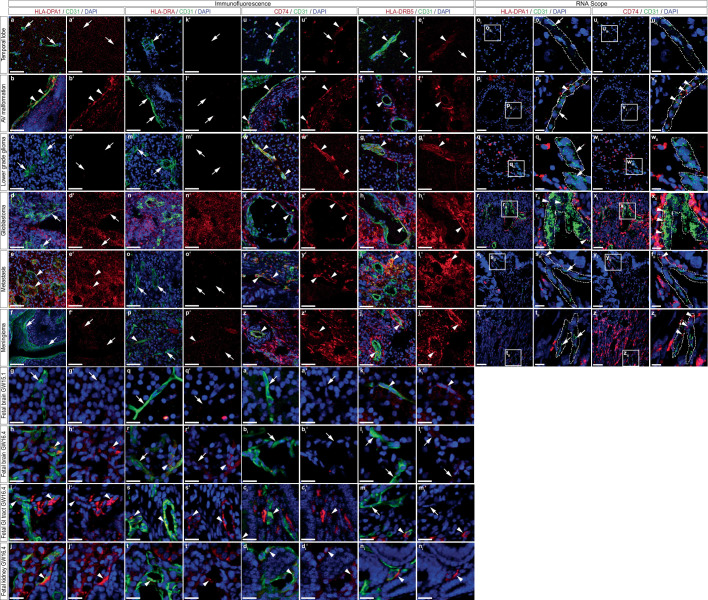
Validation of MHC class II signature markers in vascular ECs of the developing fetal brain and periphery vasculature, and of the adult and pathological brain vasculature using RNAscope and IF. Immunofluorescence (IF) and RNAscope imaging of tissue sections from the indicated entities, stained for HLA-DPA1 (red; **a-j‘**, IF; **o**_**i**_**-t**_**ii**_, RNAscope), HLA-DRA (red; **k-t‘**, IF), CD74 (red; **u-d**_**i**_**‘**, IF; **u**_**i**_**-z**_**ii**_, RNAscope), HLA-DRB5 (red; **e**_**i**_**-n**_**i**_**‘**, IF) and CD31 (green). Nuclei are stained with DAPI (blue). Arrowheads identify blood vessel ECs expressing the indicated markers, arrows identify blood vessel ECs not expressing the indicated markers in the different tissues and dotted lines (RNAscope) indicate vascular structures in the different tissues. Scale bars: 50 μm in (IF), 50 μm in overviews (RNAscope) and 12.5 μm in zooms (RNAscope).

**Extended Data Fig. 11:**
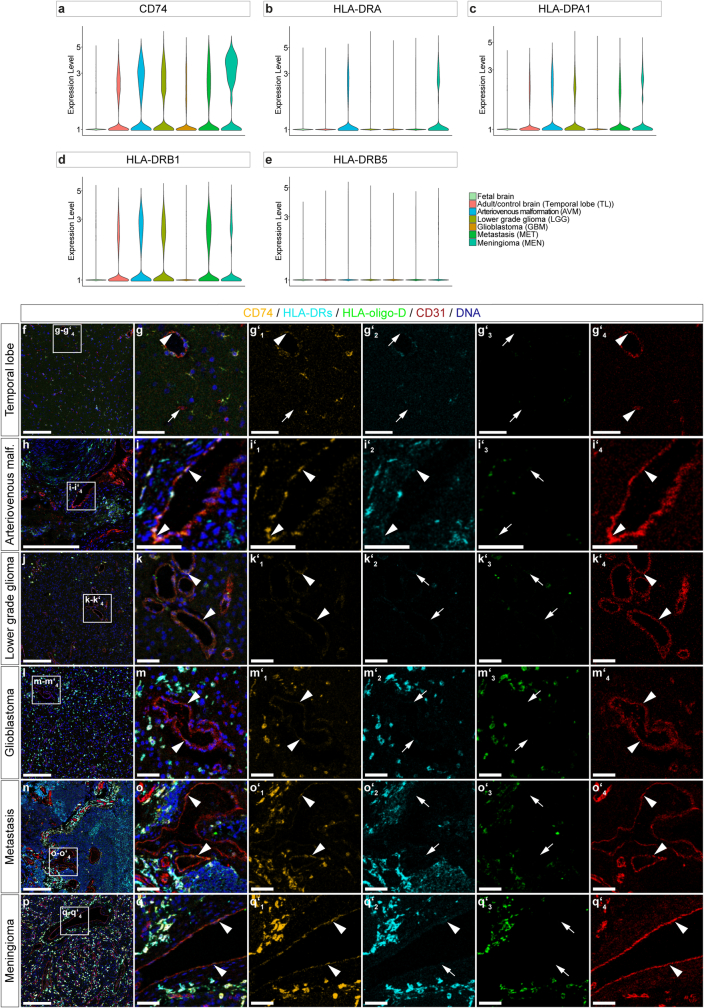
Validation of MHC class II signature markers in vascular ECs of the adult and pathological brain vasculature using IMC. **a**-**e**, Violin plots showing the expression of MHC class II marker genes in the indicated fetal brain (CNS), adult/control and pathological brain tissues. Markers shown were further validated by IF and/or IMC. **f-q‘**_**4**_ Mass cytometry (IMC) imaging of the indicated adult control and pathological brain tissue samples using metal-conjugated primary and secondary antibodies. Representative pseudocolor images of combined and individual channels of the different markers (white, overlap; orange, CD74; cyan, pan-HLA-DR; green, oligo-HLA-D; yellow, red, CD31), Scale bars: 250 μm in overviews, 50 μm in zooms. Shown are 5 antibodies stained for (Supplementary Table 24). Arrowheads identify blood vessels ECs in close contact with immune cell expressing the indicated markers in the different tissues. Arrows identify blood vessel ECs not in close contact with immune cell expressing the indicated markers.

**Extended Data Fig. 12:**
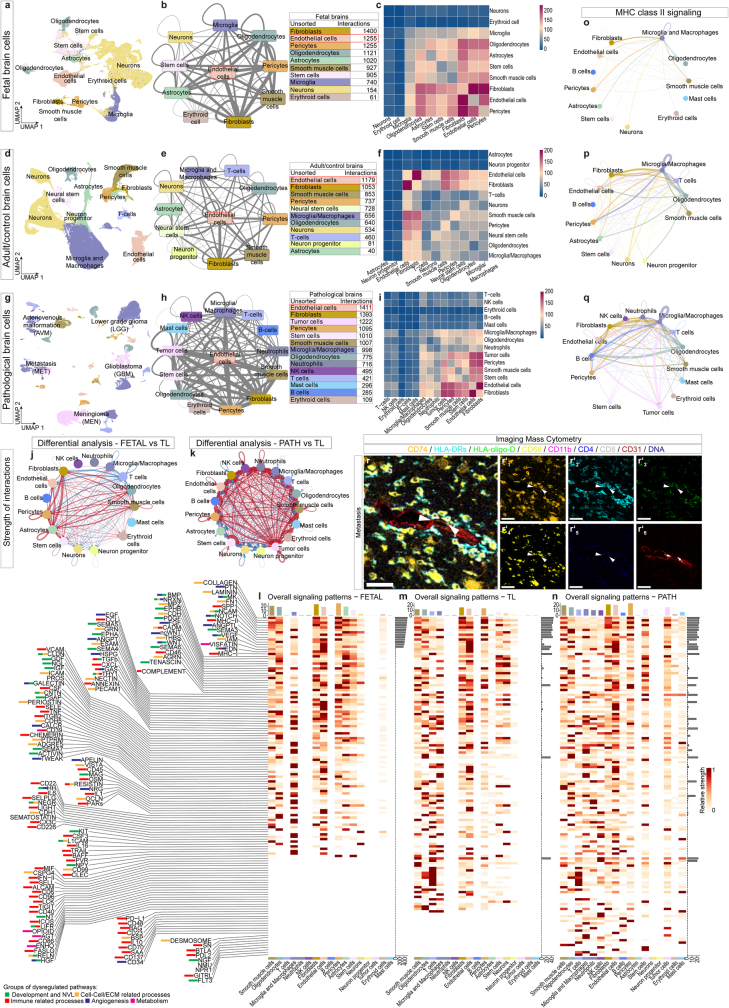
A key role for vascular ECs in the human brain neurovascular unit across development, adulthood and disease, highlighting MHC class II mediated interactions. **a**,**d**,**g**, UMAP plots of endothelial and perivascular cells derived from fetal brains (40,557 cells from 7 individuals) (**a**), adult/control brains (80,515 cells from 6 individuals) (**d**) and pathological brains (Arteriovenous malformations: 12,013 cells from 3 individuals; Lower-grade glioma: 20,712 cells from 4 individuals; Glioblastoma: 22,297 cells from 5 individuals; Metastasis: 9,204 cells from 3 individuals; Meningioma: 26,916 cells from 3 individuals) (**g**). **b**,**e**,**h**, Ligand and receptor analysis of fetal brain (**b**), adult control brain (**e**), and brain pathology (**h**) cells using CellphoneDB. Line thickness indicates the number of interactions between cell types. Tables summarize the number of interactions for each cell type. **c**,**f**,**i**, Heatmaps showing the number of ligand-receptor interactions between the different cells of fetal brains (**c**), adult/control brains (**f**), and pathological brains (**i**) computed using CellphoneDB. **j**,**k**, Circle plot showing differential analysis of strength of ligand receptor interactions in fetal over adult/control brains (**j**) and pathology over adult/control brains (**k**), red indicating upregulation, while blue indicating downregulation. **l**-**n**, Heatmap showing overall signalling patterns of different cell types in adult control (**o**) and pathological (**p**) brains. **o**,**p**,**q**, Circle plots showing the strength of MHC class II signalling interactions between the different cell types of fetal brain (**o**), adult/control brain (**p**), and pathological brain (**q**). **r-r’**_**6**_ Mass cytometry (IMC) imaging of metastasis tissue sample visualizing metal-conjugated CD74, pan-HLA-DR, oligo-HLA-D, CD68, CD11b, CD4, CD8 and CD31 primary antibodies and metal-conjugated secondary stains, a subset of the antibody set (Supplementary Table 24) stains present in all images. Overlay of pseudocolor images as well as individual channels for CD74, pan-HLA-DR, oligo-HLA-D, CD68, CD11b and CD4, (white, overlap; orange, CD74; cyan, pan-HLA-DR; green, oligo-HLA-D; yellow, CD68; purple, CD11b; blue, CD4; grey, CD8; red, CD31), Scale bars = 50 μm. Shown are 8 antibodies stained for. Arrowheads identify blood vessel ECs in close contact with immune cell expressing the indicated markers in the different tissues. Arrows identify blood vessel ECs not in close contact with immune cell expressing the indicated markers.

**Extended Data Fig. 13:**
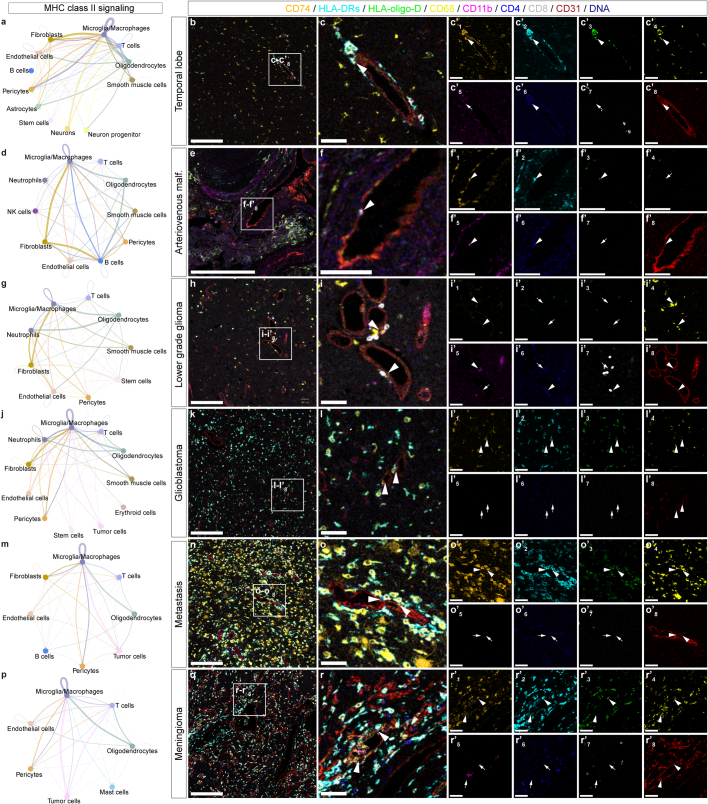
Validation of MHC class II mediated endothelial-immune cell interactions in the adult and pathological brain vasculature using IMC. **a**,**d**,**g**,**j**,**m**,**p**, Circle plots showing the strength of MHC class II signalling interactions between the different cell types of adult/control brain (**a**) arteriovenous malformation (**d**), lower-grade glioma (**g**), glioblastoma (**j**), metastasis (**m**) and meningioma (**p**) cells. **b-c’**_**8**_, **e-f’**_**8**_, **h-i’**_**8**_, **k-l’**_**8**_, **n-o’**_**8**_, **q-r’**_**8**_, Mass cytometry (IMC) imaging of the indicated adult control and pathological brain tissue samples using metal-conjugated CD74, pan-HLA-DR, oligo-HLA-D, CD68, CD11b, CD4, CD8 and CD31 primary antibodies and metal-conjugated secondary stains. Overlay of pseudocolor images as well as individual channels of the different markers (white, overlap; orange, CD74; cyan, pan-HLA-DR; green, oligo-HLA-D; yellow, CD68; purple, CD11b; blue, CD4; grey, CD8; red, CD31), Scale bars: 250 μm in overviews, 50 μm in zooms. Shown are 5 antibodies stained for (Supplementary Table 24). Arrowheads identify blood vessels ECs in close contact with immune cell expressing the indicated markers in the different tissues. Arrows identify blood vessel ECs not in close contact with immune cells expressing the indicated markers.

We confirmed enrichment of MHC class II genes including *CD74* and others in the pathological human cerebrovasculature using IF, IMC and RNAscope (Fig. 5h–w and Extended Data Figs 10, 11, 12r and 13).

Spatial transcriptomics confirmed spatial co-localization of MHC class II ligands and receptors on ECs in the temporal lobe and in GBM (Supplementary Fig. 18). MHC class II signalling seems to be mediated mainly by *APP*, *COPA* and *MIF* ligands and the *CD74* receptor in AV clusters (Supplementary Fig. 17), and *APP–CD74*, *COPA–CD74* and *MIF–CD74* have been described as ligand–receptor pairs^104,105^.

## A key role for ECs in the human brain NVU

Single-cell transcriptomics of unsorted ECs and PVCs offers the opportunity to address cellular cross-talk and the role of ECs within the NVU. To address cell–cell interactions across entities, we constructed ligand–receptor interaction maps^85,86^. In the majority of entities, ECs were at the centre of the network displaying numerous interactions with other ECs and PVCs (Extended Data Fig. 12a–i and Supplementary Fig. 19), indicating a crucial role of ECs in NVU function and EC–PVC cross-talk. In fetal and adult/control brains, ECs showed most interactions with fibroblasts, pericytes and astrocytes (Extended Data Fig. 12a–f). In brain pathologies, ECs displayed increased interaction numbers as well as increased interactions with immune cells (Extended Data Fig. 12g–i and Supplementary Figs. 19 and 20). Intercellular signalling pathways were substantially increased in fetal and pathological ECs (and PVCs) (Extended Data Fig. 12j,k and Supplementary Fig. 20a–g). Cell–cell communication analysis predicted upregulation of signalling pathways in the developing versus control brain as well as diseased versus control brain NVUs, including similar pathways as observed among EC clusters in EC–EC networks (Fig. 2i–k, Extended Data Fig. 12l–n and Supplementary Fig. 20k–n). Intercellular cross-talk analysis predicted a key role for the ECs within the fetal, adult/control and diseased brain NVU signalling networks (Extended Data Fig. 12l–n and Supplementary Fig. 20e–g).

During fetal brain development and in brain pathologies, we observed upregulation of ligands and receptors on ECs and PVCs as well as of the corresponding pathways, which partially overlapped with the ones in EC–EC cross-talk (Extended Data Fig. 12l–n), suggesting that these ligand–receptor interactions contribute to brain EC–PVC signalling.

On the basis of our observation of MHC class II signalling in EC–EC communication, we next addressed MHC class II signalling in EC–PVC intercellular cross-talk, which predicted elevated MHC class II signalling (predominantly in microglia and macrophages, ECs and tumour cells/oligodendrocytes) in brain pathologies (Extended Data Fig. 12o,p,q and Supplementary Fig. 20h–j). Spatial transcriptomics confirmed spatial co-localization of MHC class II ligands and receptors on ECs and PVCs (Supplementary Fig. 18) in the temporal lobe and in GBM, while IMC illustrated physical proximity between MHC class II-expressing ECs and microglia/macrophages across all entities (Extended Data Figs. 12r and 13). Notably, the *APP–CD74*, *COPA–CD74* and *MIF–CD74* ligand–receptor pairs that were predicted to mediate MHC class II signalling in EC–EC interactions were also predicted ligand–receptor pairs in the developing, adult and diseased NVU (Supplementary Fig. 20h–j), with ECs notably strongly expressing *CD74* (Supplementary Fig. 19e,j,o,t,y). These data suggest involvement of MHC class II in EC–immune cell interactions and indicate that the *APP–CD74*, *COPA–CD74* and *MIF–CD74* ligand–receptor pairs contribute to NVU signalling.

## Discussion

Here we generated a large-scale single-cell molecular atlas of the developing fetal, adult/control and diseased human brain vasculature at a very high resolution, using scRNA-seq, composed of 606,308 freshly isolated endothelial, perivascular and other tissue-derived cells covering a substantial diversity of human brain tissue.

We have provided molecular definitions of human brain cell types and their differences by brain developmental stage and pathology, thereby unravelling organizational principles of ECs and PVCs composing the human brain vasculature. Our experimental methodology relies on transcriptional profiles of human cerebrovascular cells generated from fresh human neurosurgical resections and fresh fetal abortions, reducing the likelihood of transcriptional alterations associated with post-mortem tissue acquisition (Supplementary Discussion).

Our human vascular brain atlas provides a basis for understanding the organizing principles and single-cell heterogeneity of universal, specialized and activated endothelial and PVCs with broad implications for physiology and medicine, and serves as a powerful publicly available reference for the field.

### Reporting summary

Further information on research design is available in the Nature Portfolio Reporting Summary linked to this article.

## Online content

Any methods, additional references, Nature Portfolio reporting summaries, source data, extended data, supplementary information, acknowledgements, peer review information; details of author contributions and competing interests; and statements of data and code availability are available at 10.1038/s41586-024-07493-y.

## Supplementary information

Supplementary Information Supplementary Figs. 1–20.

Reporting Summary

Peer Review File

Supplementary Methods A detailed description of the methods used in our article with a separate references section.

Supplementary Discussion Supplementary Discussion.

Supplementary Tables 1–27 Supplementary Tables 1–27 and the legends for the Supplementary Tables.

## Figures and Tables

**Extended Data Fig. 7:**
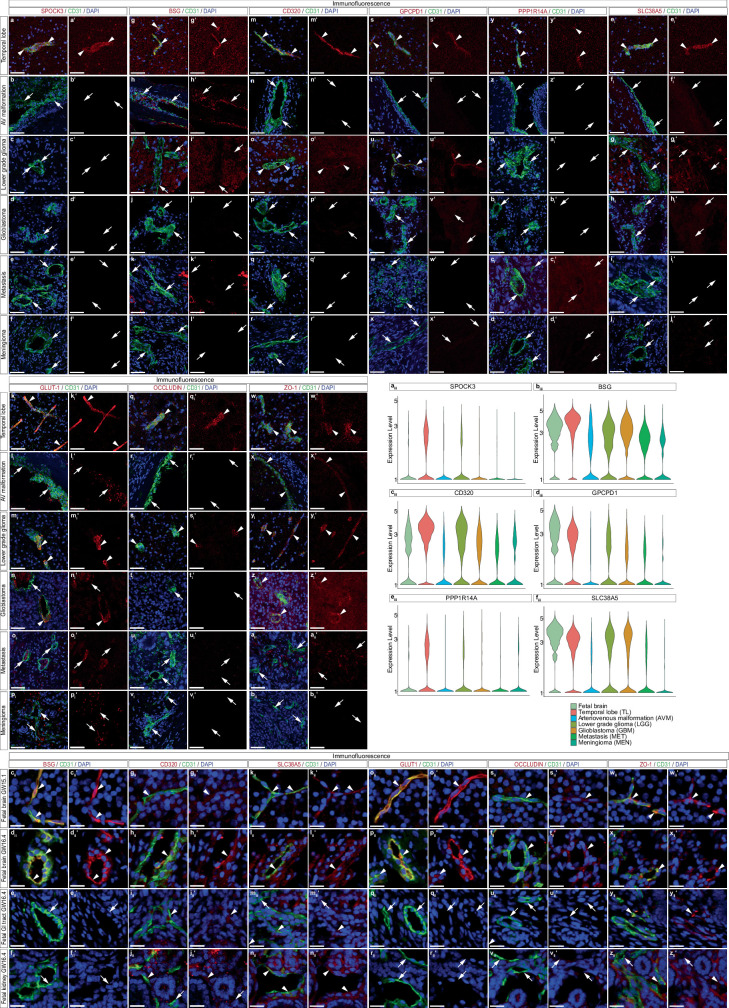
Validation of CNS signature markers in vascular ECs of the developing fetal, adult and pathological brain vasculature using IF. **a-z**_**ii**_**‘**, Immunofluorescence (IF) imaging of tissue sections from the indicated entities, stained for *SPOCK3* (red; **a-f‘**), *BSG* (red; **g-l‘**, **c**_**ii**_**-f**_**ii**_**‘**), *CD320* (red; **m-r‘**, **g**_**ii**_**-j**_**ii**_**‘**), *GPCPD1* (red; **s-x‘**), *PPP1R141* (red; **y-d**_**i**_**‘**), *SLC38A5* (red; **e**_**i**_**-j**_**i**_**‘**, **k**_**ii**_**-n**_**ii**_**‘**), *GLUT1* (red; **k**_**i**_**‘-p**_**i**_**‘**, **o**_**ii**_**-r**_**ii**_**‘**), *OCLN* (red; **q**_**i**_**‘-v**_**i**_**‘**, **s**_**ii**_**-v**_**ii**_**‘**), *ZO-1* (red; **w**_**i**_**-b**_**ii**_**‘**, **w**_**ii**_**-z**_**ii**_**‘**) and *CD31* (green). Nuclei are stained with DAPI (blue). Arrowheads identify blood vessel ECs expressing the indicated markers in the different tissues. Arrows identify blood vessel ECs not expressing the indicated markers in the different tissues. Scale bars: 200 μm in overviews, 50 μm in zooms. **a**_**iii**_**-f**_**iii**_, Violin plots showing the expression of CNS-specific genes in the indicated adult control and pathological brain tissues.

**Extended Data Fig. 8:**
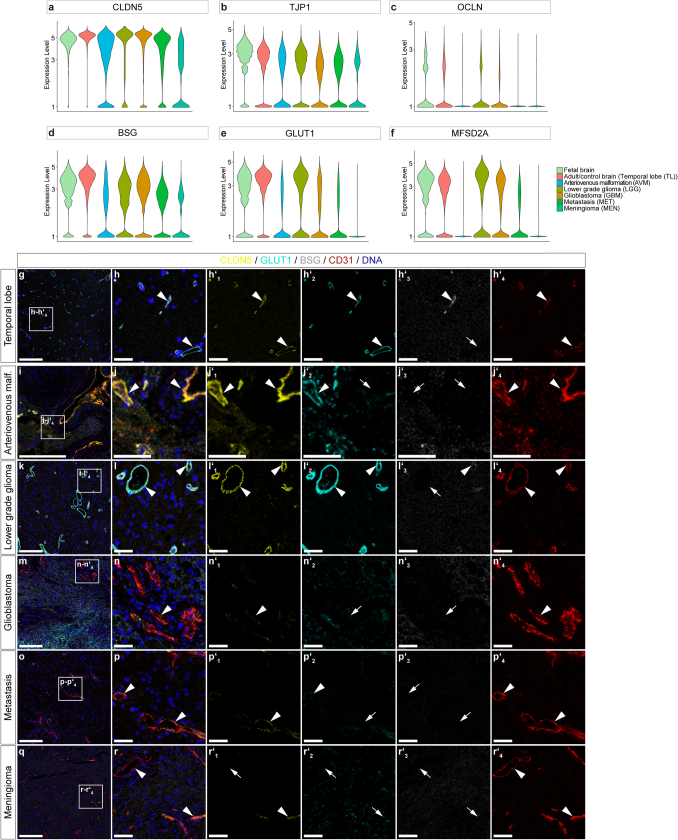
Validation of CNS signature markers in vascular ECs of the developing fetal, adult and pathological brain vasculature using IMC. **a**-**f**, Violin plots showing the expression of CNS-specific and BBB marker genes in the indicated adult control and pathological brain tissues. Markers were further validated by IF and/or IMC. **g-r‘**_**4**_ Mass cytometry (IMC) imaging of the indicated adult control and pathological brain tissue samples using metal-conjugated antibodies. Representative pseudocolour images of combined and individual channels of the different markers (white, overlap; yellow, CLDN5; cyan, GLUT1; grey, BSG; red, CD31; blue, DNA intercalator), Scale bars: 250 μm in overviews, 50 μm in zooms. Shown are 5 antibodies stained for (Supplementary Table 24). Arrowheads identify blood vessels ECs expressing the indicated markers in the different tissues. Arrows identify blood vessel ECs not expressing the indicated markers in the different tissues.

## Data Availability

All data are now accessible under Gene Expression Omnibus accession number GSE256493. An interactive website is available at https://waelchli-lab-human-brain-vasculature-atlas.ethz.ch, https://brain-vasc.cells.ucsc.edu and https://cellxgene.cziscience.com/collections/c95ca269-68a7-47c5-82db-da227f31b598 on which the data can visualized and downloaded.

## Code availability

Most of our analyses are standard workflows and we have now deposited the source code at GitHub (https://github.com/Waelchli-lab/Single-cell-atlas-of-the-human-brain-vasculature-accross-development-adulthood-and-disease) to improve reproducibility our results. The generated Seurat objects of FACS-sorted (CD31^+^CD45^−^) ECs for the individual entities can be downloaded from 10.5281/zenodo.10058183, and for the overall merges from 10.5281/zenodo.10057779. The generated Seurat objects of unsorted ECs and PVCs for individual entities can be downloaded from 10.5281/zenodo.10058371, and for the overall merges from 10.5281/zenodo.10058563. The generated Monocle 3 CDS pseudotime objects of FACS-sorted (CD31^+^CD45^−^) ECs can be downloaded from 10.5281/zenodo.10058766. The generated diffusion map objects of FACS-sorted (CD31^+^CD45^−^) ECs can be downloaded from 10.5281/zenodo.10060876. The generated RNA velocity objects of FACS-sorted (CD31^+^CD45^−^) ECs of individual pathological entities can be downloaded from 10.5281/zenodo.10065659; the fetal and adult/control brains from 10.5281/zenodo.10066390; the overall merge of brain tumours from 10.5281/zenodo.10066538; and the overall merge of pathological entities from 10.5281/zenodo.10066703.

## References

[1] Wälchli, T. et al. Shaping the brain vasculature in development and disease in the single-cell era. *Nat. Rev. Neurosci.* **24**, 271–298 (2023). 36941369 10.1038/s41583-023-00684-y PMC10026800

[2] Cho, C., Smallwood, P. M. & Nathans, J. Reck and Gpr124 are essential receptor cofactors for Wnt7a/Wnt7b-Specific signaling in mammalian CNS angiogenesis and blood-brain barrier regulation. *Neuron* **95**, 1221–1225 (2017). 28858622 10.1016/j.neuron.2017.08.032

[3] Wälchli, T. et al. Wiring the vascular network with neural cues: a CNS perspective. *Neuron* **87**, 271–296 (2015). 26182414 10.1016/j.neuron.2015.06.038

[4] Sweeney, M. D., Kisler, K., Montagne, A., Toga, A. W. & Zlokovic, B. V. The role of brain vasculature in neurodegenerative disorders. *Nat. Neurosci.* **21**, 1318–1331 (2018). 30250261 10.1038/s41593-018-0234-x PMC6198802

[5] Zlokovic, B. V. The blood-brain barrier in health and chronic neurodegenerative disorders. *Neuron* **57**, 178–201 (2008). 18215617 10.1016/j.neuron.2008.01.003

[6] Kuhnert, F. et al. Essential regulation of CNS angiogenesis by the orphan G protein-coupled receptor GPR124. *Science* **330**, 985–989 (2010). 21071672 10.1126/science.1196554 PMC3099479

[7] Chang, J. et al. Gpr124 is essential for blood-brain barrier integrity in central nervous system disease. *Nat. Med.* **23**, 450–460 (2017). 28288111 10.1038/nm.4309 PMC5559385

[8] Anderson, K. D. et al. Angiogenic sprouting into neural tissue requires Gpr124, an orphan G protein-coupled receptor. *Proc. Natl Acad. Sci. USA* **108**, 2807–2812 (2011). 21282641 10.1073/pnas.1019761108 PMC3041062

[9] Zhou, Y. & Nathans, J. Gpr124 controls CNS angiogenesis and blood-brain barrier integrity by promoting ligand-specific canonical wnt signaling. *Dev. Cell* **31**, 248–256 (2014). 25373781 10.1016/j.devcel.2014.08.018 PMC4223636

[10] Carmeliet, P. Angiogenesis in health and disease. *Nat. Med.* **9**, 653–660 (2003). 12778163 10.1038/nm0603-653

[11] Carmeliet, P. & Jain, R. K. Molecular mechanisms and clinical applications of angiogenesis. *Nature* **473**, 298–307 (2011). 21593862 10.1038/nature10144 PMC4049445

[12] Potente, M., Gerhardt, H. & Carmeliet, P. Basic and therapeutic aspects of angiogenesis. *Cell* **146**, 873–887 (2011). 21925313 10.1016/j.cell.2011.08.039

[13] Carmeliet, P. Angiogenesis in life, disease and medicine. *Nature* **438**, 932–936 (2005). 16355210 10.1038/nature04478

[14] Quaegebeur, A., Lange, C. & Carmeliet, P. The neurovascular link in health and disease: molecular mechanisms and therapeutic implications. *Neuron* **71**, 406–424 (2011). 21835339 10.1016/j.neuron.2011.07.013

[15] Ghobrial, M. The human brain vasculature shows a distinct expression pattern of SARS-CoV-2 entry factors. Preprint at *bioRxiv*10.1101/2020.10.10.334664 (2020).

[16] Wälchli, T. et al. Quantitative assessment of angiogenesis, perfused blood vessels and endothelial tip cells in the postnatal mouse brain. *Nat. Protoc.* **10**, 53–74 (2015). 10.1038/nprot.2015.002 25502884

[17] Wälchli, T. et al. Hierarchical imaging and computational analysis of three-dimensional vascular network architecture in the entire postnatal and adult mouse brain. *Nat. Protoc.* **16**, 4564–4610 (2021). 34480130 10.1038/s41596-021-00587-1

[18] Nikolaev, S. I. et al. Somatic activating KRAS mutations in arteriovenous malformations of the brain. *N. Engl. J. Med.* **378**, 250–261 (2018). 29298116 10.1056/NEJMoa1709449 PMC8161530

[19] Wälchli, T. et al. Nogo-A is a negative regulator of CNS angiogenesis. *Proc. Natl Acad. Sci. USA* **110**, E1943–E1952 (2013). 23625008 10.1073/pnas.1216203110 PMC3666688

[20] Wälchli, T. et al. Nogo-A regulates vascular network architecture in the postnatal brain. *J. Cereb. Blood Flow Metab.* **37**, 614–631 (2017). 27927704 10.1177/0271678X16675182 PMC5381465

[21] Schwab, M. et al. Nucleolin promotes angiogenesis and endothelial metabolism along the oncofetal axis in the human brain vasculature. *JCI Insight* **8**, e143071 (2023). 36917178 10.1172/jci.insight.143071 PMC10243742

[22] Arvanitis, C. D., Ferraro, G. B. & Jain, R. K. The blood-brain barrier and blood-tumour barrier in brain tumours and metastases. *Nat. Rev. Cancer* **20**, 26–41 (2020). 31601988 10.1038/s41568-019-0205-x PMC8246629

[23] Engelhardt, B. Development of the blood-brain barrier. *Cell Tissue Res.* **314**, 119–129 (2003). 12955493 10.1007/s00441-003-0751-z

[24] Engelhardt, B. Blood-brain barrier differentiation. *Science* **334**, 1652–1653 (2011). 22194564 10.1126/science.1216853

[25] Mancuso, M. R., Kuhnert, F. & Kuo, C. J. Developmental angiogenesis of the central nervous system. *Lymphat. Res. Biol.* **6**, 173–180 (2008). 19093790 10.1089/lrb.2008.1014 PMC2712664

[26] Daneman, R. et al. Wnt/β-catenin signaling is required for CNS, but not non-CNS, angiogenesis. *Proc. Natl Acad. Sci. USA* **106**, 641–646 (2009). 19129494 10.1073/pnas.0805165106 PMC2626756

[27] Liebner, S. et al. Wnt/β-catenin signaling controls development of the blood-brain barrier. *J. Cell Biol.* **183**, 409–417 (2008). 18955553 10.1083/jcb.200806024 PMC2575783

[28] Vanlandewijck, M. et al. A molecular atlas of cell types and zonation in the brain vasculature. *Nature* **554**, 475–480 (2018). 29443965 10.1038/nature25739

[29] Majno, G. & Palade, G. E. Studies on inflammation. 1. The effect of histamine and serotonin on vascular permeability: an electron microscopic study. *J. Biophys. Biochem. Cytol.* **11**, 571–605 (1961). 14468626 10.1083/jcb.11.3.571 PMC2225138

[30] Simionescu, M., Simionescu, N. & Palade, G. E. Segmental differentiations of cell junctions in the vascular endothelium. The microvasculature. *J. Cell Biol.* **67**, 863–885 (1975). 1202025 10.1083/jcb.67.3.863 PMC2111645

[31] Han, X. et al. Construction of a human cell landscape at single-cell level. *Nature* **581**, 303–309 (2020). 32214235 10.1038/s41586-020-2157-4

[32] Litviňuková, M. et al. Cells of the adult human heart. *Nature* **588**, 466–472 (2020). 32971526 10.1038/s41586-020-2797-4 PMC7681775

[33] Travaglini, K. J. et al. A molecular cell atlas of the human lung from single-cell RNA sequencing. *Nature* **587**, 619–625 (2020). 33208946 10.1038/s41586-020-2922-4 PMC7704697

[34] Garcia, F. J. et al. Single-cell dissection of the human brain vasculature. *Nature* **603**, 893–899 (2022). 35158371 10.1038/s41586-022-04521-7 PMC9680899

[35] Yang, A. C. et al. A human brain vascular atlas reveals diverse mediators of Alzheimer’s risk. *Nature* **603**, 885–892 (2022). 35165441 10.1038/s41586-021-04369-3 PMC9635042

[36] Winkler, E. A. et al. A single-cell atlas of the normal and malformed human brain vasculature. *Science* **375**, eabi7377 (2022). 35084939 10.1126/science.abi7377 PMC8995178

[37] Crouch, E. E. et al. Ensembles of endothelial and mural cells promote angiogenesis in prenatal human brain. *Cell* **185**, 3753–3769 (2022). 36179668 10.1016/j.cell.2022.09.004 PMC9550196

[38] Kalucka, J. et al. Single-cell transcriptome atlas of murine endothelial cells. *Cell* **180**, 764–779 (2020). 32059779 10.1016/j.cell.2020.01.015

[39] Marin-Padilla, M. The human brain intracerebral microvascular system: development and structure. *Front. Neuroanat.* **6**, 38 (2012). 22993505 10.3389/fnana.2012.00038 PMC3440694

[40] Saunders, N. R., Liddelow, S. A. & Dziegielewska, K. M. Barrier mechanisms in the developing brain. *Front. Pharmacol.* **3**, 46 (2012). 22479246 10.3389/fphar.2012.00046 PMC3314990

[41] Saunders, N. R., Dziegielewska, K. M., Mollgard, K. & Habgood, M. D. Physiology and molecular biology of barrier mechanisms in the fetal and neonatal brain. *J. Physiol.* **596**, 5723–5756 (2018). 29774535 10.1113/JP275376 PMC6265560

[42] Jabbour, P. M., Tjoumakaris, S. I. & Rosenwasser, R. H. Endovascular management of intracranial aneurysms. *Neurosurg. Clin. N. Am.* **20**, 383–398 (2009). 19853799 10.1016/j.nec.2009.07.003

[43] Xu, R., Pisapia, D. & Greenfield, J. P. Malignant transformation in glioma steered by an angiogenic switch: defining a role for bone marrow-derived cells. *Cureus* **8**, e471 (2016). 26973806 10.7759/cureus.471 PMC4772998

[44] Jain, R. K. et al. Angiogenesis in brain tumours. *Nat. Rev. Neurosci.* **8**, 610–622 (2007). 17643088 10.1038/nrn2175

[45] Das, S. & Marsden, P. A. Angiogenesis in glioblastoma. *N. Engl. J. Med.* **369**, 1561–1563 (2013). 24131182 10.1056/NEJMcibr1309402 PMC5378489

[46] Lorger, M., Krueger, J. S., O’Neal, M., Staflin, K. & Felding-Habermann, B. Activation of tumor cell integrin alphavbeta3 controls angiogenesis and metastatic growth in the brain. *Proc. Natl Acad. Sci. USA* **106**, 10666–10671 (2009). 19541645 10.1073/pnas.0903035106 PMC2697113

[47] Barresi, V. Angiogenesis in meningiomas. *Brain Tumor Pathol.* **28**, 99–106 (2011). 21290262 10.1007/s10014-010-0012-2

[48] Picelli, S. et al. Full-length RNA-seq from single cells using Smart-seq2. *Nat. Protoc.* **9**, 171–181 (2014). 24385147 10.1038/nprot.2014.006

[49] Xie, Y. et al. Key molecular alterations in endothelial cells in human glioblastoma uncovered through single-cell RNA sequencing. *JCI Insight* **6**, e150861 (2021). 34228647 10.1172/jci.insight.150861 PMC8410070

[50] Parab, S., Quick, R. E. & Matsuoka, R. L. Endothelial cell-type-specific molecular requirements for angiogenesis drive fenestrated vessel development in the brain. *eLife* **10**, e64295 (2021). 33459592 10.7554/eLife.64295 PMC7840183

[51] Wisniewska-Kruk, J. et al. Plasmalemma vesicle-associated protein has a key role in blood-retinal barrier loss. *Am. J. Pathol.* **186**, 1044–1054 (2016). 26878208 10.1016/j.ajpath.2015.11.019

[52] Bosma, E. K., van Noorden, C. J. F., Schlingemann, R. O. & Klaassen, I. The role of plasmalemma vesicle-associated protein in pathological breakdown of blood-brain and blood-retinal barriers: potential novel therapeutic target for cerebral edema and diabetic macular edema. *Fluids Barriers CNS* **15**, 24 (2018). 30231925 10.1186/s12987-018-0109-2 PMC6146740

[53] Carson-Walter, E. B. et al. Plasmalemmal vesicle associated protein-1 is a novel marker implicated in brain tumor angiogenesis. *Clin. Cancer Res.* **11**, 7643–7650 (2005). 16278383 10.1158/1078-0432.CCR-05-1099

[54] Zhang, H. et al. Targeting endothelial cell-specific molecule 1 protein in cancer: a promising therapeutic approach. *Front. Oncol.* **11**, 687120 (2021). 34109132 10.3389/fonc.2021.687120 PMC8181400

[55] McCracken, I. R. et al. Transcriptional dynamics of pluripotent stem cell-derived endothelial cell differentiation revealed by single-cell RNA sequencing. *Eur. Heart J.* **41**, 1024–1036 (2020). 31242503 10.1093/eurheartj/ehz351 PMC9597329

[56] Dieterich, L. C. et al. Transcriptional profiling of human glioblastoma vessels indicates a key role of VEGF-A and TGFβ2 in vascular abnormalization. *J. Pathol.* **228**, 378–390 (2012). 22786655 10.1002/path.4072

[57] Huang, Z. et al. Effects of sex and aging on the immune cell landscape as assessed by single-cell transcriptomic analysis. *Proc. Natl Acad. Sci. USA* **118**, e2023216118 (2021). 34385315 10.1073/pnas.2023216118 PMC8379935

[58] Huang, X. et al. Single-cell transcriptional profiling reveals sex and age diversity of gene expression in mouse endothelial cells. *Front. Genet.* **12**, 590377 (2021). 33679877 10.3389/fgene.2021.590377 PMC7929607

[59] Hajdarovic, K. H. et al. Single-cell analysis of the aging female mouse hypothalamus. *Nat. Aging* **2**, 662–678 (2022). 36285248 10.1038/s43587-022-00246-4 PMC9592060

[60] Hao, Y. et al. Integrated analysis of multimodal single-cell data. *Cell* **184**, 3573–3587 (2021). 34062119 10.1016/j.cell.2021.04.048 PMC8238499

[61] Stuart, T. et al. Comprehensive integration of single-cell data. *Cell* **177**, 1888–1902 (2019). 31178118 10.1016/j.cell.2019.05.031 PMC6687398

[62] Butler, A., Hoffman, P., Smibert, P., Papalexi, E. & Satija, R. Integrating single-cell transcriptomic data across different conditions, technologies, and species. *Nat. Biotechnol.* **36**, 411–420 (2018). 29608179 10.1038/nbt.4096 PMC6700744

[63] Satija, R., Farrell, J. A., Gennert, D., Schier, A. F. & Regev, A. Spatial reconstruction of single-cell gene expression data. *Nat. Biotechnol.* **33**, 495–502 (2015). 25867923 10.1038/nbt.3192 PMC4430369

[64] Yang, A. C. et al. A human brain vascular atlas reveals diverse mediators of Alzheimer's risk. *Nature* **603**, 885–892 (2022). 10.1038/s41586-021-04369-3 PMC9635042 35165441

[65] Yang, A. C. et al. Physiological blood–brain transport is impaired with age by a shift in transcytosis. *Nature* **583**, 425–430 (2020). 32612231 10.1038/s41586-020-2453-z PMC8331074

[66] Chen, M. B. et al. Brain endothelial cells are exquisite sensors of age-related circulatory cues. *Cell Rep.* **30**, 4418–4432 (2020). 32234477 10.1016/j.celrep.2020.03.012 PMC7292569

[67] Halpern, K. B. et al. Single-cell spatial reconstruction reveals global division of labour in the mammalian liver. *Nature* **542**, 352–356 (2017). 28166538 10.1038/nature21065 PMC5321580

[68] He, L. et al. Analysis of the brain mural cell transcriptome. *Sci. Rep.* **6**, 35108 (2016). 27725773 10.1038/srep35108 PMC5057134

[69] Garcia, F. J. et al. Single-cell dissection of the human brain vasculature. *Nature* **603**, 893–899 (2022). 10.1038/s41586-022-04521-7 PMC9680899 35158371

[70] Schaum, N. et al. Single-cell transcriptomics of 20 mouse organs creates a *Tabula Muris*. *Nature* **562**, 367–372 (2018). 30283141 10.1038/s41586-018-0590-4 PMC6642641

[71] Platel, V., Faure, S., Corre, I. & Clere, N. Endothelial-to-mesenchymal transition (EndoMT): roles in tumorigenesis, metastatic extravasation and therapy resistance. *J. Oncol.* **2019**, 8361945–8361945 (2019). 31467544 10.1155/2019/8361945 PMC6701373

[72] Suvà, M. L. & Tirosh, I. The glioma stem cell model in the era of single-cell genomics. *Cancer Cell* **37**, 630–636 (2020). 32396858 10.1016/j.ccell.2020.04.001

[73] Suvà, M. L. et al. Reconstructing and reprogramming the tumor-propagating potential of glioblastoma stem-like cells. *Cell* **157**, 580–594 (2014). 24726434 10.1016/j.cell.2014.02.030 PMC4004670

[74] Wang, R. et al. Glioblastoma stem-like cells give rise to tumour endothelium. *Nature* **468**, 829–833 (2010). 21102433 10.1038/nature09624

[75] Ricci-Vitiani, L. et al. Tumour vascularization via endothelial differentiation of glioblastoma stem-like cells. *Nature* **468**, 824–828 (2010). 21102434 10.1038/nature09557

[76] Prabavathy, D., Swarnalatha, Y. & Ramadoss, N. Lung cancer stem cells-origin, characteristics and therapy. *Stem Cell Invest.* **5**, 6 (2018). 10.21037/sci.2018.02.01 PMC5897668 29682513

[77] Lawton, M. T. et al. Brain arteriovenous malformations. *Nat. Rev. Dis. Primers* **1**, 15008 (2015). 27188382 10.1038/nrdp.2015.8

[78] Malinverno, M. et al. Endothelial cell clonal expansion in the development of cerebral cavernous malformations. *Nat. Commun.* **10**, 2761 (2019). 31235698 10.1038/s41467-019-10707-x PMC6591323

[79] Orsenigo, F. et al. Mapping endothelial-cell diversity in cerebral cavernous malformations at single-cell resolution. *eLife* **9**, e61413 (2020). 33138917 10.7554/eLife.61413 PMC7609066

[80] Zhu, I. et al. Modular design of synthetic receptors for programmed gene regulation in cell therapies. *Cell* **185**, 1431–1443 (2022). 35427499 10.1016/j.cell.2022.03.023 PMC9108009

[81] Uhlen, M. et al. Tissue-based map of the human proteome. *Science* **347**, 1260419 (2015). 25613900 10.1126/science.1260419

[82] Tang, M. et al. Evaluating single-cell cluster stability using the Jaccard similarity index. *Bioinformatics* **37**, 2212–2214 (2021). 33165513 10.1093/bioinformatics/btaa956 PMC8352506

[83] Sjostedt, E. et al. An atlas of the protein-coding genes in the human, pig, and mouse brain. *Science* **367**, eaay5947 (2020). 32139519 10.1126/science.aay5947

[84] Hodge, R. D. et al. Conserved cell types with divergent features in human versus mouse cortex. *Nature* **573**, 61–68 (2019). 31435019 10.1038/s41586-019-1506-7 PMC6919571

[85] Jin, S. et al. Inference and analysis of cell-cell communication using CellChat. *Nat. Commun.* **12**, 1088 (2021). 33597522 10.1038/s41467-021-21246-9 PMC7889871

[86] Efremova, M., Vento-Tormo, M., Teichmann, S. A. & Vento-Tormo, R. CellPhoneDB: inferring cell–cell communication from combined expression of multi-subunit ligand–receptor complexes. *Nat. Protoc.* **15**, 1484–1506 (2020). 32103204 10.1038/s41596-020-0292-x

[87] Farnsworth, R. H., Lackmann, M., Achen, M. G. & Stacker, S. A. Vascular remodeling in cancer. *Oncogene* **33**, 3496–3505 (2014). 23912450 10.1038/onc.2013.304

[88] Trapnell, C. et al. The dynamics and regulators of cell fate decisions are revealed by pseudotemporal ordering of single cells. *Nat. Biotechnol.* **32**, 381–386 (2014). 24658644 10.1038/nbt.2859 PMC4122333

[89] Ji, Z. & Ji, H. TSCAN: tools for single-cell analysis. R package v.1.34.0 (2022).

[90] La Manno, G. et al. RNA velocity of single cells. *Nature* **560**, 494–498 (2018). 30089906 10.1038/s41586-018-0414-6 PMC6130801

[91] Bergen, V., Lange, M., Peidli, S., Wolf, F. A. & Theis, F. J. Generalizing RNA velocity to transient cell states through dynamical modeling. *Nat. Biotechnol.* **38**, 1408–1414 (2020). 32747759 10.1038/s41587-020-0591-3

[92] Angerer, P. et al. destiny: diffusion maps for large-scale single-cell data in R. *Bioinformatics* **32**, 1241–1243 (2016). 26668002 10.1093/bioinformatics/btv715

[93] Munji, R. N. et al. Profiling the mouse brain endothelial transcriptome in health and disease models reveals a core blood–brain barrier dysfunction module. *Nat. Neurosci.* **22**, 1892–1902 (2019). 31611708 10.1038/s41593-019-0497-x PMC6858546

[94] Saili, K. S. et al. Blood-brain barrier development: systems modeling and predictive toxicology. *Birth Defects Res.* **109**, 1680–1710 (2017). 29251840 10.1002/bdr2.1180 PMC6476421

[95] Saunders, N. R. et al. The rights and wrongs of blood-brain barrier permeability studies: a walk through 100 years of history. *Front. Neurosci.* **8**, 404 (2014). 25565938 10.3389/fnins.2014.00404 PMC4267212

[96] Virgintino, D. et al. Immunolocalization of tight junction proteins in the adult and developing human brain. *Histochem. Cell Biol.* **122**, 51–59 (2004). 15221411 10.1007/s00418-004-0665-1

[97] Daneman, R. & Prat, A. The blood-brain barrier. *Cold Spring Harb. Perspect. Biol.* **7**, a020412 (2015). 25561720 10.1101/cshperspect.a020412 PMC4292164

[98] Ben-Zvi, A. et al. Mfsd2a is critical for the formation and function of the blood–brain barrier. *Nature* **509**, 507–511 (2014). 24828040 10.1038/nature13324 PMC4134871

[99] Ma, S. C. et al. Claudin-5 regulates blood-brain barrier permeability by modifying brain microvascular endothelial cell proliferation, migration, and adhesion to prevent lung cancer metastasis. *CNS Neurosci. Ther.* **23**, 947–960 (2017). 28961379 10.1111/cns.12764 PMC6492739

[100] Goveia, J. et al. An integrated gene expression landscape profiling approach to identify lung tumor endothelial cell heterogeneity and angiogenic candidates. *Cancer Cell* **37**, 21–36 (2020). 31935371 10.1016/j.ccell.2019.12.001

[101] Andreone, B. J. et al. Blood-brain barrier permeability is regulated by lipid transport-dependent suppression of caveolae-mediated transcytosis. *Neuron* **94**, 581–594 (2017). 28416077 10.1016/j.neuron.2017.03.043 PMC5474951

[102] O’Brown, N. M., Megason, S. G. & Gu, C. Suppression of transcytosis regulates zebrafish blood-brain barrier function. *eLife* **8**, e47326 (2019). 31429822 10.7554/eLife.47326 PMC6726461

[103] Zhao, Z., Nelson, A. R., Betsholtz, C. & Zlokovic, B. V. Establishment and dysfunction of the blood-brain barrier. *Cell* **163**, 1064–1078 (2015). 26590417 10.1016/j.cell.2015.10.067 PMC4655822

[104] Kim, N. et al. Single-cell RNA sequencing demonstrates the molecular and cellular reprogramming of metastatic lung adenocarcinoma. *Nat. Commun.* **11**, 2285 (2020). 32385277 10.1038/s41467-020-16164-1 PMC7210975

[105] Jiang, Y. Q. et al. Investigating mechanisms of response or resistance to immune checkpoint inhibitors by analyzing cell-cell communications in tumors before and after programmed cell death-1 (PD-1) targeted therapy: an integrative analysis using single-cell RNA and bulk-RNA sequencing data. *Oncoimmunology* **10**, 1908010 (2021). 33868792 10.1080/2162402X.2021.1908010 PMC8023241

