# Supplementary material for: Single-cell atlas of the human brain vasculature across development, adulthood and disease
Source: Nature. 2024 Jul 10;632(8025):603–13. doi: 10.1038/s41586-024-07493-y (PMC11324530; doi:10.1038/s41586-024-07493-y)
Supplement: Supplementary file 1 — Supplementary Figs. 1–20. [file 41586_2024_7493_MOESM1_ESM.pdf]

nature portfolio

https://doi.org/10.1038/s41586-024-07493-y

Supplementary information

Single-cell atlas of the human brain vasculature across development, adulthood and disease

In the format provided by the authors and unedited

Supplementary Figure 1

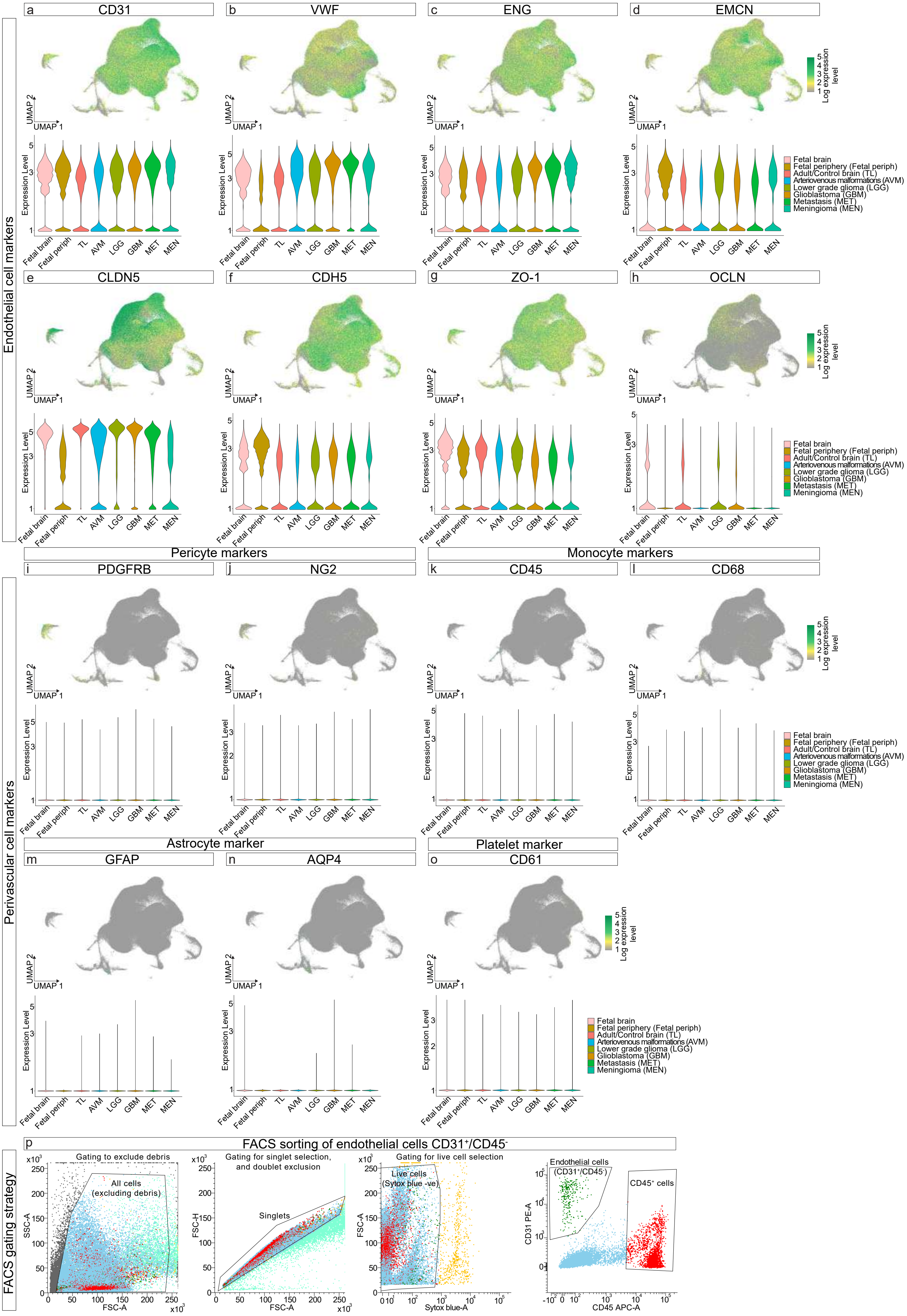

Supplementary Figure 1 | Validation of endothelial identity of $CD31^{+}/CD45^{-}$ FACS-sorted endothelial cells in the developing fetal, adult and pathological human brain vasculature, and in the developing fetal peripheral vasculature

a-o, UMAP plot of the 304,016 integrated/batch corrected ECs from fetal brain, fetal periphery, adult/control brain and pathological brain across 5 fetal brain, 8 fetal periphery, 9 adult/control and 29 pathological individuals (Supplementary Table 3), and violin plots showing the expression of endothelial (**a-h**) and perivascular (**i-o**) markers in the isolated endothelial cells from fetal periphery and fetal, adult/control and pathological brains. **p,** FACS sorting of endothelial cells ($CD31^{+}/CD45^{-}$), plots show the gating strategy: cell debris were excluded via a forward scatter-area/side scatter-area (FSC-A/SSC-A) gating, while singlets were selected for using a forward scatter-area (FSC-A)/ FSC-height (FSC-H) gating. Viable (SYTOX^{TM} blue negative) endothelial cells were FACS-sorted by endothelial marker CD31 positivity and negative selection for the brain microglia and macrophages marker CD45, whereas unsorted endothelial and perivascular cells were obtained from the SYTOX^{TM} blue negative fraction.

Supplementary Figure 2

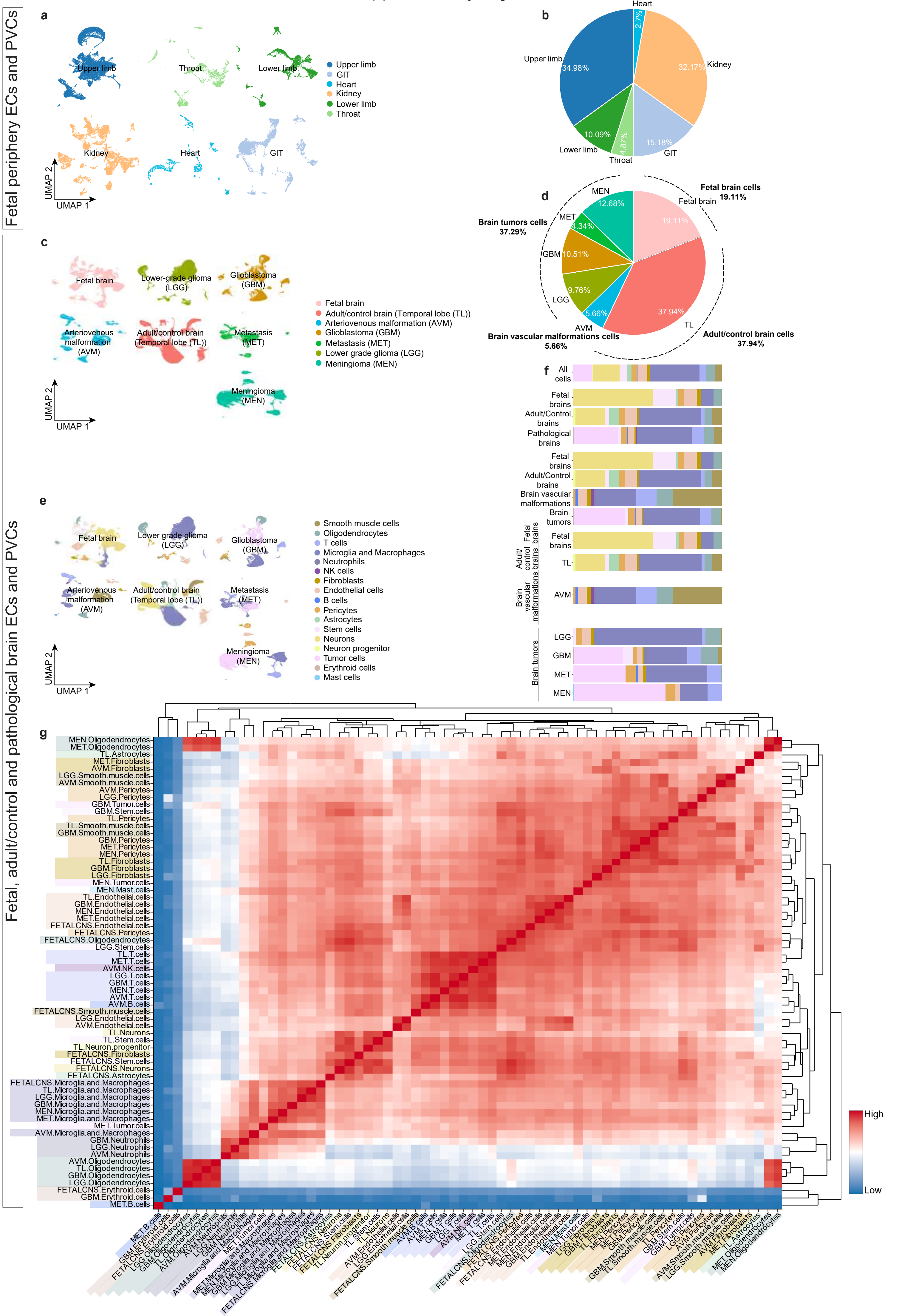

Supplementary Figure 2 | Construction of a molecular single-cell atlas of the human brain vasculature across development, adulthood and disease and of the fetal peripheral vasculature - inter-tissue heterogeneity of unsorted endothelial and perivascular cells

a, Composite UMAP plots of 90,150 human fetal periphery endothelial and perivascular cells, colored by tissue of origin (Upper limb (arm): 31,531 cells from 4 individuals; Kidney: 29,002 from 3 individuals; Throat: 4396 cells from 1 individual; Lower limb (leg): 9097 cells from 1 individual; Heart: 2,440 cells from 1 individual; GI tract: 13,684 cells from 1 individual) (Supplementary Table 3).

b, Pie chart showing relative abundance and percentage of cells from each of the peripheral tissues collected. **c,e,** Composite UMAP plots of human brain endothelial and perivascular cells, colored by tissue of origin (**c**) (Fetal brains: 40,557 cells from 7 individuals; Adult/control brains: 80,515 cells from 6 individuals; Arteriovenous malformations: 12,013 cells from 3 individuals; Lower-grade glioma: 20,712 cells from 4 individuals; Glioblastoma: 22,297 cells from 5 individuals; Metastasis: 9,204 cells from 3 individuals; Meningioma: 26,916 cells from 3 individuals) and by cell type (**e**). **d,** Pie chart showing relative abundance and percentage of cells from each tissue collected. **f,** Relative abundance of cell types from the indicated tissue of origin. Color-code corresponds to (**e**), number of individuals analyzed is as follows: (for All Cells=31, Fetal brains=7, Adult/control brains (TL)=6, All pathological brains=18, Brain vascular malformations=3, Brain tumors=15, AVM=3, LGG=4, GBM=5, MET=3, MEN=3) (Supplementary Table 3). **g,** Endothelial and perivascular cells transcriptome correlation heatmap and hierarchical clustering.

Supplementary Figure 3

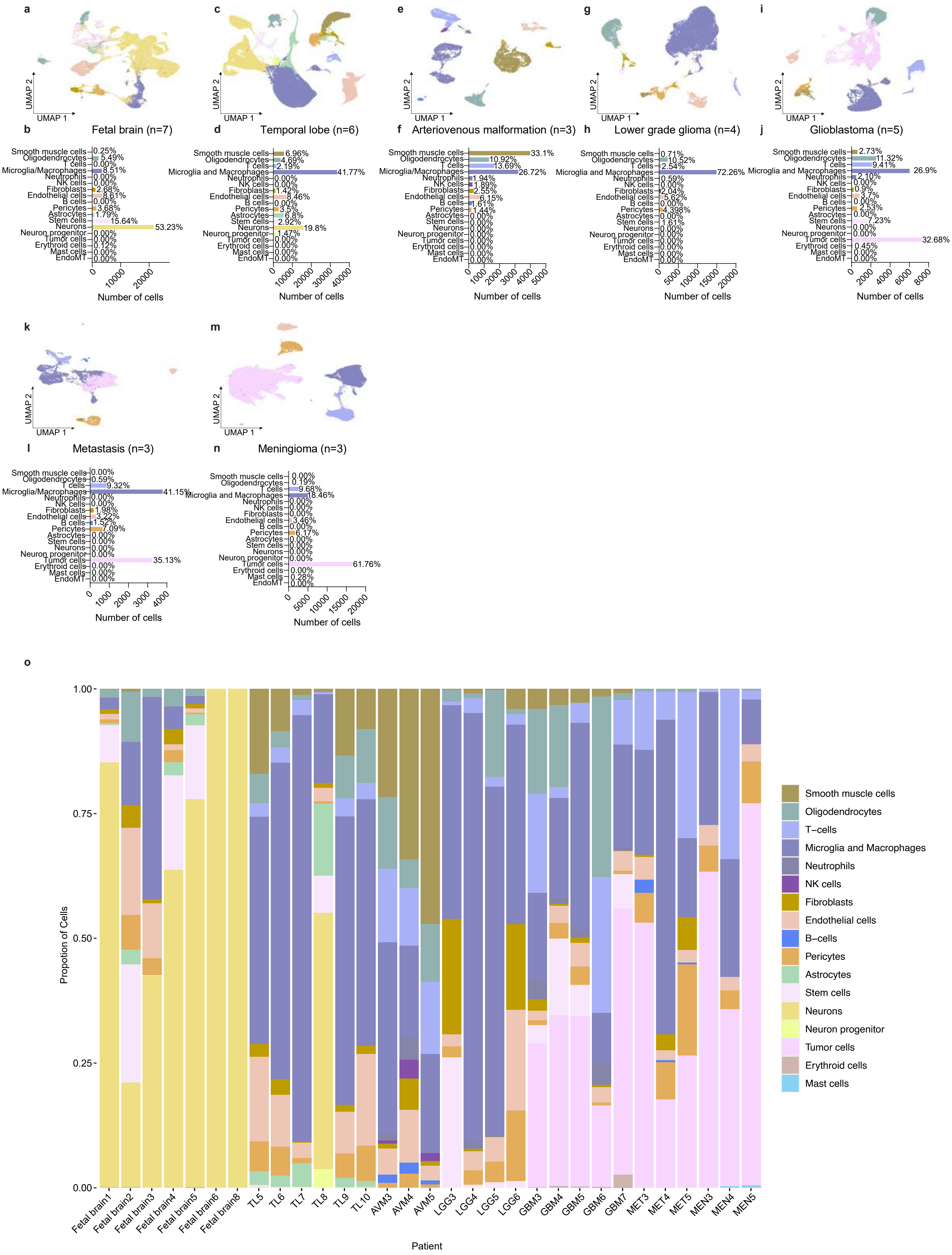

Supplementary Figure 3 | Inter-tissue heterogeneity of unsorted endothelial and perivascular cells in the developing fetal, adult and pathological brain vasculature

a-n, UMAP plots of ECs and PVCs for each tissue of origin indicated, color-coded by cell type. Bar plots showing the number and proportion of cells of each cell type are shown below each corresponding UMAP, (Fetal brains: 40,557 cells from 7 individuals; Adult/control brains: 80,515 cells from 6 individuals; Arteriovenous malformations: 12,013 cells from 3 individuals; Lower-grade glioma: 20,712 cells from 4 individuals; Glioblastoma: 22,297 cells from 5 individuals; Metastasis: 9,204 cells from 3 individuals; Meningioma: 26,916 cells from 3 individuals). **o,** Composite bar graphs showing relative abundance of cell types from the indicated patients and tissues of origin. Color-code corresponds to cell type (see legend).

Supplementary Figure 4

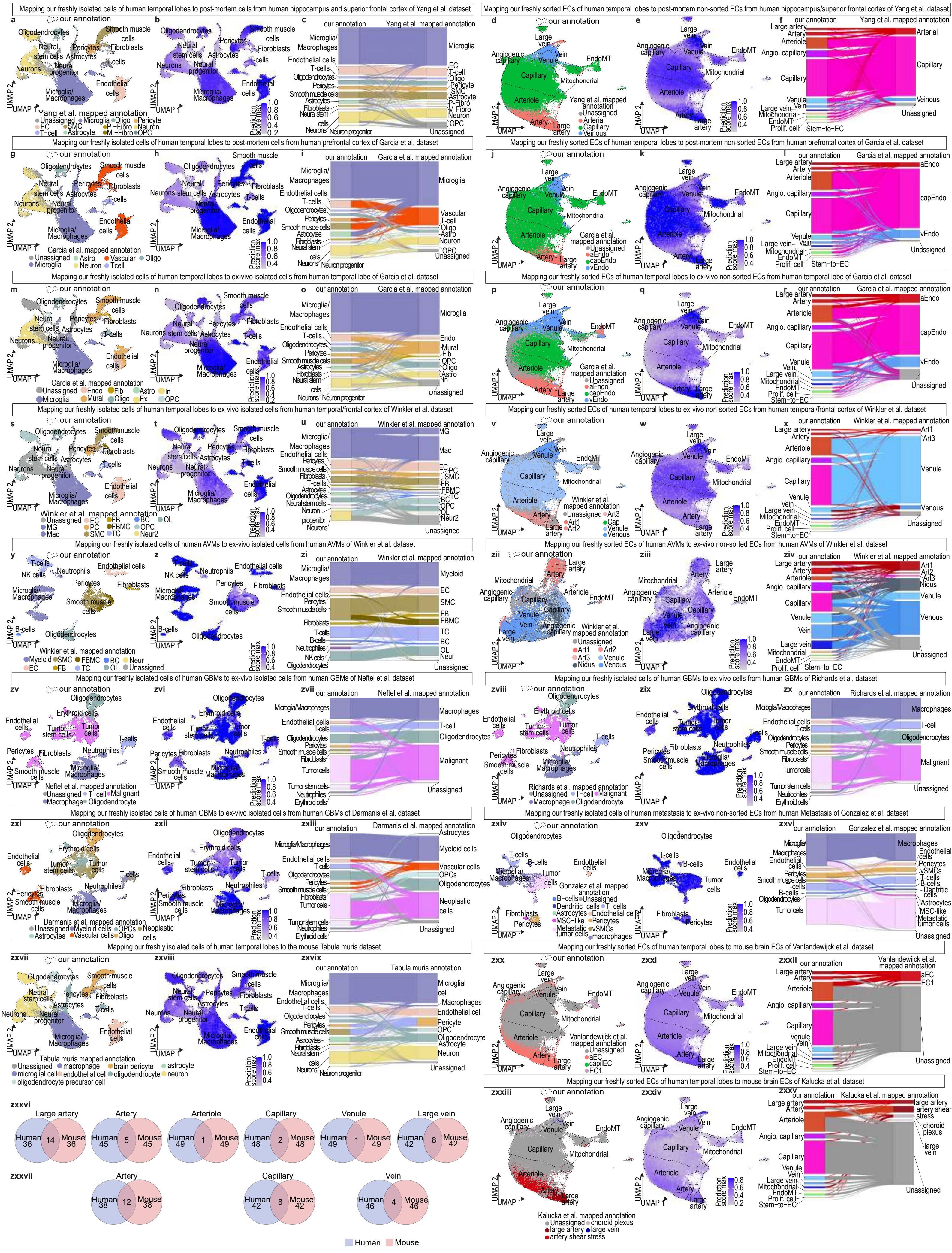

Supplementary Figure 4 | Mapping of our human brain vascular single-cell atlas to publicly available datasets

a-zxxv, Mapping our datasets to the indicated reference dataset indicated. For each of the comparisons, the left panel is a UMAP of the query dataset cells color coded by the predicted annotation based on mapping to the reference dataset, cells with prediction score less than 0.5 are denoted as "unassigned". The dotted lines indicate our annotation of cell clusters based on top cluster marker analysis. The middle panel is a UMAP of the query dataset cells color coded by prediction score based on mapping to the reference dataset indicated. The right panel is a sankey plot showing the predicted annotation (right nodes) of our annotated cells clusters (left nodes) based on mapping to the reference dataset indicated.

zxxvi, Venn diagram showing the overlap between human and mouse AV specification markers (comparing to large artery, artery, arterioles, capillary, venule and large vein of Kalucka et al. dataset). **zxxvii,** Venn diagram showing the overlap between human and mouse AV specification markers (comparing to artery, capillary, vein of Vanlandewijck et al. dataset).

Supplementary Figure 5

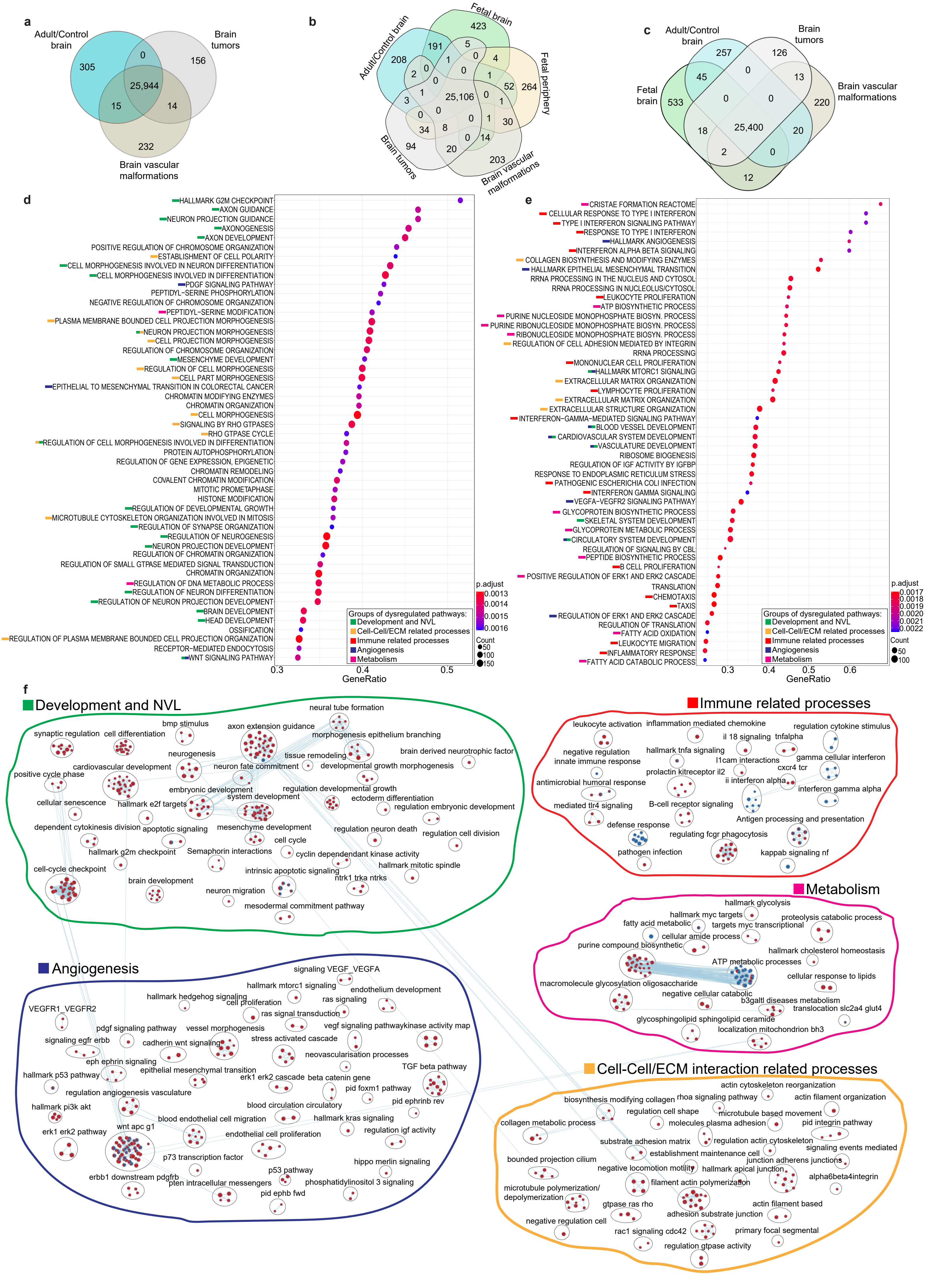

Supplementary Figure 5 | Reactivation of fetal programs in pathological brain vascular ECs

a-c, Venn diagram showing the number of differentially expressed genes between the endothelial cells of the indicated entities, the intersection of all circles indicate the genes that didn’t pass the significance threshold. **d,e,** Over-representation (enrichment) analysis shown as dotplots representing the top 50 pathways enriched in fetal brain ECs as compared to adult/control brains (**d**) and in pathological brain ECs over adult/control brain ECs (**e**). Pathway analysis was performed using gene-set enrichment analysis (GSEA), Kolmogorov-Smirnov test, a permutation-based P-value is computed and corrected for multiple testing to produce a permutation based Benjamini – Hochberg correction false-discovery rate q-value that ranges from 1 (not significant) to 0 (highly significant). Pathways are color-coded for the biological processes indicated. **f,** Enrichment map visualizing some of the commonly enriched pathways from the GSEA pathway analysis, commonly enriched to fetal and pathological brain endothelial cells as compared to adult/control brain endothelial cells. Results include enriched genesets belonging to development and NVL, angiogenesis, cell-cell/extracellular matrix interaction, metabolism, and immune related processes.

Supplementary Figure 6

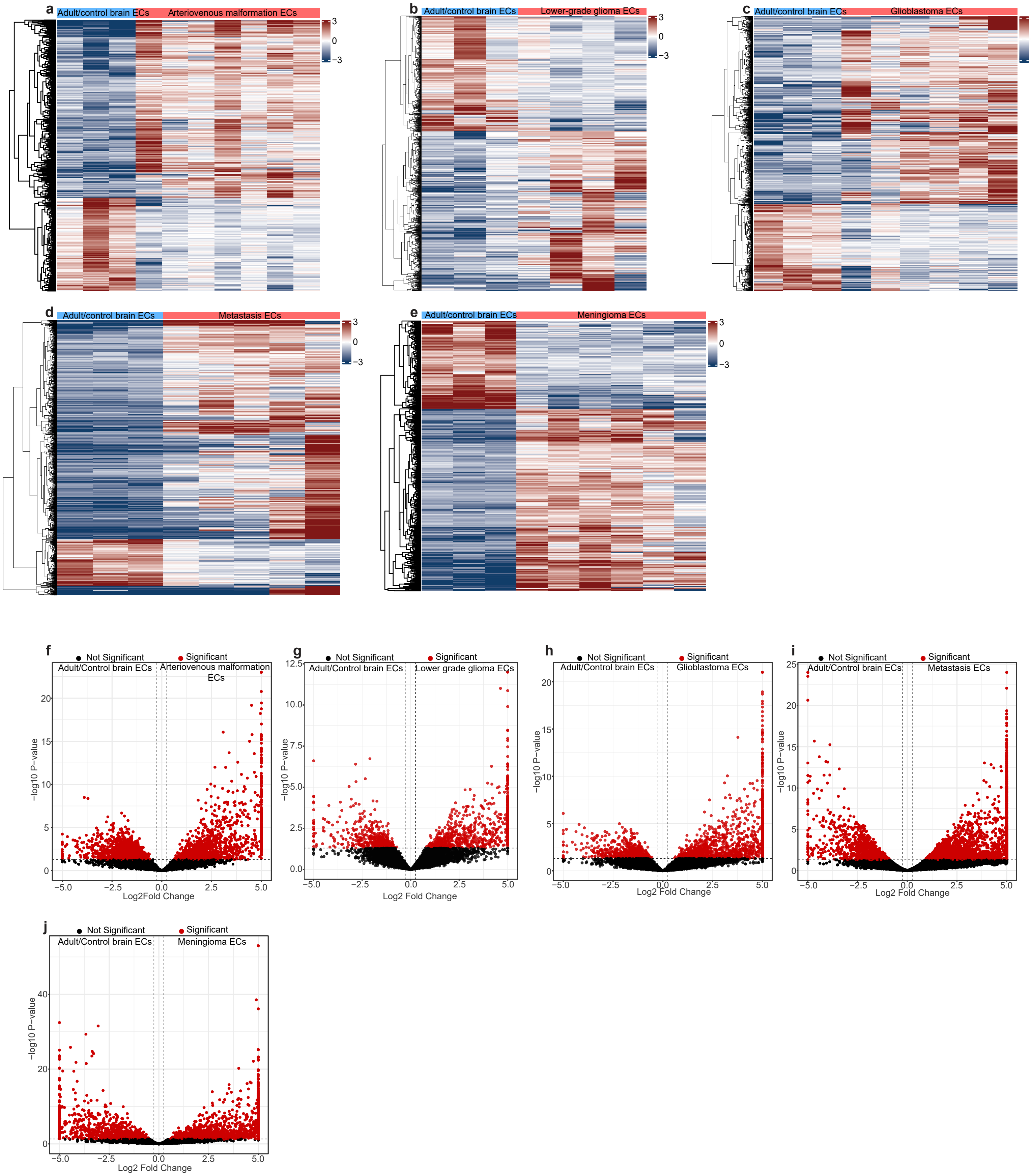

Supplementary Figure 6 | Bulk RNA-seq analysis of $CD31^{+}$/$CD45^{-}$ FACS-sorted human brain vascular ECs of the adult and pathological brain vasculature

Bulk RNA-seq analysis. **a-e,** Heatmaps and hierarchical clustering of all significant genes emanating from differential expression analysis comparing **a,** arteriovenous malformations (number of individuals=7), **b,** lower-grade glioma (LGG) (number of individuals=4), **c,** high-grade glioma/glioblastoma (GBM) (number of individuals=6), **d,** metastasis (MET), **e,** meningioma (MEN) (number of individuals=5), to adult/control brain endothelial cells (number of individuals=3), (Supplementary Table 2). **f-j,** Volcano plots showing the differential expression analysis comparing endothelial cells from adult/control brains (left) and the indicated entity (right). (Wald test, Benjamini Hochberg correction; $p\text{-value} < 0.05$ and $\log_2 FC \geq 0.25$ colored significant in red).

Supplementary Figure 7

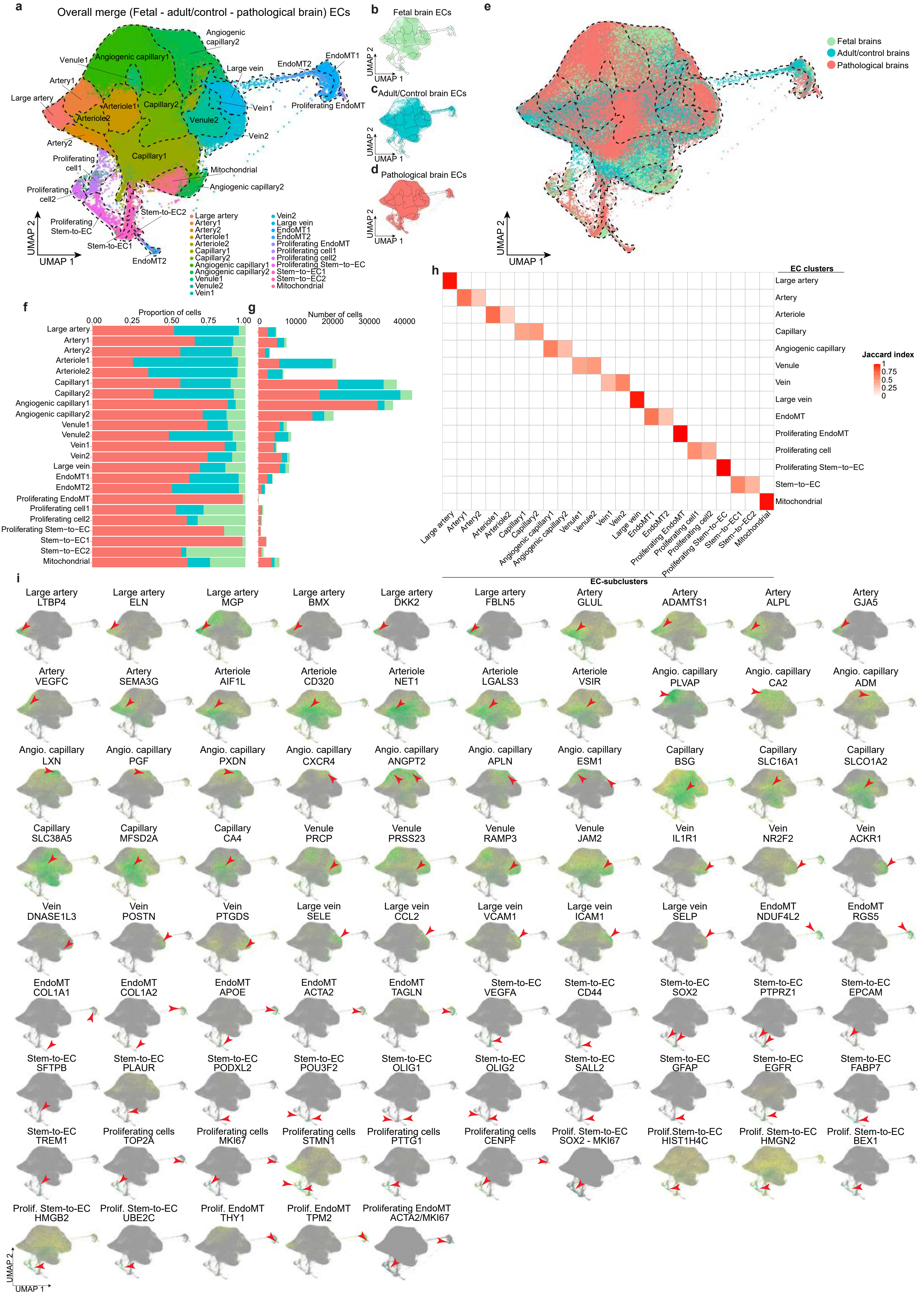

Supplementary Figure 7 | AV-zonation/specification of human brain vascular ECs in the developing fetal, adult and pathological brains vasculature

a,e, UMAP plot of the 243,521 batch corrected ECs, color-coded by EC sub-clusters (**a**), cluster annotation is indicated in the legend and by tissue of origin (**e**) across 5 fetal, 9 adult/control and 29 pathological individuals (Supplementary Table 3). **b-d,** UMAP showed in (**a**) split by tissue of origin: fetal brain (**b**), adult/control brain (**c**) and brain pathologies (**d**). **f,g,** Relative abundance (**f**) and absolute number of (**g**) endothelial cells in the different EC sub-clusters, color-code corresponds to tissue of origin: fetal brain (green), adult/control brain (cyan) and brain pathologies (red). **h,** Heatmap of the pairwise Jaccard distance between the 14 EC clusters (y-axis) and 23 EC subclusters (x-axis).**i,** UMAP plots, color-coded for expression of indicated marker genes (red arrowheads).

Supplementary Figure 8

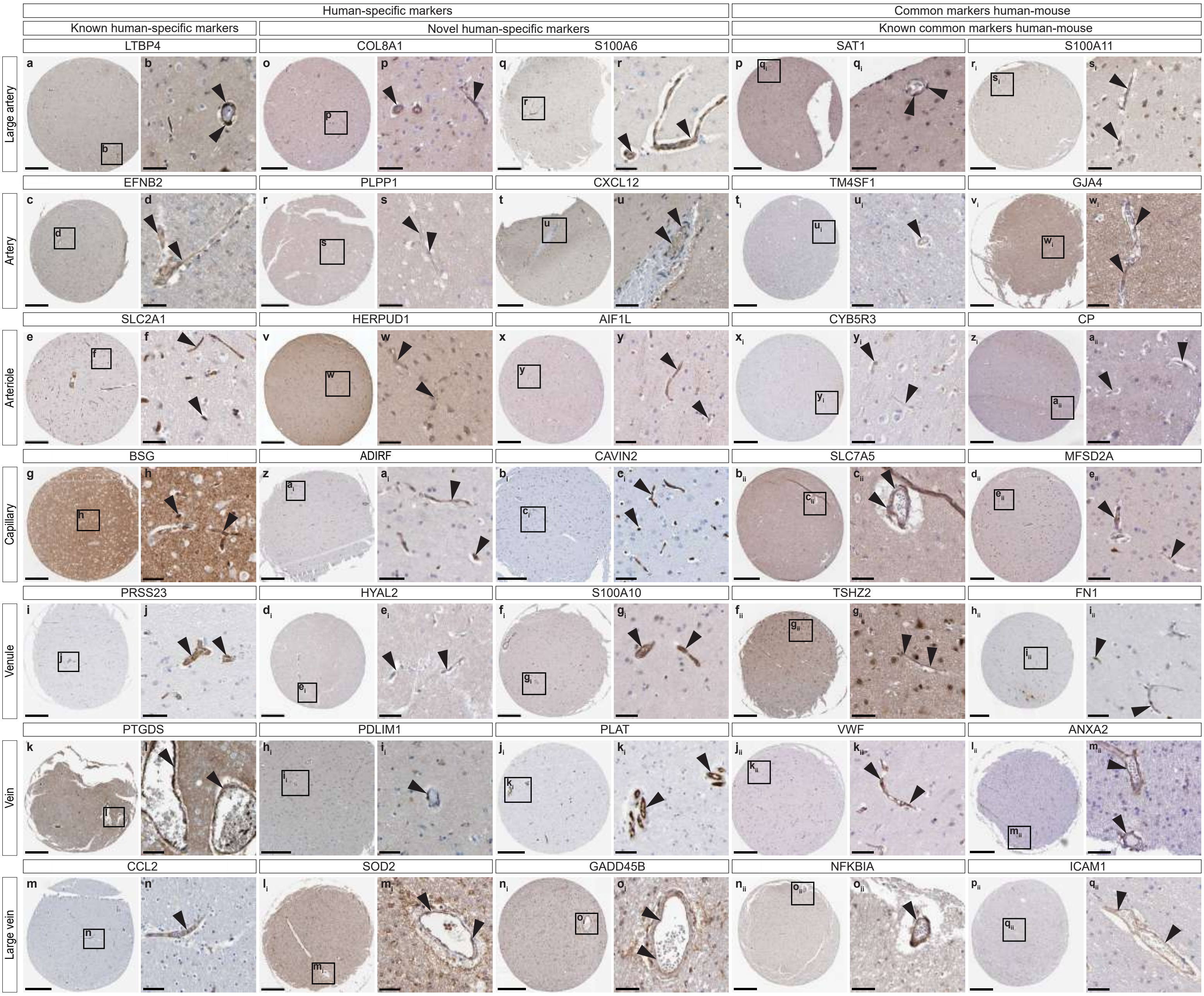

Supplementary Figure 8 | Validation of AV-zonation/specification markers in adult brain tissues of the Human Protein Atlas

a-qii, Immunohistochemistry (IHC) images for the protein expression of the indicated AV specification markers in human cerebral cortex. The images were obtained from the human protein atlas (http://www.proteinatlas.org). **a-n,** Known human-specific AV specification markers. **o-oi,** Novel human-specific AV specification markers, e.g of large arteries (COL8A1 and S100A6), of arteries (PLPP1 and CXCL12), of arterioles (HERPUD1 and AIF1L), of capillaries (ADIRF and CAVIN2), of venules (HYAL2 and S100A10), of veins (PDLIM1 and PLAT), and of large veins (SOD2 and GADD45B). **p-qii,** Known common AV specification markers between human and mouse. The boxed area is magnified on the right; arrowheads indicate vascular structures. Scale bars: 300μm in overviews and 50μm in zooms.

Supplementary Figure 9

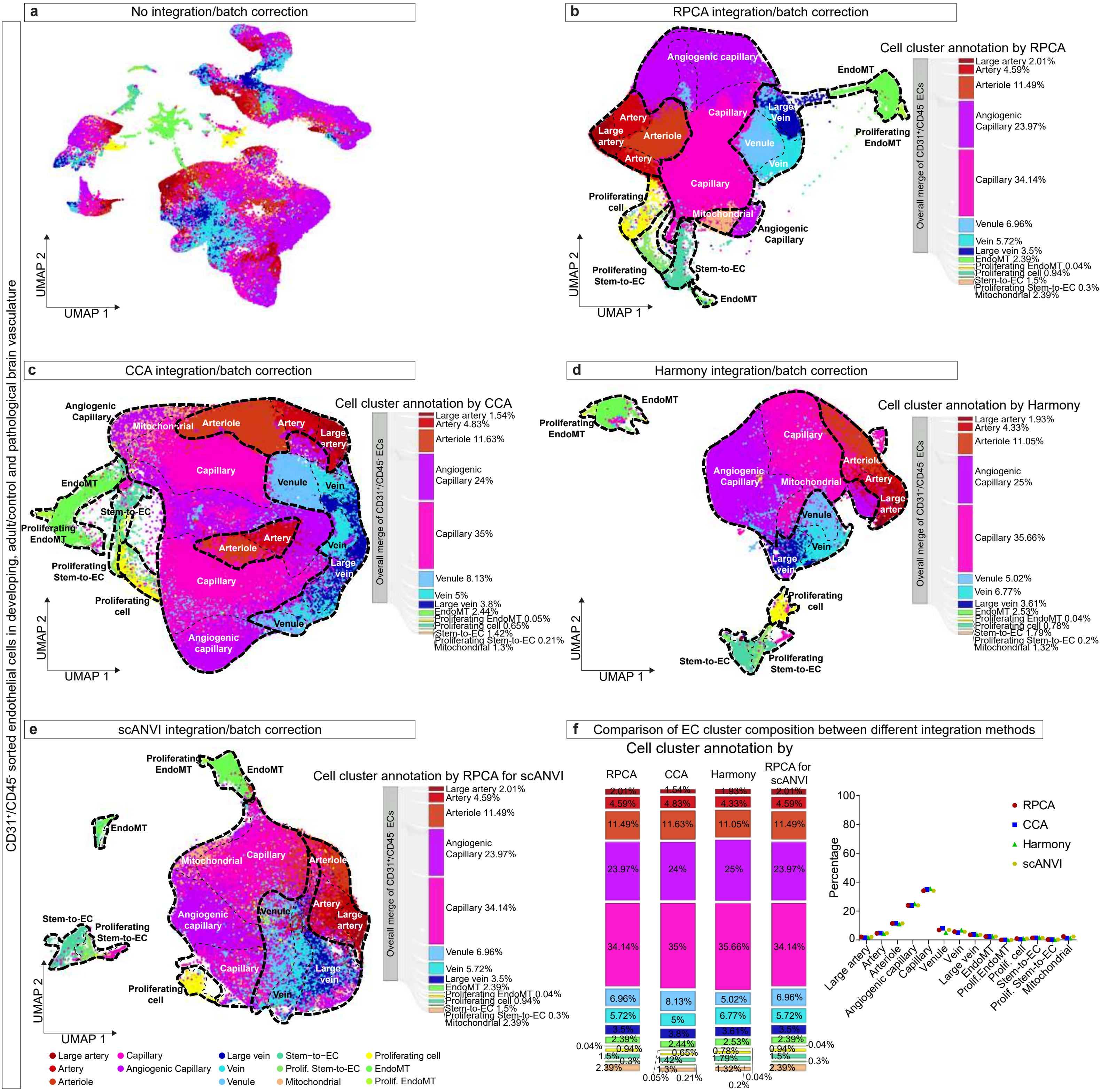

Supplementary Figure 9 | Comparison of methods of integration/batch correction of vascular ECs of the developing fetal, adult and pathological human brain vasculature

a-e, UMAP plots of the 243,521 analyzed $CD31^{+}$/$CD45^{-}$ ECs, color-coded by ECs arteriovenous (AV) specification clusters, with no integration/batch correction (**a**), integrated/batch corrected using: the Seurat-based RPCA (**b**), Seurat-based CCA (**c**), harmony (**d**) and scANVI (**e**) methods. For each of the methods of integration/batch correction performed, a sankey plot shows the resulting relative abundance of EC subtypes (AV specification cluster). **f,** Comparison of the EC cluster composition from the indicated method of integration/batch correction.

Supplementary Figure 10

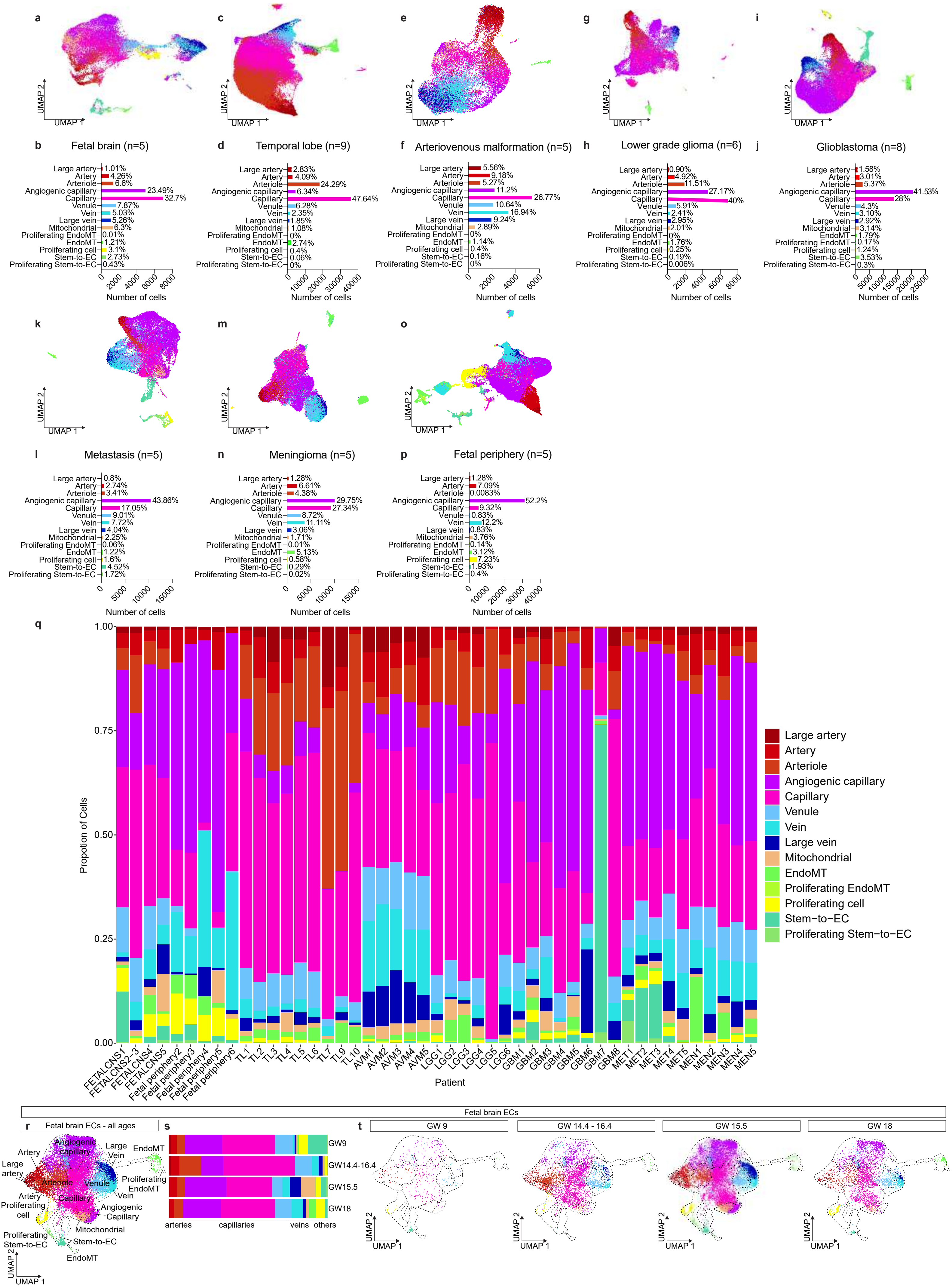

Supplementary Figure 10 | Inter-tissue heterogeneity of sorted vascular endothelial cells in the developing fetal, adult and pathological brains and fetal periphery vasculature

a-p, UMAP plots of ECs for each tissue of origin indicated (Fetal brains: 21,512 ECs from 5 individuals; Adult/control brains: 76,125 ECs from 9 individuals; Arteriovenous malformations: 20,305 ECs from 5 individuals; Lower-grade glioma:17,373 ECs from 6 individuals; Glioblastoma: 49,999 ECs from 8 individuals; Brain metastasis: 23,962 ECs from 5 individuals; Meningioma: 34,245 ECs from 5 individuals; Fetal periphery: 60,495 ECs from 5 individuals), color-coded by ECs arteriovenous (AV) specification. Bar plots showing the number and proportion of each EC subtype are shown below each corresponding UMAP. **q,** Composite bar graphs showing relative abundance of EC subtypes from the indicated patients and tissues of origin. Color-code corresponds to AV cluster (see legend).

r, UMAP plot of fetal brain ECs, color-coded by ECs arteriovenous (AV) specification. **t,** UMAP plot shown in (**r**) split by tissue of gestational ages (GW9 (444 ECs), GW 14.4-16.4 (3762 ECs), GW15.5 (18,314 ECs), GW18 (3601 ECs)). **s,** Relative abundance of EC subtypes (AV specification cluster) from the indicated gestational ages. Color-code corresponds to legend in (**q**).

Supplementary Figure 11

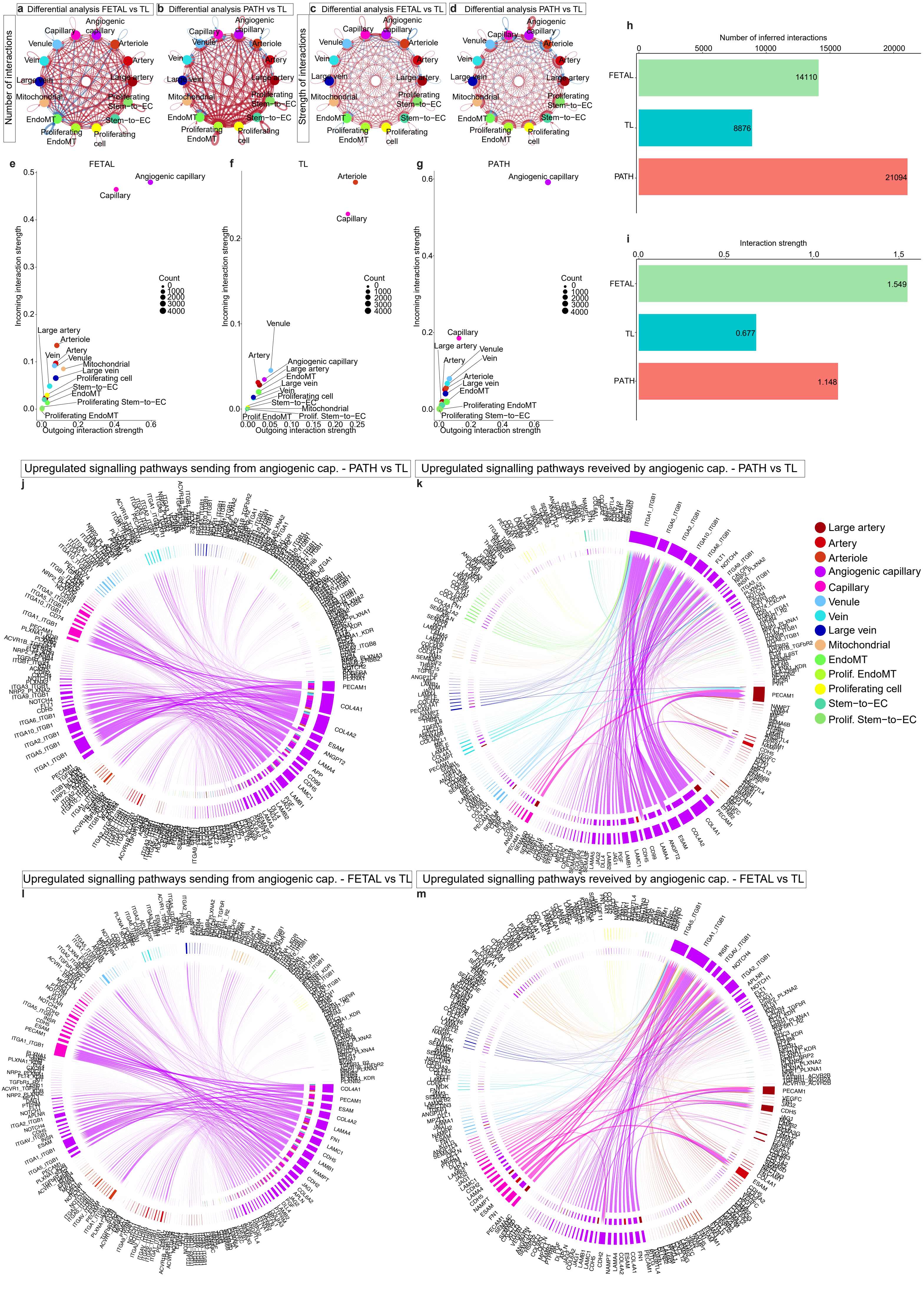

Supplementary Figure 11 | EC-EC interactions inference in the developing, adult and pathological brain vasculature via ligand-receptor analysis

a-d, Circle plots showing the strength of statistically significant signaling interactions between EC subtypes of fetal, adult/control and pathological brains. **a,b,** Differential analysis of the number of interactions for fetal over adult/control brain ECs (**a**) and for pathological over adult/control brain ECs (**b**). **c,d,** Differential analysis of the strength of interactions for fetal over adult/control brain ECs (**c**) and for pathological over adult/control brain ECs (**d**). Red indicating upregulation, while blue indicating downregulation. **e-g,** Scatter plot showing the strength of outgoing (x-axis) and incoming (y-axis) signaling pathways of different EC subtypes from fetal (**e**), adult/control (=TL) (**f**) and pathological brains' EC subtypes (**g**). **h,i,** Barplots showing the number (**h**) and strength (**i**) of interactions in fetal brains (FETAL), adult/control brains (TL) and pathological brains (PATH) endothelial cells. **j,k,** Chord plots showing the ligand-receptor signaling interactions between ECs subtypes upregulated in pathological as compared to adult/control brains; signaling pathways sending from (**j**) and receiving by angiogenic capillaries (**k**). **l,m,** Chord plots showing the ligand-receptor signaling interactions between ECs subtypes upregulated in fetal as compared to adult/control brains; signaling pathways sending from (**l**) and receiving by angiogenic capillaries (**m**). Edge thickness represents edge weights and edge color indicates the sender cell type.

Supplementary Figure 12

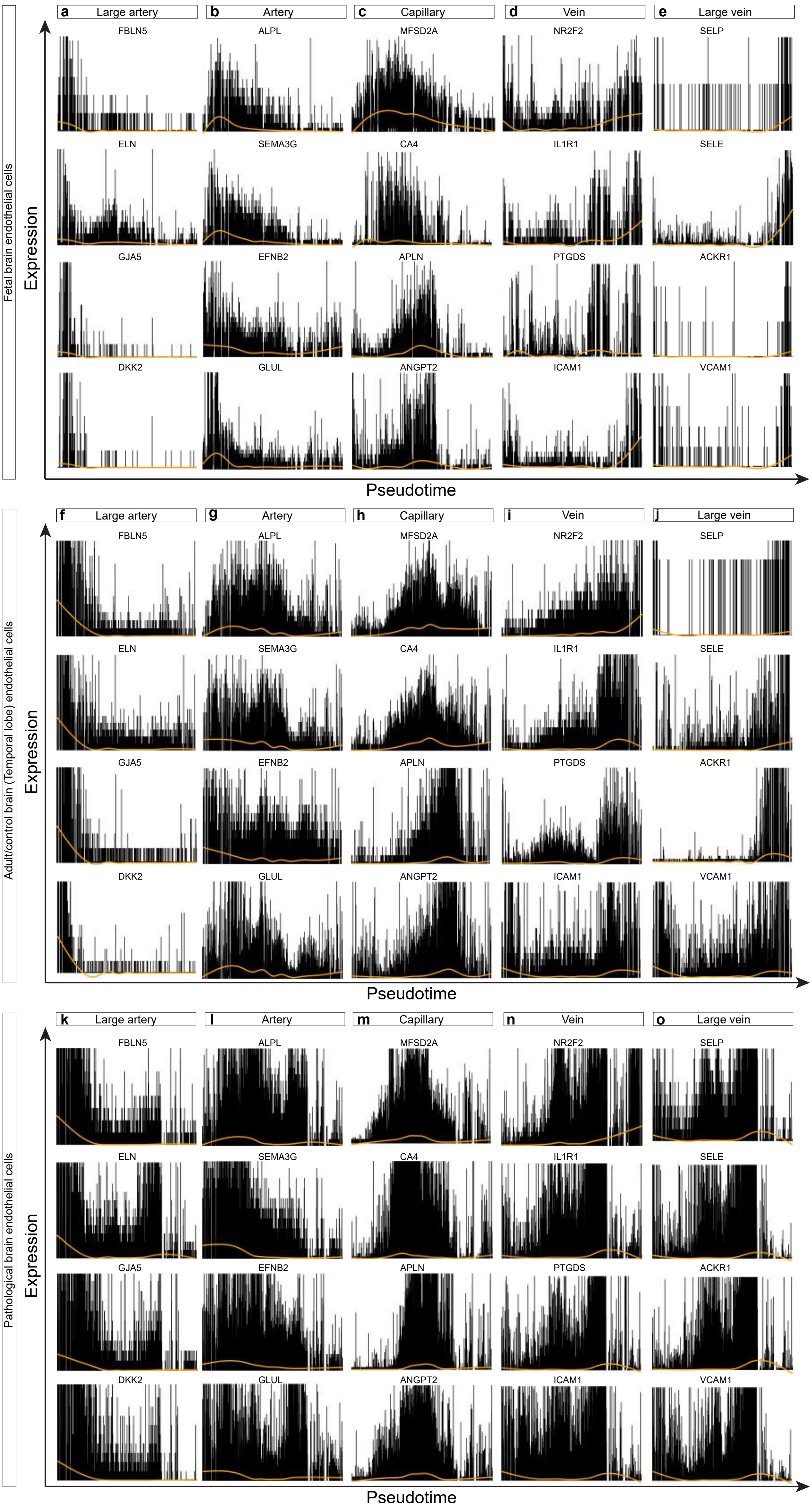

Supplementary Figure 12 | Pseudotime analysis of AV-specification markers along the AV-zonation axis in vascular ECs of the fetal, adult and pathological brain vasculature

Gene expression of the indicated AV specification markers along the pseudotime trajectory in fetal brain (**a-e**), adult/control brain (**f-j**), and pathological brain (**k-o**) ECs. Spline (orange) and density of black lines (counts) correspond with average expression levels.

Supplementary Figure 13

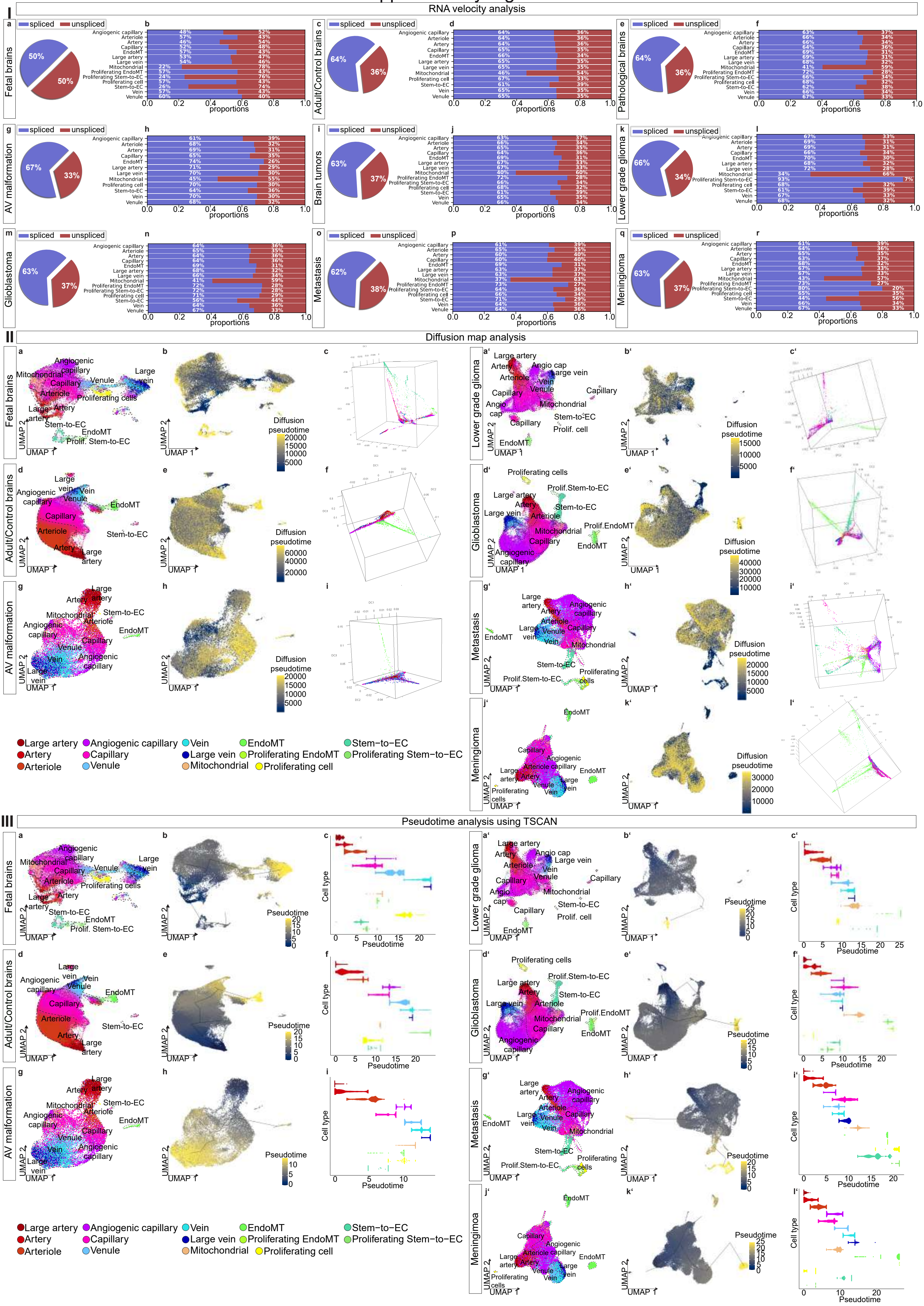

Supplementary Figure 13 | Trajectory analysis of fetal, adult and pathological human brain vascular ECs via RNA velocity, Diffusion map and TSCAN pseudotime along the AV-zonation/specification axis

(I) a-r, Pie charts and composite bar graphs of the of spliced/unspliced transcript proportions in the indicated entities and AV clusters.

(II) a,d,g,a‘,d‘,g‘,j‘, UMAP plots of the indicated entities, colored by AV specification. **b,e,h,b‘,e‘,h‘,k‘,** UMAP plots of the indicated entities, colored by diffusion pseudotime. **c,f,i,c‘,f‘,i‘,l‘,** 3D diffusion map of the indicated entities.

(III) a,d,g,a‘,d‘,g‘,j‘, UMAP plots of the indicated entities, colored by AV specification. **b,e,h,b‘,e‘,h‘,k‘,** UMAP plots of the indicated entities, colored by pseudotime (TSCAN package based analysis). **c,f,i,c‘,f‘,i‘,l‘,** Pseudotime order of ECs color-coded according to AV specification from the indicated entities.

Number of cells and individuals analyzed is as follows: Fetal brains: 21,512 ECs from 5 individuals; Adult/control brains: 76,125 ECs from 9 individuals; Arteriovenous malformations: 20,305 ECs from 5 individuals; Lower-grade glioma:17,373 ECs from 6 individuals; Glioblastoma: 49,999 ECs from 8 individuals; Brain metastasis: 23,962 ECs from 5 individuals; Meningioma: 34,245 ECs from 5 individuals.

Supplementary Figure 14

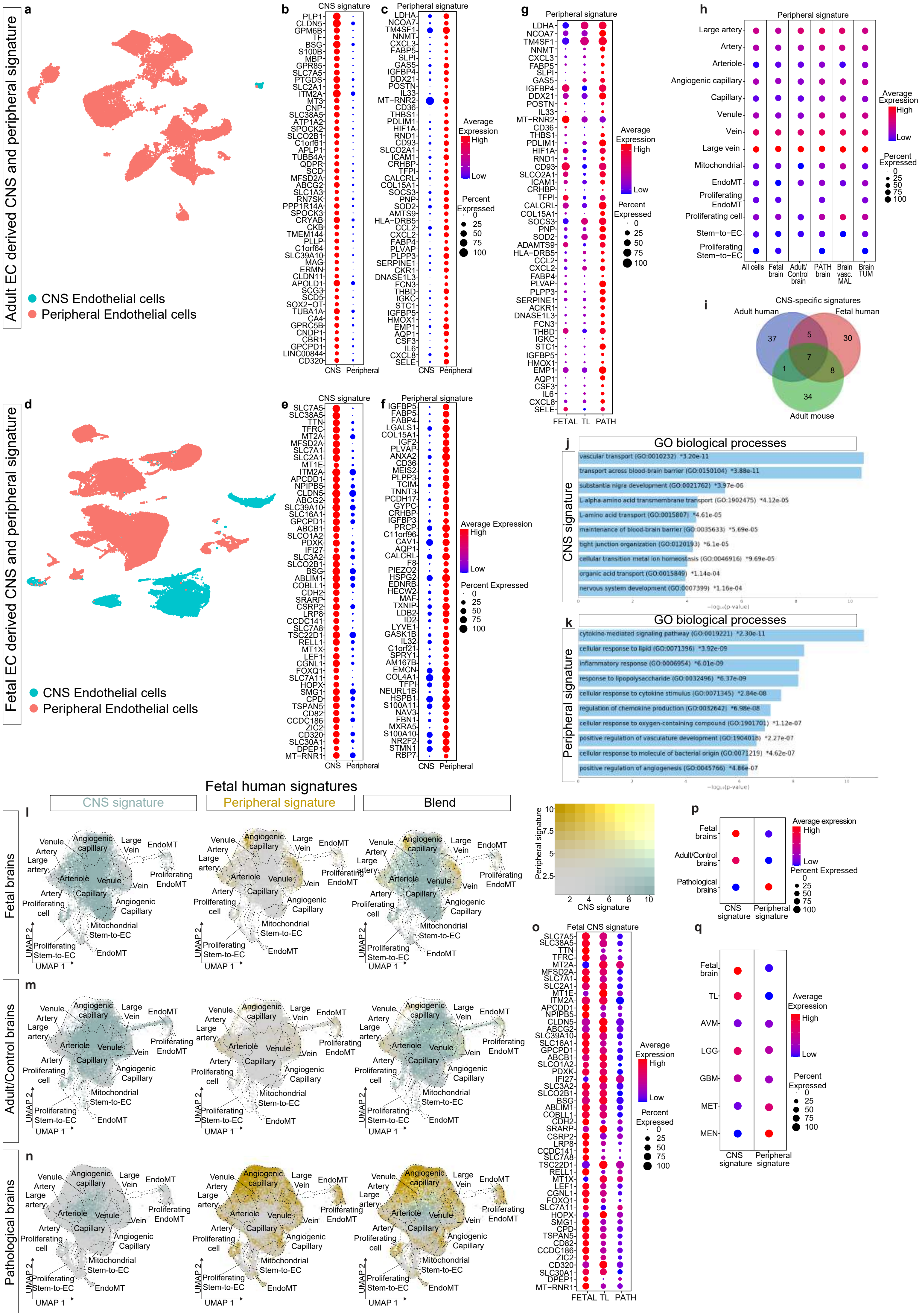

Supplementary Figure 14 | CNS and peripheral signatures in vascular ECs of the developing fetal, adult and pathological brain vasculature

a,d, UMAP plot of adult ECs from Han et al., *Nature* 2020[30] (**a**) and fetal ECs (**d**) colored by CNS and peripheral identity. **b,c,** Dotplot heatmap of the adult endothelial CNS signature (**b**) and peripheral signature (**c**) genes enriched in the respective EC populations. **e,f,** Dotplot heatmap of the fetal endothelial CNS signature (**e**). **g,** Dotplot heatmap of the adult peripheral EC signature genes in fetal, adult/control (temporal lobes) and pathological brain ECs. **h,** Dotplot heatmaps of CNS signature at the level of AV specification for the indicated entities. Color scale: red, high expression; blue, low expression, whereas the dot size represents the percentage expression within the indicated entity. **i,** Venn diagram showing the overlap between the top 50 CNS signature genes obtained from human adult, human fetal and mouse ECs. **j,** Enrichment analysis of human adult CNS endothelial signature showing the top 10 enriched gene ontology biological process (GOBP) genesets, performed on https://maayanlab.cloud/Enrichr/ (Fisher Exact Test). **k,** Enrichment analysis of human adult peripheral endothelial signature. The top 10 enriched GOBP genesets, performed on https://maayanlab.cloud/Enrichr/ (Fisher Exact Test). **l-n**, UMAP plots of the ECs from fetal brains (21,512 ECs from 5 individuals) (**l**), adult/control brains (76,125 ECs from 9 individuals) (**m**), and pathological brains (145,884 ECs from 29 individuals) (**n**). Plots are color-coded for fetal ECs CNS signature (green, left panel), fetal ECs peripheral signature (yellow, middle panel) and a blend of both signatures (right panel). **o,** Dotplot heatmaps of fetal CNS signature genes expression in fetal brain, adult/control brains (temporal lobes) and pathological brain ECs. **p,q,** Dotplot heatmaps of the fetal CNS and peripheral signature expression in fetal brain, adult/control brain, and pathological brain ECs (**p**) and in each individual entity (**q**). Color scale: red, high expression; blue, low expression, whereas the dot size represents the percentage expression within the indicated entity.

Supplementary Figure 15

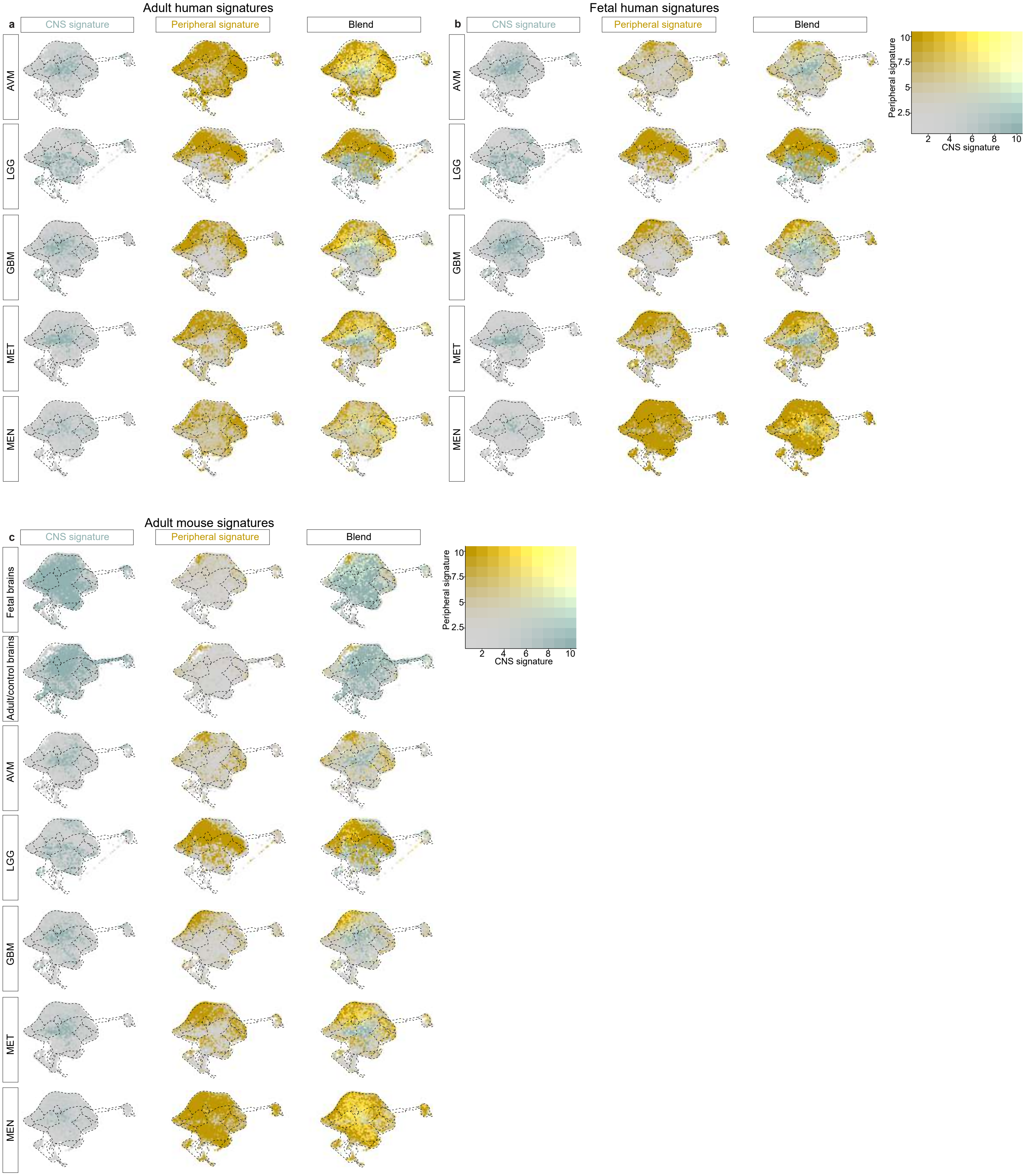

Supplementary Figure 15 | CNS and peripheral signatures in vascular ECs of the developing fetal, adult and pathological brain vasculature, across developmental stages and species

a-c, UMAP plots of ECs from the indicated entities. **a,** Plots are color-coded for human adult EC CNS signature (green, left panel), human adult EC peripheral signature (yellow, middle panel), and blend of both signatures (right panel). **b,** Plots are color-coded for human fetal EC CNS signature (green, left panel), human fetal EC peripheral signature (yellow, middle panel), and blend of both signatures (right panel). **c,** Plots are color-coded for mouse EC CNS signature (green, left panel), mouse EC peripheral signature (yellow, middle panel), and blend of both signatures (right panel). Number of cells and individuals analyzed is as follows: Arteriovenous malformations (AVM): 20,305 ECs from 5 individuals; Lower-grade glioma (LGG):17,373 ECs from 6 individuals; Glioblastoma (GBM): 49,999 ECs from 8 individuals; Brain metastasis (MET): 23,962 ECs from 5 individuals; Meningioma (MEN): 34,245 ECs from 5 individuals.

Supplementary Figure 16

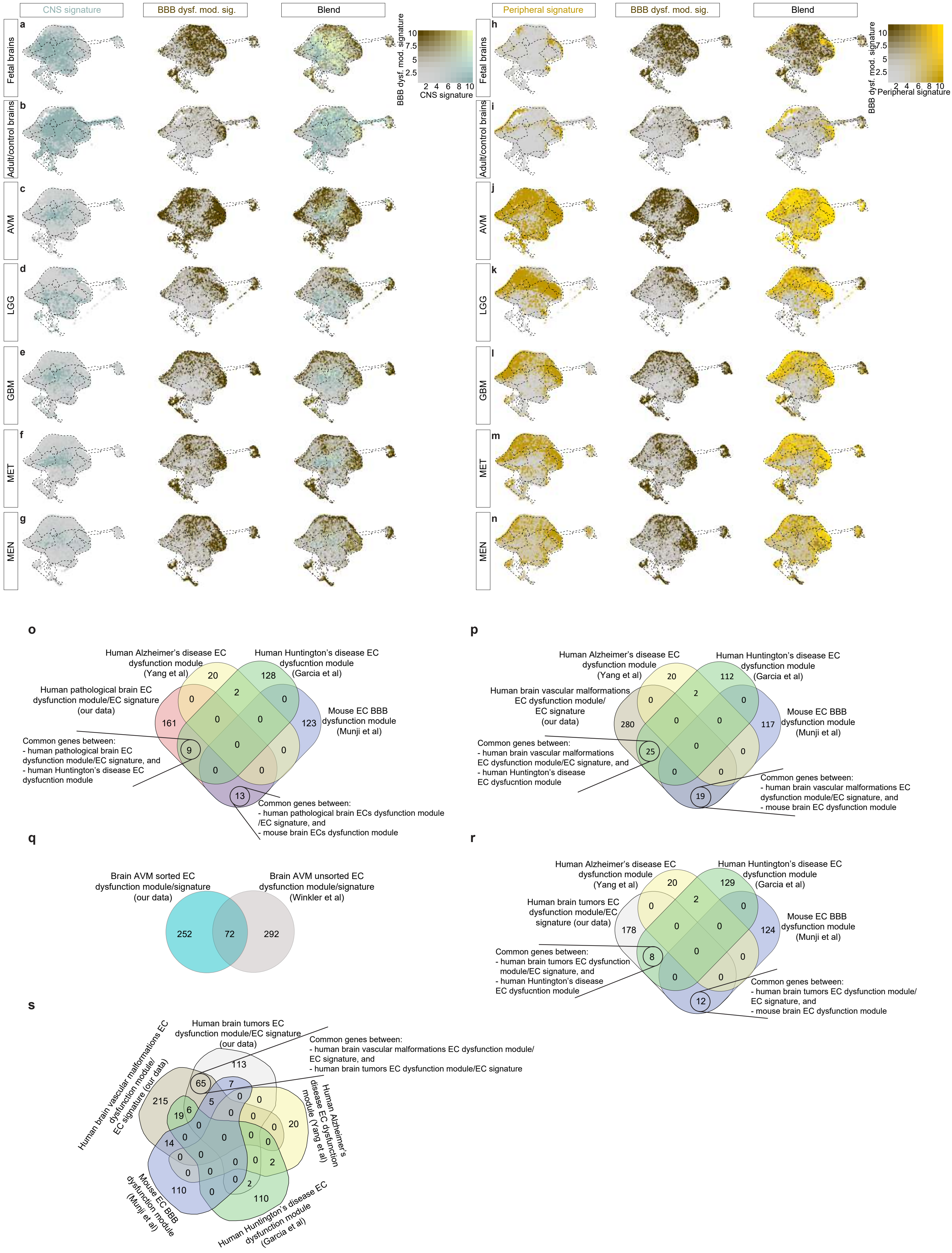

Supplementary Figure 16 | CNS, peripheral signatures and BBB dysfunction modules in vascular ECs of the developing fetal, adult and pathological brain vasculature, and comparison to publicly available datasets

a-n, UMAP plots of ECs from fetal brains (**a,h**), adult/control brains (**b,i**), brain arteriovenous malformations (**c,j**), lower-grade gliomas (**d,k**), high-grade gliomas/glioblastomas (**e,l**), brain metastases (**f,m**), meningiomas (**g,n**). **a-g,** Plots are color-coded for human adult CNS EC signature (green, left panel), mouse brain EC BBB dysfunction module signature (brown, middle panel), and blend of both signatures (right panel). **h-n,** Plots are color-coded for adult human peripheral EC signature (yellow, left panel), mouse brain EC BBB dysfunction module signature (brown, middle panel), and blend of both signatures (right panel). Number of cells and individuals analyzed is as follows: Fetal brains: 21,512 ECs from 5 individuals; Adult/control brains: 76,125 ECs from 9 individuals; Arteriovenous malformations: 20,305 ECs from 5 individuals; Lower-grade glioma:17,373 ECs from 6 individuals; Glioblastoma: 49,999 ECs from 8 individuals; Brain metastasis: 23,962 ECs from 5 individuals; Meningioma: 34,245 ECs from 5 individuals. **o-s,** Venn diagrams showing the overlap of indicated EC signatures' genes.

Supplementary Figure 17

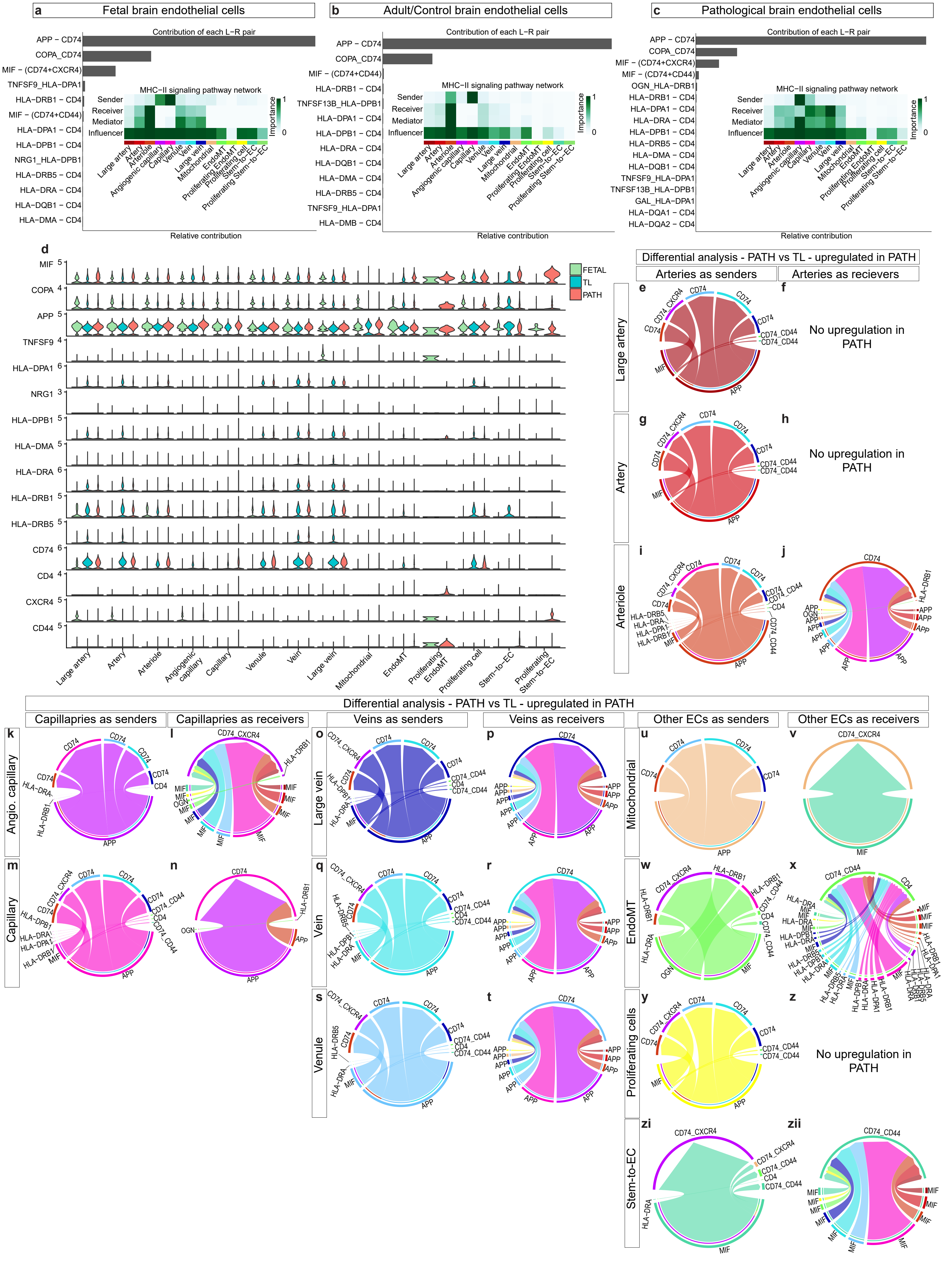

Supplementary Figure 17 | MHC class-II ligand-receptor analysis of EC-EC interactions in the developing, adult and pathological brain vasculature

a-c, Bar plots showing the relative contribution of each ligand-receptor pair to the overall communication network of MHC class II signaling pathway and heatmaps showing the relative importance of each cell type based on the computed network centrality in the fetal (**a**), adult/control (**b**) and pathological (**c**) brain ECs. **d,** Violin plot showing the expression of the main ligand and receptors participating in MHC class II signaling interactions in EC subtypes of fetal, adult control and pathological brains. **e-z_{ii},** Visualization of the differential analysis - pathological brain ECs over adult/control brain ECs - of MHC class II signaling ligand-receptor pairs in the indicated EC subtypes (left panel as sender, and right panel as receiver). **e,g,i,** Chord/circos plots showing the upregulated MHC class II signaling in arteries as source and all EC clusters as targets (**e**, large arteries; **g**, arteries; **i**, arterioles). **f,h,j,** Chord/circos plots showing the upregulated MHC class II signaling in arteries as target and all EC clusters as source (**f**, large arteries for which there was no upregulation; **h**, arteries for which there was no upregulation; **j**, arterioles). **k,m,** Chord/circos plots showing the upregulated MHC class II signaling in capillaries as source and all EC clusters as targets (**k**, angiogenic capillaries; **m**, capillaries). **l,n,** Chord/circos plots showing the upregulated MHC class II signaling in capillaries as target and all EC clusters as source (**l**, angiogenic capillaries; **n**, capillaries). **o,q,s,** Chord/circos plots showing the upregulated MHC class II signaling in veins as source and all EC clusters as targets (**o**, large veins; **q**, veins; **t**, venules). **p,r,t,** Chord/circos plots showing the upregulated MHC class II signaling in veins as target and all EC clusters as source (**p**, large veins; **r**, veins; **t**, venules). **u,** Chord/circos plots showing the upregulated MHC class II signaling in mitochondrial EC subtype as source and all EC clusters as targets. **v,** Chord/circos plots showing the upregulated MHC class II signaling in mitochondrial EC subtype as target and all EC clusters as source. **w,** Chord/circos plots showing the upregulated MHC class II signaling in EndoMT as source and all EC clusters as targets. **x,** Chord/circos plots showing the

upregulated MHC class II signaling in EndoMT as target and all EC clusters as source. **y,** Chord/circos plots showing the upregulated MHC class II signaling in proliferating ECs as source and all EC clusters as targets. **z,** There was no upregulated MHC class II signaling in proliferating ECs as target and all EC clusters as source. **zi,** Chord/circos plots showing the upregulated MHC class II signaling in stem-to-EC as source and all EC clusters as targets. **zii,** Chord/circos plots showing the upregulated MHC class II signaling in stem-to-EC as target and all EC clusters as source.

Supplementary Figure 18

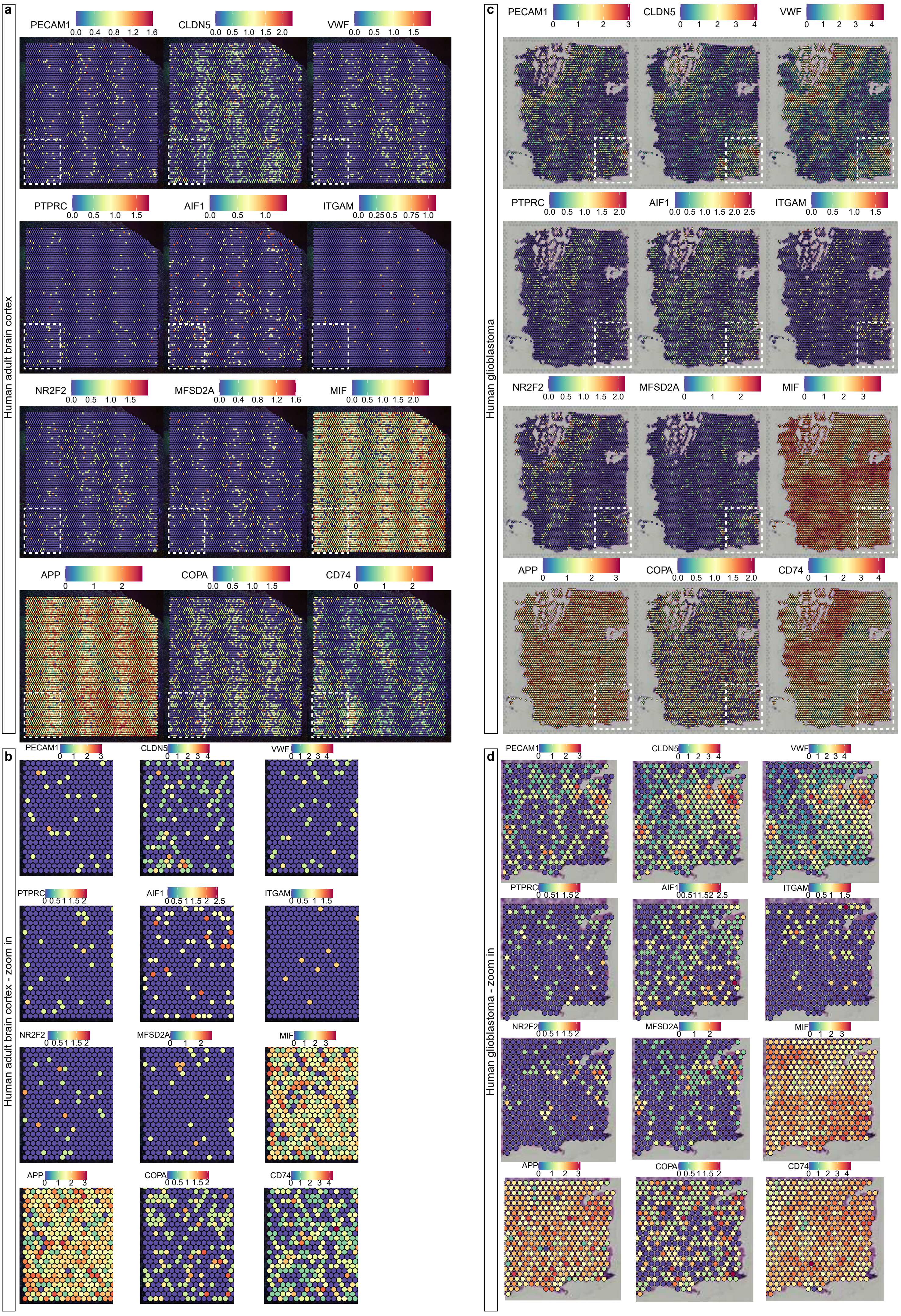

Supplementary Figure 18 | Spatial relationships of MHC class-II receptors in vascular ECs and PVCs in adult brain and in glioblastoma using spatial transcriptomics

a-d, Spatial expression of endothelial markers (PECAM1, CLDN5, VWF), microglia/macrophage markers (PTPRC, AIF1, ITGAM), venous marker (NR2F2), capillary marker (MFSD2A), MHC class II ligands (MIF, APP, COPA) and MHC class II receptor (CD74) in publicly available 10X Visium section of human adult brain cortex (**a,b**) and human glioblastoma (**c,d**). MIF, APP, COPA and CD74 are the predicted interaction partners for EC-EC (venous ECs – capillary ECs) and EC-microglia MHC class II mediated LR interactions. The boxed areas (white dotted box) in (**a**) and (**c**) are zoomed in (**b**) and (**d**) respectively.

Supplementary Figure 19

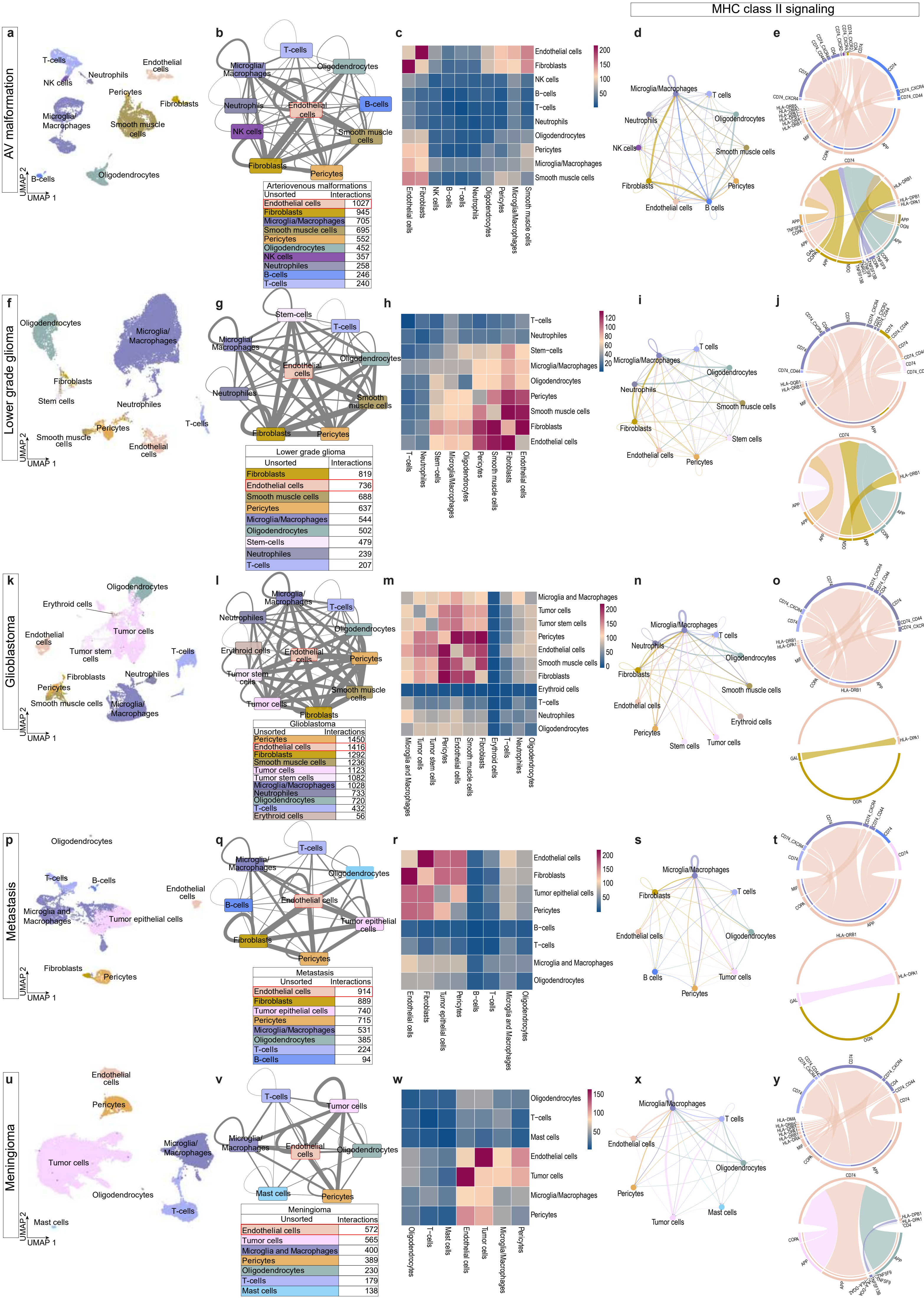

Arteriovenous malformations	
Unsorted	Interactions
Endothelial cells	1027
Fibroblasts	945
Microglia/Macrophages	705
Smooth muscle cells	695
Pericytes	552
Oligodendrocytes	452
NK cells	357
Neutrophiles	258
B-cells	246
T-cells	240

Lower grade glioma	
Unsorted	Interactions
Fibroblasts	819
Endothelial cells	736
Smooth muscle cells	688
Pericytes	637
Microglia/Macrophages	544
Oligodendrocytes	502
Stem-cells	479
Neutrophiles	239
T-cells	207

Glioblastoma	
Unsorted	Interactions
Pericytes	1450
Endothelial cells	1416
Fibroblasts	1292
Smooth muscle cells	1236
Tumor cells	1123
Tumor stem cells	1082
Microglia/Macrophages	1028
Neutrophiles	733
Oligodendrocytes	720
T-cells	432
Erythroid cells	56

Metastasis	
Unsorted	Interactions
Endothelial cells	914
Fibroblasts	889
Tumor epithelial cells	740
Pericytes	715
Microglia/Macrophages	531
Oligodendrocytes	385
T-cells	224
B-cells	94

Meningioma	
Unsorted	Interactions
Endothelial cells	572
Tumor cells	565
Microglia and Macrophages	400
Pericytes	389
Oligodendrocytes	230
T-cells	179
Mast cells	138

Supplementary Figure 19 | EC-PVC interactions inference in the developing, adult and pathological brain neurovascular unit via ligand-receptor analysis

a,f,k,p,u, UMAP plots of endothelial and perivascular cells derived from the indicated entities (Arteriovenous (AV) malformation: 12,013 cells from 3 individuals; Lower-grade glioma: 20,712 cells from 4 individuals; Glioblastoma: 22,297 cells from 5 individuals; Metastasis: 9,204 cells from 3 individuals; Meningioma: 26,916 cells from 3 individuals). **b,g,l,q,v,** Ligand and receptor analysis of the indicated entities done using CellphoneDB. Line thickness indicates the number of interactions between cell types. Tables summarize the number of interactions for each cell type. **c,h,m,r,w,** Heatmaps showing the number of ligand-receptor interactions between the different cells of the indicated entities. **d,i,n,s,x,** Circle plots showing the strength of MHC class II signaling interactions between the different cell types of the indicated entities. **e,j,o,t,y,** Visualization of MHC class II connectomic analysis. Cord/circos plots of MHC class II ligand-receptor interactions with EC as “senders” (upper panel) and as “receivers” (lower panel) in the indicated entities. Edge thickness represents its weights, whereas edge color indicates the “sender” cell type.

Supplementary Figure 20

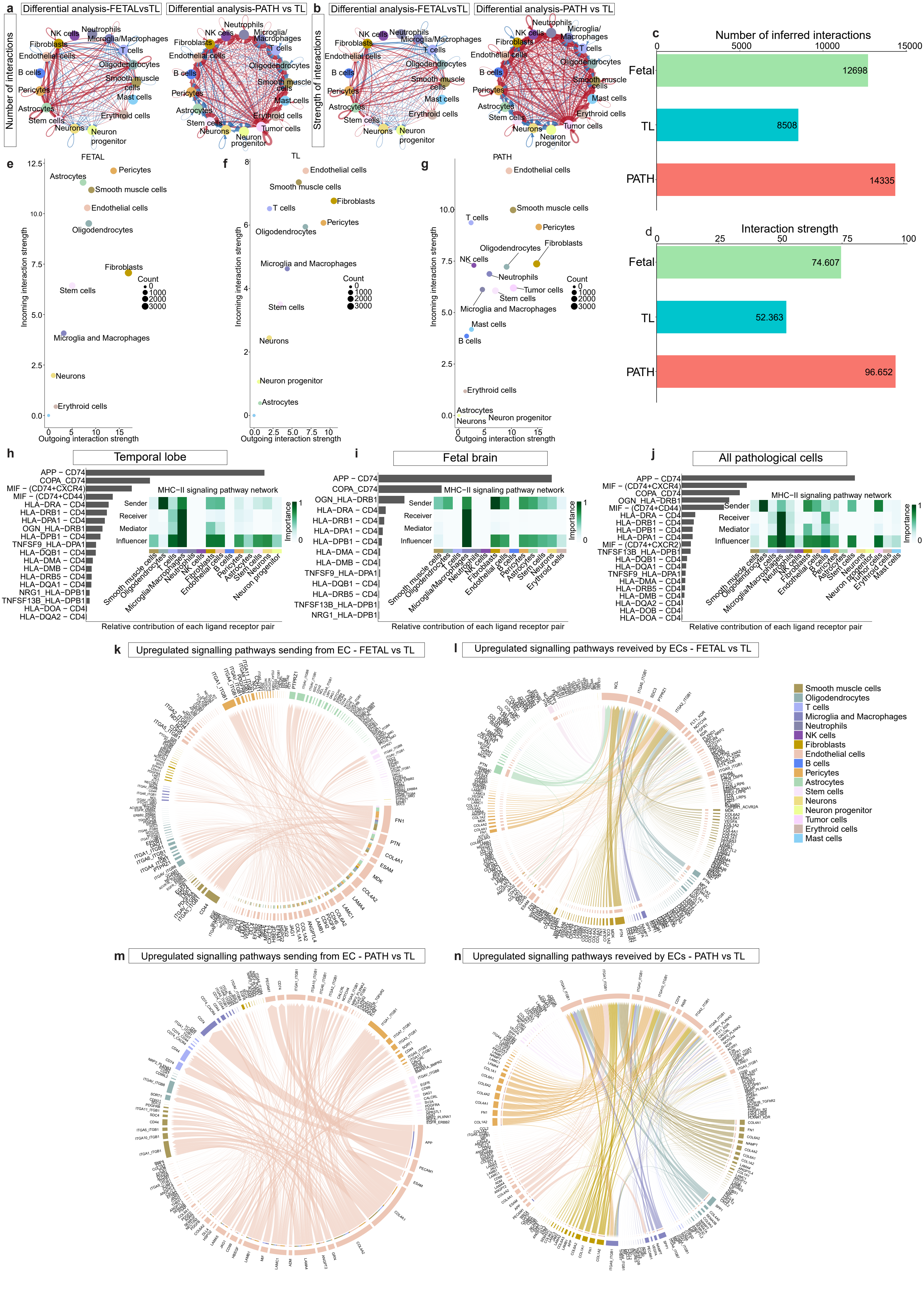

Supplementary Figure 20 | MHC class-II ligand-receptor analysis of EC-PVC interactions in the developing, adult and pathological brain vasculature

a,b, Circle plots showing the strength of statistically significant signaling interactions between cell types of fetal, adult/control and pathological brains. **a,** Differential analysis of the number of interactions for fetal over adult/control brain ECs (left panel) and for pathological over adult/control brain ECs (right panel). **b,** Differential analysis of the strength of interactions for fetal over adult/control brain ECs (left panel) and for pathological over adult/control brain ECs (right panel). Red indicating upregulation, while blue indicating downregulation. **c,d,** Barplots showing the number (**c**) and strength (**d**) of interactions in fetal brains (FETAL), adult/control brains (TL) and pathological brains (PATH) cells.

e-g, Scatter plot showing the strength of outgoing (x-axis) and incoming (y-axis) signaling pathways of different cell types from fetal (**e**), adult/control (=TL) (**f**) and pathological brains (**g**). **h-j**, Bar plots showing the relative contribution of each ligand-receptor pair to the overall communication network of MHC class II signaling pathway, and heatmaps showing the relative importance of each cell type based on the computed network centrality in the indicated tissues. **k,l,** Chord plots showing the ligand-receptor signaling interactions between perivascular cells and ECs upregulated in fetal as compared to adult/control brains; signaling pathways sending from (**k**) and received by endothelial cells (**l**). **m,n,** Chord plots showing the ligand-receptor signaling interactions between perivascular cells and ECs upregulated in pathological as compared to adult/control brains; signaling pathways sending from (**m**) and received by endothelial cells (**n**). Edge thickness represents edge weights and edge color indicates the sender cell type.
