# Supplementary material for: Single-cell atlas of the human brain vasculature across development, adulthood and disease
Source: Nature. 2024 Jul 10;632(8025):603–13. doi: 10.1038/s41586-024-07493-y (PMC11324530; doi:10.1038/s41586-024-07493-y)
Supplement: Supplementary file 2 — Reporting Summary [file 41586_2024_7493_MOESM2_ESM.pdf]

nature portfolio

Corresponding author(s): Thomas Wälchli

Last updated by author(s): Mar 11, 2024

Reporting Summary

Nature Portfolio wishes to improve the reproducibility of the work that we publish. This form provides structure for consistency and transparency in reporting. For further information on Nature Portfolio policies, see our Editorial Policies and the Editorial Policy Checklist.

Statistics

For all statistical analyses, confirm that the following items are present in the figure legend, table legend, main text, or Methods section.

n/a	Confirmed	
☐	☒	The exact sample size (*n*) for each experimental group/condition, given as a discrete number and unit of measurement
☐	☒	A statement on whether measurements were taken from distinct samples or whether the same sample was measured repeatedly
☐	☒	The statistical test(s) used AND whether they are one- or two-sided<br>*Only common tests should be described solely by name; describe more complex techniques in the Methods section.*
☐	☒	A description of all covariates tested
☐	☒	A description of any assumptions or corrections, such as tests of normality and adjustment for multiple comparisons
☒	☐	A full description of the statistical parameters including central tendency (e.g. means) or other basic estimates (e.g. regression coefficient) AND variation (e.g. standard deviation) or associated estimates of uncertainty (e.g. confidence intervals)
☐	☒	For null hypothesis testing, the test statistic (e.g. *F*, *t*, *r*) with confidence intervals, effect sizes, degrees of freedom and *P* value noted<br>*Give P values as exact values whenever suitable.*
☒	☐	For Bayesian analysis, information on the choice of priors and Markov chain Monte Carlo settings
☒	☐	For hierarchical and complex designs, identification of the appropriate level for tests and full reporting of outcomes
☐	☒	Estimates of effect sizes (e.g. Cohen's *d*, Pearson's *r*), indicating how they were calculated

Our web collection on statistics for biologists contains articles on many of the points above.

Software and code

Policy information about availability of computer code

Data collection	FACS Aria III (BD Bioscience), Cellranger-5.0.0, Zeiss Zen 2.3 software
Data analysis	Single-cell RNA-seq libraries (chromium next GEM single cell 3' gene expression libraries) were obtained following the 10x Genomics recommended protocol, using the reagents included in the Chromium Single Cell v3 Reagent Kit. Quality control of cDNA and final libraries was done using 4200 TapeStation System (Agilent) and D5000 ScreenTape & Reagents. Libraries were sequenced on the NextSeq 500 (Illumina) instrument, aiming at 50k reads per cell. The 10x Genomics scRNA-seq data was processed using cellranger-5.0.0 with the Homo sapiens Gencode GRChm38.p13 genome Ensembl release. Based on filtered gene-cell count matrix by CellRanger's default cell calling algorithm, we performed the standard Seurat clustering (version 4.0.0 in R 4.2.2) workflow, as described below; raw expression values were normalized and log transformed (normalization.method = "LogNormalize"). In order to exclude low quality cells and doublets, cells with less than 500 or more than 3000 detected genes were filtered out. scDblFinder (v3.13) was used to validate that doublets were minimal. We also filtered cells with > 25% mitochondrial counts (Supplementary Tables 3,4). For integration/batch correction at the level of the overall merge of sorted endothelial cells of all entities Seurat's reciprocal PCA (RPCA) was applied as described in the "Methods" section. For integration/batch correction of sorted endothelial cells at the level of individual entities separately for every entity Seurat's reciprocal PCA (RPCA) was applied. For integration/batch correction of unsorted cells at the level of individual entities separately for every entity separately, Seurat's reciprocal PCA (RPCA) was applied. The integration/batch correction was not performed at the level of the overall merge of unsorted cells across all entities because we did not have biological questions that would required to do so. The detailed description of the integration/batch correction is present in the "Methods" section. In order to validate the integration results obtained by Seurat reciprocal PCA (RPCA), integration/batch correction was also performed with: 1) Harmony integration/batch correction(https://github.com/immunogenomics/harmony) (REF: https://www.nature.com/articles/s41592-019-0619-0), 2) Seurat's canonical correlation analysis (CCA) integration/batch correction, 3) scANVI (https://github.com/scverse/scvi-tools).<br>For cluster annotation, differential expression analysis for each cluster against all other clusters was computed using wilcoxauc function implemented in the github package presto (v1.0.0) (https://github.com/immunogenomics/presto) (FDR values were calculated using the Benjamini–Hochberg method). The resulting positive markers (that passed the threshold of p value < 0.05, and log2FC > 0.25) were used as

the top cluster markers for annotation.
Heatmap of the pairwise Jaccard distance between the 14 main EC clusters and 44 EC subclusters was computed using scclusteval package.
BIOMEX (version 1.0.0) (https://carmelietlab.sites.vib.be/en/biomex) was using to perform the cluster similarity analysis (Figure 3m-p) as described in sections 33 and 37 of the package manual.
To predict the cell identity of the pathological brain endothelial cell clusters of the adult and fetal datasets as compared to the adult/control brain endothelial cells, the cell identity classification and label transfer was done using the standard Seurat workflow using the temporal lobe endothelial cells. as the reference dataset. Illustration of the results was generated using Seurat (v.4.0.0), Sankey plots were done using networkD3 (v.0.4).
For patient level analysis, DESeq2 package (v1.30.1) was used to perform pseudobulk differential expression analysis, volcano plots were done using EnhancedVolcano (v1.8.0) package and heatmaps were plotted using pheatmap (v1.0.12).
To statistically quantify the compositional changes between fetal, adult/control and pathological brain datasets, we used single-cell differential composition analysis (scCODA) package (version 0.1.7). Moreover, to validate scCODA findings while adjusting for covariates age and sex, we used: 1) tree-aggregated amplicon and single-cell compositional data analysis (tascCODA) (version 0.1.3) (https://github.com/bio-datascience/tascCODA), 2) Dirichlet regression was used via DirichletReg R package version 0.7.0 (https://github.com/maiermarco/DirichletReg), 3) The propeller method was used via speckle R package (0.99.1) (https://github.com/phipsonlab/speckle), 4) Single-Cell Interpretable Tensor Decomposition (scITD) (version 1.0.2) (https://github.com/kharchenkolab/scITD#walkthrough). As described in the methods section.
For bulk RNA-sequencing, the quality of the RNA and final libraries was determined using an Agilent 4200 TapeStation System. The libraries were pooled equimolarly and sequenced in an Illumina NovaSeq sequencer (single-end 100 bp) with a depth of around 20 Mio reads per sample. For mapping and trimming of FASTQ format sequences was performed using Trimmomatic v0.3.3, and sequence quality control was assessed using FastQC. Alignment to the Ensembl Homo_sapiens GRCh38.p10 reference genome (Release_91-2018-02-26) was performed using the STAR aligner. Gene expression values were computed with the function featureCounts from the R package Rsubread.
Sorted endothelial cells bulk RNA-seq deconvolution was performed using BayesPrism (version 2.0) (https://github.com/Danko-Lab/BayesPrism) and EPIC (version 1.1.5) (https://github.com/GfellerLab/EPIC) R packages.
Differential expression was computed using the wilcoxauc function implemented in the github package presto (https://github.com/immunogenomics/presto). FDR values were calculated using the Benjamini–Hochberg method. Pathway analysis was performed using the Gene Set Enrichment Analysis (GSEA) software from the Broad Institute (software.broadinstitute.org/GSEA) (version 4.0.1). A permutation-based P-value is computed and corrected for multiple testing to produce a permutation based Benjamini – Hochberg correction false-discovery rate q-value that ranges from 1 (not significant) to 0 (highly significant). The resulting pathways were ranked using NES and FDR q-value, P-values were reported in the GSEA output reports.
Human_GOBP_AllPathways_no_GO_iea_March_01_2021_symbol.gmt from [http://baderlab.org/GeneSets] was used to identify enriched pathways in GSEA analysis. Highly related pathways were grouped into a themes, labeled by AutoAnnotate (version 1.3) and plotted using Cytoscape (Version 3.7.0) and EnrichmentMap (version 3.3).
Pseudospace/pseudotime trajectory analysis was performed using Monocle 3 (version 1.0.0) (https://github.com/cole-trapnell-lab/monocle3) and Tools for Single Cell Analysis (TSCAN) (1.36.0) (https://github.com/zji90/TSCAN) in fetal, adult/control and pathological brain endothelial cells. Endothelial cells were clustered using the standard Seurat (version 4.0.0) clustering procedure and cluster markers were used to AV annotate those clusters, which were used as an input into Monocle to infer trajectory/lineage/arteriovenous relationships within endothelial cells. SeuratWrappers (v.0.3.0) was used to convert the Seurat objects to cell data set objects, while retaining the Seurat generated UMAP embeddings and cell clustering and then trajectory graph learning and pseudo-time measurement with Monocle3.
To further address trajectory inference, we performed RNA velocity and diffusion map analyses, which both address the pseudotime but not the pseudospace. To compute RNA velocity of endothelial cells of the different entities, Velocyto package (version 0.17.17) (https://velocyto.org/velocyto.py/) was used on the CellRanger output BAM files and the genome annotation (.gtf file) from (https://support.10xgenomics.com/single-cell-gene-expression/software/pipelines/latest/advanced/references). For RNA velocity visualization, the generated loom file containing the spliced and unspliced RNA transcripts served as input into the scVelo package (version 0.2.4) (https://scvelo.readthedocs.io/en/stable/). Trajectory inference was also analyzed using destiny package (version 3.12.0) (https://bioconductor.org/packages/release/bioc/html/destiny.html), the method infers the low-dimensional manifold by estimating the eigenvalues and eigenvectors for the diffusion operator related to the data, In brief, the diffusion map was generated by applying the "DiffusionMap" function on the single cell experiment object.
Cell-cell (ligand receptor) interaction analysis between vascular cell types as well as endothelial and perivascular cells was performed using two published packages: CellPhoneDB (version 3.0.0) and Cellchat (version 1.6.1). First, using CellphoneDB ligand-receptor pairing matrix was constructed as follows; only ligands and receptors expressed in at least 10% of the cells in a particular cluster were considered, cluster labels were then permuted randomly 1,000 times to calculate the mean expression values of ligands and receptors, followed by pairwise comparisons between all cell types. The cut-off of expression was set to more that 0.1 and P-value to less than 0.05. The number of paired cell-cell interactions was based on the sum of the number of ligand-receptor interactions in each of the cell–cell pairs. Finally, Cytoscape was used to visualize the interaction network as a degree sorted circle layout.
Second, using CellChat (v.1.6.1) we followed the developers' suggested workflow, briefly applied the pre-processing functions identifyOverExpressedGenes, identifyOverExpressedInteractions, and projectData with standard parameters set. The CellChatDB including the Secreted Signaling pathways, ECM-receptor as well as Cell-Cell contact were analysed, in addition MHC class-II interactions reported in the CellphoneDB. Moreover, the gene expression data was projected onto experimentally validated protein-protein interaction. the standard package functions as computeCommunProb, computeCommunProbPathway, and aggregateNet were used with default parameters. Finally, to determine the ligand-receptor contributions and senders/receivers' roles in the network the functions netAnalysis_contribution and netAnalysis_signalingRole was applied on the netP data slot respectively.
We further compared cell–cell communication patterns by computing the Euclidean distance between ligand-receptor pairs of the shared signaling pathways (a measure of the difference between the signaling networks of datasets, see methods e.g. larger Euclidean distance implying larger difference of the communication networks between two datasets in terms of either functional or structure similarity, termed network architecture)57. We compared the information flow for each signaling pathway between, which is defined by the sum of communication probability among all pairs of cell groups for a given signaling pathway in the inferred network.

Software used include: R 4.2.2, Python 3.7, FV10-ASW 4.2 Viewer, Zeiss Zen 2.3 software, ImageJ 1.53e.
All data is accessible via the GEO accession number GSE186771 (will be made public as soon as upload is finished).
An interactive website is available at: Waelchli-lab-human-brain-vasculature-atlas.hest.ethz.ch
Our analyses are standard workflows that have been carried out using freely available software packages (detailed in the "Methods" section and also above), and we have now deposited the source code from the respective freely available software packages (indicated above and in the "Methods" section) that we used in:
(https://github.com/Waelchli-lab/Single-cell-atlas-of-the-human-brain-vasculature-accross-development-adulthood-and-disease) to improve

reproducibility our results.

For manuscripts utilizing custom algorithms or software that are central to the research but not yet described in published literature, software must be made available to editors and reviewers. We strongly encourage code deposition in a community repository (e.g. GitHub). See the Nature Portfolio guidelines for submitting code & software for further information.

Data

Policy information about availability of data

All manuscripts must include a data availability statement. This statement should provide the following information, where applicable:

- Accession codes, unique identifiers, or web links for publicly available datasets
- A description of any restrictions on data availability
- For clinical datasets or third party data, please ensure that the statement adheres to our policy

All data is accessible via the GEO accession number GSE256493 (access token: wzermaewdzernab). We have deposited our research data via the Nature Portfolio - figshare partnering on: https://doi.org/10.6084/m9.figshare.25151738

Field-specific reporting

Please select the one below that is the best fit for your research. If you are not sure, read the appropriate sections before making your selection.

☒ Life sciences ☐ Behavioural & social sciences ☐ Ecological, evolutionary & environmental sciences

For a reference copy of the document with all sections, see nature.com/documents/nr-reporting-summary-flat.pdf

Life sciences study design

All studies must disclose on these points even when the disclosure is negative.

Sample size	We performed single cell RNAseq and bulk RNA-seq analysis on the number of fetuses/patients indicated in Supplementary Tables 1,2,3 and Extended data figure 1. We performed single-cell RNA sequencing of 606,380 freshly isolated endothelial, perivascular and other tissue-derived cells from 117 samples, from 68 human fetuses and adult patients to construct a molecular atlas of the developing fetal, adult control and diseased human brain vasculature.
Data exclusions	No data were excluded from the analysis. For the final count matrix, we excluded cells based on pre-established criteria for single-cells: we excluded low quality cells (i.e. - cells with low number of detected genes and high mitochondria content). In order to exclude low quality cells and doublets, cells with less than 500 or more than 3000 detected genes were filtered out. scDblFinder (v3.13) was used to validate that doublets were minimal. We also filtered cells with > 25% mitochondrial counts (Supplementary Tables 3,4).
Replication	We performed single cell RNAseq on the number of fetuses/patients indicated in Supplementary tables 1,2,3 and Extended data figure 1. The number of the different biological replicates are stated in the Supplementary Tables 1,2,3, and those attempts were fresh isolations and sequencing that are successful at the first and only attempt of the experiment. We only processed freshly operated and isolated samples.
Randomization	Different single cells were randomly captured before analysis. Human samples were not randomized due to practical constraints.
Blinding	We are blinded to analyzed cell types before single cell analyses.

Reporting for specific materials, systems and methods

We require information from authors about some types of materials, experimental systems and methods used in many studies. Here, indicate whether each material, system or method listed is relevant to your study. If you are not sure if a list item applies to your research, read the appropriate section before selecting a response.

Materials & experimental systems

n/a	Involved in the study	
☐	☒	Antibodies
☒	☐	Eukaryotic cell lines
☒	☐	Palaeontology and archaeology
☒	☐	Animals and other organisms
☐	☒	Human research participants
☐	☒	Clinical data
☒	☐	Dual use research of concern

Methods

n/a	Involved in the study	
☒	☐	ChIP-seq
☐	☒	Flow cytometry
☒	☐	MRI-based neuroimaging

Antibodies

Antibodies used

ZO1 Mouse Invitrogen 33 9100
Occludin Mouse Invitrogen 33 1500
GLUT1 Mouse Abcam ab40084
CNS-specificity markers
SLC38A5 Rabbit Abcam ab72717
SPOCK3 Rabbit ThermoFischer PA531369
PPP1R14A Rabbit AVIVA Systems Biology OAAF01271
BSG (CD147) Mouse Abcam ab666
CD320 Rabbit Proteintech 10343-1-AP
GPCPD1 Rabbit ThermoFischer PA5-65346
CD31 Guinea pig Synaptic Systems 351004
CD326(EPCAM) Mouse ThermoFisher 14-9326-82
SFTPB Rabbit Invitrogen PA5 42000
SOX2 Rabbit Abcam ab97959
PTPRZ1 Rabbit ThermoFisher PA5-53280
HLA DRB5 Rabbit Invitrogen PA5 60260
HLA DRA Rabbit Invitrogen PA5 27553
HLA DPA1 Rabbit Invitrogen PA5 28037
HLA DPA1 Rabbit Sigma HPA017967
CD74 Rabbit Sigma HPA010592
PLVAP Rabbit Sigma HPA002279
Alexa Fluor Goat anti-mouse 488 Invitrogen A28175
Alexa Fluor Goat anti-rabbit 568 Invitrogen A-11011
Alexa Fluor Donkey anti-mouse 488 Invitrogen A-21202
Alexa Fluor Donkey anti-rat 488 Invitrogen A-21208
Alexa Fluor Donkey anti-guinea pig 488 Jackson Immuno Research 706-545-148
Northern Light Donkey anti-sheep 493 R&D NL012
Northern Light Donkey anti-mouse 493 R&D NL009
Alexa Fluor Donkey anti-mouse 555 Invitrogen A-31570
Alexa Fluor Donkey anti-rabbit 555 Invitrogen A-31572
Northern Light Donkey anti-mouse 557 R&D NL007
Northern Light Donkey anti-rabbit 557 R&D NL004
Northern Light Donkey anti-mouse 637 R&D NL008
Northern Light Donkey anti-rabbit 637 R&D NL005
Northern Light Donkey anti-goat 637 R&D NL002
Alexa Fluor Donkey anti-rabbit 647 Invitrogen A-31573
CD68 KP1 Thermo Fisher 14-0688-82 2265228 https://www.thermofisher.com/antibody/product/CD68-Antibody-clone-KP1-Monoclonal/14-0688-82
CD31 EPR3094 abcam ab207090 GR3229164-11 https://www.abcam.com/cd31-antibody-epr3094-bsa-and-azide-free-ab207090.html
CD11b EPR1344 abcam ab209970 GR3352581-4 https://www.abcam.com/cd11b-antibody-epr1344-bsa-and-azide-free-ab209970.html
IMC Cell Segmentation Kit Protein 1 Protein1-unknown Fluidigm TIS-00001 1742007
IMC Cell Segmentation Kit Protein 2 Protein2-unknown Fluidigm TIS-00001 1742008
IMC Cell Segmentation Kit Protein 3 Protein3-unknown Fluidigm TIS-00001 1882005
CD8a C8/144B Thermo Fisher 14-0085-82 2247491 https://www.thermofisher.com/antibody/product/CD8a-Antibody-clone-C8-144B-Monoclonal/14-0085-82
HLA-DR TAL 1B5 abcam ab176408 GR3384096-1 https://www.abcam.com/hla-dr-antibody-tal-1b5-bsa-and-azide-free-ab176408.html
CD4 EPR6855 abcam ab181724 GR3352909-4 https://www.abcam.com/cd4-antibody-epr6855-bsa-and-azide-free-ab181724.html
SMA (ACTA2) 1A4 Thermo Fisher 14-9760-82 2288516 https://www.thermofisher.com/antibody/product/53-9760-82.html?ef_id=CjwKCAjwquWVBhBrEiwAt1Kmwg0mNovA4BDEYQrLlZsnBTvV6n6aMXnizAycM3_lK84aN4iQFe-8QRoClcQQAvD_BwE:G:s&s_kwcid=AL!3652!3!459736943987!!!g!!&cid=bid_pca_aup_r01_co_cp1359_pjt0000_bid00000_0se_gaw_dy_pur_con&gclid=CjwKCAjwquWVBhBrEiwAt1Kmwg0mNovA4BDEYQrLlZsnBTvV6n6aMXnizAycM3_lK84aN4iQFe-8QRoClcQQAvD_BwE
pan Cytokeratin C11 Thermo Fisher MA1-12594 XB3490763 https://www.thermofisher.com/antibody/product/Cytokeratin-Pan-Antibody-clone-C11-Monoclonal/MA1-12594
pan Cytokeratin AE1 Sigma Aldrich MAB1612 3460341 https://www.sigmaaldrich.com/CA/en/product/mm/mab1612
Keratin Epithelial AE3 Sigma Aldrich MAB1611 3382323 https://www.sigmaaldrich.com/CA/en/product/mm/mab1611
E-Cadherin / P-Cadherin 36/E-Cadherin BD Biosciences 610182 2038668 https://www.bdbiosciences.com/content/bdb/paths/generate-tds-document.cn.610182.pdf
BSG (CD147/Basigin) MEM-M6/1 abcam ab666 GR3344079-2 https://www.abcam.com/cd147-antibody-mem-m61-ab666.html
CLDN5 EPR7583 abcam ab236066 GR3422726-1 https://www.abcam.com/claudin-5-antibody-epr7583-bsa-and-azide-free-ab236066.html
CD74 LN2 abcam ab213104 GR3359511-2 https://www.abcam.com/cd74-antibody-ln-2-bsa-and-azide-free-ab213104.html
PDGFRbeta Polyclonal R&D Systems AF385 B1W0821071 https://www.rndsystems.com/products/human-pdgf-rbeta-antibody_af385
GLUT1 (Glucose Transporter) EPR3915 abcam ab252403 GR3374509-1 https://www.abcam.com/glucose-transporter-glut1-antibody-epr3915-bsa-and-azide-free-ab252403.html
HLA- DRB5 Polyclonal Thermo Fisher PA5-60260 UL2898503A https://www.thermofisher.com/antibody/product/HLA-DRB5-Antibody-Polyclonal/PA5-60260
HLA- DRA Polyclonal Thermo Fisher PA5-27553 UL2898347B https://www.thermofisher.com/antibody/product/HLA-DRA-Antibody-Polyclonal/PA5-27553

HLA- DPA1 Polyclonal Thermo Fisher PA5-28037 UL2898341 https://www.thermofisher.com/antibody/product/HLA-DPA1-Antibody-Polyclonal/PA5-28037
HLA- DRB1 EPR6148 abcam ab133578 GR3300630-2 https://www.abcam.com/hla-class-ii-drb1-antibody-epr6148-ab133578.html
HLA- DQB1 Polyclonal abcam ab224600 GR3231742-9 https://www.abcam.com/hla-dqb1-antibody-ab224600.html
HLA- DMA Polyclonal Thermo Fisher PA5-22365 WB3188332C https://www.thermofisher.com/antibody/product/HLA-DMA-Antibody-Polyclonal/PA5-22365
Mouse IgG (H+L) Polyclonal Thermo Fisher A28174 2276480 https://www.thermofisher.com/antibody/product/Goat-anti-Mouse-IgG-H-L-Secondary-Antibody-Recombinant-Polyclonal/A28174
Rabbit IgG (H+L) Polyclonal Thermo Fisher A27033 RL246119A https://www.thermofisher.com/antibody/product/Goat-anti-Rabbit-IgG-Heavy-Chain-Secondary-Antibody-Recombinant-Polyclonal/A27033
Anti-Rat IgG (H+L) Polyclonal Thermo Fisher A18873 61-172-060320 https://www.thermofisher.com/antibody/product/Goat-anti-Rat-IgG-H-L-Cross-Adsorbed-Secondary-Antibody-Polyclonal/A18873

Validation

Validation are available for all antibodies from the manufacturer. Please refer to references contained in the provided links.

Human research participants

Policy information about studies involving human research participants

Population characteristics

We performed single-cell RNA sequencing of 606,380 freshly isolated endothelial, perivascular and other tissue-derived cells from 117 samples, from 68 human fetuses and adult patients to construct amolecular atlas of the developing fetal, adult control and diseased human brain vasculature.
Tissues analyzed include fetal and adult samples covering fetal CNS and peripheral organs; as adult/control brains (temporal lobe (TL)), brain arteriovenous malformation (AVM), lower-grade glioma (LGG), high-grade gliomas/glioblastoma (GBM), lung cancer brain metastasis (MET) and meningioma (MEN).

Recruitment

Informed consent for fetal tissue collection and research was obtained from each patient after her decision to legally terminate her pregnancy but before the abortive procedure was performed. For adult tissue collection, informed consents for collection and research use of the surgically removed adult brain tissues was obtained from each patient before the operation.

Ethics oversight

The collection of human samples and research conducted in this study were approved by the institutional research ethics review boards of the University Hospital Zurich, the University Health Network Toronto and the Mount Sinai Hospital Toronto (approval numbers: BASEC 2016-00167, 13-6009, 20-0141-E). Informed consent for fetal tissue collection and research was obtained from each patient after her decision to legally terminate her pregnancy but before the abortive procedure was performed. For adult tissue collection, informed consents for collection and research use of the surgically removed adult brain tissues was obtained from each patient before the operation. Details on patient information and pathology reports are provided in Supplementary Tables 1,2. All the protocols used in this study were in strict compliance with the legal and ethical regulations of the University of Zurich, the University of Toronto and affiliated hospitals.

Note that full information on the approval of the study protocol must also be provided in the manuscript.

Clinical data

Policy information about clinical studies
All manuscripts should comply with the ICMJE guidelines for publication of clinical research and a completed CONSORT checklist must be included with all submissions.

Clinical trial registration: NA

Study protocol: NA

Data collection: NA

Outcomes: NA

Flow Cytometry

Plots

Confirm that:

☒ The axis labels state the marker and fluorochrome used (e.g. CD4-FITC).

☒ The axis scales are clearly visible. Include numbers along axes only for bottom left plot of group (a 'group' is an analysis of identical markers).

☐ All plots are contour plots with outliers or pseudocolor plots.

☒ A numerical value for number of cells or percentage (with statistics) is provided.

Methodology

Sample preparation

For human fetal brains/CNS and human fetal peripheral organ tissues, fresh fetal tissues were obtained from patients who

Sample preparation

elected to terminate their pregnancies at fetal age (indicated by gestational weeks) 9 weeks – 21 weeks (GW 9 - 21) for reasons that are not genetic or medical conditions. Immediately following the termination of pregnancy procedure, fetal tissue samples were transferred to cold 0.01 M PBS / surgical physiological solution (Tis-U-Sol, Baxter) and transported on ice to the research facility in order to begin tissue processing and sample dissociation within 3 hours of collection.
For human adult/control brains (temporal lobe, TL), fresh normal cerebral cortex of the temporal lobe was obtained as part of a neurosurgical operation for epilepsy called temporal lobectomy1 for patients with pharmacoresistant epilepsy. Brain tissue of the neocortical resection of the temporal lobectomy corresponding to the normal cerebral cortex/neocortex overlying the hippocampus (to reach deep seated lesions in the amygdala and hippocampus causing epilepsy and removed during the amygdalohippocampectomy resection of the temporal lobectomy) which is thought to be uninvolved in the pathology2 (for details about the tissue samples, see Supplementary Tables 1,2) was harvested. We harvested the maximal safe amount of temporal neocortex after a piece of tissue was sent for histopathology (standard for every neurosurgical operation). All harvested brain specimens were >2 cm away from any radiographic abnormality on magnetic resonance imaging.
For human brain pathologies (brain tumors (LGG, GBM, MET, MEN) and brain vascular malformations (AVM), fresh samples were obtained during neurosurgical operations for either brain tumors or brain vascular malformations (for details about the tissue samples, see Supplementary Tables 1,2). In brief, we harvested the maximal safe amount of brain tumor/brain vascular malformation tissue after a piece of tissue was sent for histopathology (standard for every neurosurgical operation), (for details about the tissue samples, see Supplementary Tables 1,2).
All adult brain tissues (adult/control brain tissue, pathological brain tissues) were acquired by academic neurosurgeons familiar with routine sampling of surgical tissue for research purposes including cell isolations and sequencing experiments as well as tissue stainings. Generally, this involved i) resection “en bloc” (e.g. the entire brain tumor / brain vascular malformation, the entire neocortex overlying the hippocampal lesion) to maintain in situ tissue organization whenever possible ii) minimizing tissue damage/disruption by avoidance of electrocautery as much as possible, iii) washing the brain tumor or perfusing the brain vascular malformation tissue with cold 0.01 M PBS/surgical physiological solution (Tis-U-Sol, Baxter) to reduce intravascular blood/erythrocytes. In the case of brain arteriovenous malformations 1:9 heparin dilution was added to the physiological solution to perfuse the vessels. Immediately after neurosurgical resection, adult tissue samples were transferred to cold 0.01 M PBS / surgical physiological solution (Tis-U-Sol, Baxter) and transported on ice to the research facility in order to begin tissue processing and sample dissociation within 2-3 hours of resection. Fetal and adult tissue samples were then processed for either bulk- or sc-RNA sequencing (FACS-sorted or unsorted) or for immunofluorescence (IF), immunohistochemistry (IHC) or Imaging Mass Cytometry (IMC). Patient demographic information and details on brain tissue samples for all fetal brain and peripheral tissues as well as for all resected adult/control brain and pathological brain tissues utilized are summarized in Supplementary Tables 1,2).

Isolation of FACS-sorted human fetal and adult endothelial cells and of unsorted human fetal and adult endothelial and perivascular cells for single cell RNA-seq
Endothelial cells were isolated from human fetal and adult tissues using tissue digestion and subsequent CD31+ / CD45- FACS sorting whereas human endothelial and perivascular cells (all cells) were isolated from the unsorted fraction. Briefly, both fetal and adult tissues were quickly minced in a petri dish on ice, using two surgical blades. For CD31+ / CD45- FACS sorting, a cell suspension was obtained upon digesting the tissue in 2 mg/ml Dispase II (D4693, Sigma-Aldrich, Steinheim, Germany), 2 mg/ml Collagenase IV (#1710401, Thermo Fisher Scientific, Zurich, Switzerland) and 2 mM CaCl2 PBS solution for 40 min at 37°C with occasional shaking. The suspension was filtered sequentially through 100/70/40 µm cell strainers (#431751, Corning, New York, USA) to remove large cell debris. Cells were then centrifuged 500 RCF for 5 min at 4°C. In case of a visible myelin pellet 5 ml 25% Bovine Serum Albumin (BSA) (ice cold) was overlayed with 5 ml of the sample, centrifuged at 2000 RCF for 20 min (4°C). The supernatant was removed (including the lipid phase) and the pellet was resuspended in 9 ml PBS, followed by another round of centrifugation at 500 RCF for 5 min (4°C). Supernatant was subsequently discarded, while the cell pellets were resuspended in 3 ml of ACK hemolytic buffer at room temperature for 3 minutes. To stop the reaction, 30 ml of ice-cold PBS was added to the mixture and centrifuged at 500 RCF for 5 min at 4ºC. The cell pellets were resuspended in FACS buffer (PBS + 1% Bovine Serum Albumin), a volume is taken for unsorted scRNA-seq analysis. For CD31+ / CD45- FACS sorting, the cells were stained with anti-CD31 PE conjugated antibody in a concentration of 1:20 (#566125, clone MBC78.2, BD Pharmingen) and anti-CD45 APC conjugated antibody in a concentration of 1:20 (#17-0459-42, clone HI30, eBiosciences) for 30 min at 4º C, protected from light. Thereafter the cells were washed with 1ml of FACS buffer, centrifuged in a tabletop centrifuge at 500 RCF at 4ºC for 5 min. Finally, the cell pellets were resuspended in appropriate volumes of FACS buffer (PBS + 1% Bovine Serum Albumin) and the suspension was passed through a 35 µm cell strainer of a FACS sorting tube (#352235, Corning). Immediately before sorting, SYTOXTM blue was added in 1:1000 (Thermo Fisher Scientific, #S34857) to exclude dead cells from further analysis. Cell debris were excluded via a forward scatter-area/side scatter-area (FSC-A/SSC-A) gating, while singlets were selected for using a forward scatter-area (FSC-A)/ FSC-height (FSC-H) gating strategy. Viable (SYTOXTM blue negative) endothelial cells were FACS-sorted by endothelial marker CD31 positivity and negative selection for the brain microglia and macrophages marker CD45, whereas unsorted endothelial and perivascular cells were obtained from the SYTOXTM blue- fraction. Cells were sorted by a FACS Aria III (BD Bioscience) sorter using the four-way purity sorting mode directly in EGM2 medium (#CC-3162, Lonza, Basel, Switzerland).

Instrument

FACS Aria III

Software

FACS Aria III software

Cell population abundance

The abundance of endothelial cells (CD31+/CD45-) varied according to the patient sample, ranging from 5% to 15% of all viable cells.

Gating strategy

Cell debris were excluded via a forward scatter-area/side scatter-area (FSC-A/SSC-A) gating, while singlets were selected for using a forward scatter-area (FSC-A)/ FSC-height (FSC-H) gating strategy. Viable (SYTOXTM blue negative) endothelial cells were FACS-sorted by endothelial marker CD31 positivity and negative selection for the brain microglia and macrophages marker CD45, whereas unsorted endothelial and perivascular cells were obtained from the SYTOXTM blue- fraction. Cells were sorted by a FACS Aria III (BD Bioscience) sorter using the four-way (Figure exemplifying the gating strategy is provided in Supplementary Figure 1p).

☒ Tick this box to confirm that a figure exemplifying the gating strategy is provided in the Supplementary Information.
