# Supplementary material for: Single-cell atlas of the human brain vasculature across development, adulthood and disease
Source: Nature. 2024 Jul 10;632(8025):603–13. doi: 10.1038/s41586-024-07493-y (PMC11324530; doi:10.1038/s41586-024-07493-y)
Supplement: Supplementary file 4 — A detailed description of the methods used in our article with a separate references section. [file 41586_2024_7493_MOESM4_ESM.docx]

**METHODS**

**Ethics statements**

The collection of human fetal and adult samples and research conducted in this study were approved by the institutional research ethics review boards of the University Hospital Zurich, the University Health Network Toronto and the Mount Sinai Hospital, Sinai Health Systems Toronto (approval numbers: BASEC 2016-00167, REB 13-6009, REB 20-0141-E, REB 20-0178-E). Informed consent for fetal tissue collection and research was obtained from each patient after her decision to legally terminate her pregnancy but before the abortive procedure was performed. For adult tissue collection, informed consents for collection and research use of the surgically removed adult brain tissues was obtained from each patient before the operation. Details on patient information and pathology reports are provided in Supplementary Tables 1,2. All the protocols used in this study were in strict compliance with the legal and ethical regulations of the University of Zurich, the University of Toronto and affiliated hospitals.

**Acquisition and processing of human fetal and adult tissues**

For human fetal brains/CNS and human fetal peripheral organ tissues, fresh fetal tissues were obtained from patients who elected to terminate their pregnancies at fetal age (indicated by gestational weeks) 9 weeks – 21 weeks (GW 9 - 21) for reasons that are not genetic or medical conditions. Immediately following the termination of pregnancy procedure, fetal tissue samples were transferred to cold 0.01 M PBS / surgical physiological solution (Tis-U-Sol, Baxter) and transported on ice to the research facility in order to begin tissue processing and sample dissociation within 3 hours of collection.

For human adult/control brains (temporal lobe, TL), fresh normal cerebral cortex of the temporal lobe was obtained as part of a neurosurgical operation for epilepsy called temporal lobectomy^1^ for patients with pharmacoresistant epilepsy. Brain tissue of the neocortical resection of the temporal lobectomy corresponding to the normal cerebral cortex/neocortex overlying the hippocampus (to reach deep seated lesions in the amygdala and hippocampus causing epilepsy and removed during the amygdalohippocampectomy resection of the temporal lobectomy) which is thought to be uninvolved in the pathology^2^ (for details about the tissue samples, see Supplementary Tables 1,2) was harvested. We harvested the maximal safe amount of temporal neocortex after a piece of tissue was sent for histopathology (standard for every neurosurgical operation). All harvested brain specimens were >2 cm away from any radiographic abnormality on magnetic resonance imaging.

For human brain pathologies (brain tumors (LGG, GBM, MET, MEN) and brain vascular malformations (AVM), fresh samples were obtained during neurosurgical operations for either brain tumors or brain vascular malformations (for details about the tissue samples, see Supplementary Tables 1,2). In brief, we harvested the maximal safe amount of brain tumor/brain vascular malformation tissue after a piece of tissue was sent for histopathology (standard for every neurosurgical operation), (for details about the tissue samples, see Supplementary Tables 1,2).

All adult brain tissues (adult/control brain tissue, pathological brain tissues) were acquired by academic neurosurgeons familiar with routine sampling of surgical tissue for research purposes including cell isolations and sequencing experiments as well as tissue stainings. Generally, this involved i) resection “en bloc” (e.g. the entire brain tumor / brain vascular malformation, the entire neocortex overlying the hippocampal lesion) to maintain in situ tissue organization whenever possible ii) minimizing tissue damage/disruption by avoidance of electrocautery as much as possible, iii) washing the brain tumor or perfusing the brain vascular malformation tissue with cold 0.01 M PBS/surgical physiological solution (Tis-U-Sol, Baxter) to reduce intravascular blood/erythrocytes. In the case of brain arteriovenous malformations 1:9 heparin dilution was added to the physiological solution to perfuse the vessels. Immediately after neurosurgical resection, adult tissue samples were transferred to cold 0.01 M PBS / surgical physiological solution (Tis-U-Sol, Baxter) and transported on ice to the research facility in order to begin tissue processing and sample dissociation within 2-3 hours of resection. Fetal and adult tissue samples were then processed for either bulk- or sc-RNA sequencing (FACS-sorted or unsorted) or for immunofluorescence (IF), immunohistochemistry (IHC) or Imaging Mass Cytometry (IMC). Patient demographic information and details on brain tissue samples for all fetal brain and peripheral tissues as well as for all resected adult/control brain and pathological brain tissues utilized are summarized in Supplementary Tables 1,2).

**Isolation of FACS-sorted human fetal and adult endothelial cells and of unsorted human fetal and adult endothelial and perivascular cells for single cell RNA-seq**

Endothelial cells were isolated from human fetal and adult tissues using tissue digestion and subsequent CD31^+^ / CD45^-^ FACS sorting whereas human endothelial and perivascular cells (all cells) were isolated from the unsorted fraction. Briefly, both fetal and adult tissues were quickly minced in a petri dish on ice, using two surgical blades. For CD31^+^ / CD45^-^ FACS sorting, a cell suspension was obtained upon digesting the tissue in 2 mg/ml Dispase II (D4693, Sigma-Aldrich, Steinheim, Germany), 2 mg/ml Collagenase IV (#1710401, Thermo Fisher Scientific, Zurich, Switzerland) and 2 mM CaCl2 PBS solution for 40 min at 37°C with occasional shaking. The suspension was filtered sequentially through 100/70/40 μm cell strainers (#431751, Corning, New York, USA) to remove large cell debris. Cells were then centrifuged 500 RCF for 5 min at 4°C. In case of a visible myelin pellet 5 ml 25% Bovine Serum Albumin (BSA) (ice cold) was overlayed with 5 ml of the sample, centrifuged at 2000 RCF for 20 min (4°C). The supernatant was removed (including the lipid phase) and the pellet was resuspended in 9 ml PBS, followed by another round of centrifugation at 500 RCF for 5 min (4°C). Supernatant was subsequently discarded, while the cell pellets were resuspended in 3 ml of ACK hemolytic buffer at room temperature for 3 minutes. To stop the reaction, 30 ml of ice-cold PBS was added to the mixture and centrifuged at 500 RCF for 5 min at 4⁰C. The cell pellets were resuspended in FACS buffer (PBS + 1% Bovine Serum Albumin), a volume is taken for unsorted scRNA-seq analysis. For CD31^+^ / CD45^-^ FACS sorting, the cells were stained with anti-CD31 PE conjugated antibody in a concentration of 1:20 (#566125, clone MBC78.2, BD Pharmingen) and anti-CD45 APC conjugated antibody in a concentration of 1:20 (#17-0459-42, clone HI30, eBiosciences) for 30 min at 4⁰ C, protected from light. Thereafter the cells were washed with 1ml of FACS buffer, centrifuged in a tabletop centrifuge at 500 RCF at 4⁰C for 5 min. Finally, the cell pellets were resuspended in appropriate volumes of FACS buffer (PBS + 1% Bovine Serum Albumin) and the suspension was passed through a 35 μm cell strainer of a FACS sorting tube (#352235, Corning). Immediately before sorting, SYTOX^TM^ blue was added in 1:1000 (Thermo Fisher Scientific, #S34857) to exclude dead cells from further analysis. Cell debris were excluded via a forward scatter-area/side scatter-area (FSC-A/SSC-A) gating, while singlets were selected for using a forward scatter-area (FSC-A)/ FSC-height (FSC-H) gating strategy. Viable (SYTOX^TM^ blue negative) endothelial cells were FACS-sorted by endothelial marker CD31 positivity and negative selection for the brain microglia and macrophages marker CD45, whereas unsorted endothelial and perivascular cells were obtained from the SYTOX^TM^ blue**^-^** fraction. Cells were sorted by a FACS Aria III (BD Bioscience) sorter using the four-way purity sorting mode directly in EGM2 medium (#CC-3162, Lonza, Basel, Switzerland).

**Chromium 10X library preparation, single cell RNA-sequencing and data analysis**

CD31^+^ / CD45^-^ FACS sorted endothelial cells and unsorted endothelial and perivascular cells were resuspended in PBS supplemented with Bovine Serum Albumin (BSA) (400 µg/ml, Thermo Fisher Scientific#37525), targeting the required a 1,000 cells/μl concentration and loaded on the chromium controller (10X genomics). Single-cell RNA-seq libraries (chromium next GEM single cell 3' gene expression libraries) were obtained following the 10x Genomics recommended protocol, using the reagents included in the Chromium Single Cell v3 Reagent Kit. Quality control of cDNA and final libraries was done using 4200 TapeStation System (Agilent) and D5000 ScreenTape & Reagents. Libraries were sequenced on the NextSeq 500 (Illumina) instrument, aiming at 50k reads per cell. The 10x Genomics scRNA-seq data was processed using cellranger-5.0.0 with the Homo sapiens Gencode GRChm38.p13 genome Ensembl release. Based on filtered gene-cell count matrix by CellRanger’s default cell calling algorithm, we performed the standard Seurat clustering (version 4.0.0 in R 4.2.2) workflow, as described below; raw expression values were normalized and log transformed (normalization.method = "LogNormalize"). In order to exclude low quality cells and doublets, cells with less than 500 or more than 3000 detected genes were filtered out. scDblFinder (v3.13) was used to validate that doublets were minimal. We also filtered cells with > 25% mitochondrial counts (Supplementary Tables 3,4).

**Integration/batch correction at the level of the overall merge of sorted endothelial cells of all entities**

For integration /batch correction of the overall merge of sorted endothelial cell datasets of all entities, Seurat's reciprocal PCA (RPCA) was applied, consisting of the following computational steps: i) in brief, the samples (every sample consisting of a "patient") to be integrated/batch corrected were merged using the Seurat function "merge". ii) The merged dataset was split by "patient" using the function "SplitObject". iii) For each "patient" independently, the normalization was done using the function "NormalizeData", then highly variable features (= genes) were computed using the Seurat function "FindVariableFeatures", returning 3000 features by patient, which were then used for the downstream PCA analysis. iv) The features that were repeatedly variable across the different patients were selected using the "SelectIntegrationFeatures" function. v) Integration anchors were then identified using the "FindIntegrationAnchors" function, which takes the list of Seurat objects of the different patients to integrate as input, reduction was set to "rpca", and then used these anchors to integrate the data together using "IntegrateData". After this step, the integration of the dataset is achieved. Next, to perform downstream analyses on this integrated dataset including clustering and UMAP visualization, the following steps were performed: i) The resulting integrated data was scaled using the "ScaleData" function. ii) next, PCA (using "RunPCA" function) was performed on the scaled data. iii) The principal components (PC) were ranked based on the percentage of variance (using "ElbowPlot" function). iv) To define the endothelial clusters and perform UMAP visualization – provided that the 'elbow' was observed around PC30 - the top 30 PCs were used as inputs for the Seurat functions "FindNeighbors", "FindClusters" (resolution 2) and "RunUMAP". This resulted in 44 endothelial Seurat clusters. For endothelial cell cluster annotation, please see section "Cell cluster annotation".

**Integration/batch correction of sorted endothelial cells at the level of individual entities separately for every entity**

To visualize the UMAPs of endothelial cells for every individual entity separately – notably fetal brains, adult/control brains (TLs), arteriovenous malformations (AVMs), lower grade gliomas (LGGs), high grade gliomas/glioblastomas (GBMs), brain metastases (METs) and meningiomas (MENs) – integration of endothelial cells of patients per individual entity was performed. The integration/batch correction was performed using Seurat's reciprocal PCA (RPCA) and applied exactly the same way as described in the section: "*Integration/batch correction at the level of the overall merge of sorted endothelial cells of all entities*".

**Integration/batch correction of unsorted cells at the level of individual entities separately for every entity**

For cell annotation as well as UMAP visualization of unsorted cells for every individual entity separately – notably fetal brains, adult/control brains (TLs), arteriovenous malformations (AVMs), lower grade gliomas (LGGs), high grade gliomas/glioblastomas (GBMs), brain metastases (METs) and meningiomas (MENs) – integration of unsorted cells of patients per individual entity was performed. The integration/batch correction was performed using Seurat's reciprocal PCA (RPCA) and applied exactly the same way as described in the section: "*Integration/batch correction at the level of the overall merge of sorted endothelial cells of all entities*".

**Integration/batch correction at the level of the overall merge of unsorted cells of all entities**

The integration/batch correction was not performed at the level of the overall merge of unsorted cells across all entities because we did not have biological questions that would require to do so. However, in order to respond to the reviewer's comments, we preformed the overall merge of unsorted cells across all entities using different methods of integration/batch correction, notably RPCA, CCA, Harmony, and scANVI (Reviewer Figure 5).

**Comparison to other integration/batch correction methods**

In order to validate the integration results obtained by Seurat reciprocal PCA (RPCA), integration/batch correction was also performed with:

1) Harmony integration/batch correction(<https://github.com/immunogenomics/harmony>) (REF: <https://www.nature.com/articles/s41592-019-0619-0>) was applied consisting of the following computational steps: i) in brief the samples to be integrated were merged using the Seurat function "merge", thereafter the data were normalized using "NormalizeData", and the highly variable features were computed using "FindVariableFeatures" function. ii) downstream PCA analysis done using "RunPCA", iii) data was scaled using the "ScaleData" function. iv) harmony integration was performed using "RunHarmony" function, "group.by.vars" was set to include "patient","sex","age". v) Harmony embeddings were used for the downstream "RunUMAP" and "FindNeighbors" functions. vi) Finally the clusters were identified using the "FindClusters" function. For endothelial cell cluster annotation, please see section "Cell cluster annotation".

2) Seurat’s canonical correlation analysis (CCA) integration/batch correction

CC integration was applied consisting of the following computational steps: i) in brief the samples (every sample consisting of a "patient") to be integrated/batch corrected were merged using the Seurat function "merge". ii) The merged dataset was split by "patient" using the " SplitObject" function. iii) For each patient independently, the normalization was done using "NormalizeData" function, then highly variable features (=genes) were computed using Seurat's "FindVariableFeatures" function, returning 3000 features, which were used for the downstream PCA analysis. iv) The features that were repeatedly variable across the different patients were selected using the "SelectIntegrationFeatures" function. v) Integration anchors were then identified using "FindIntegrationAnchors" function, which takes the list of Seurat objects of the different patients to integrate as input and then used these anchors to integrate the data together using "IntegrateData". After this step, the integration of the dataset is achieved. Next, to perform downstream analyses on this integrated dataset including clustering and UMAP visualization, the following steps were performed: i) The resulting integrated data was scaled using the "ScaleData" function. ii) next, PCA was performed (using "RunPCA" function) on the scaled data. iii) The principal components (PC) were ranked based on the percentage of variance (using "ElbowPlot" function).

iv) To define the endothelial clusters and perform UMAP visualization – provided that the 'elbow' was observed around PC30 - the top 30 PCs were used as inputs for the Seurat functions "FindNeighbors", "FindClusters" and "RunUMAP". For endothelial cell cluster annotation, please see section "Cell cluster annotation".

3) scANVI

scANVI (https://github.com/scverse/scvi-tools) integration incorporates cell type annotation information (obtained from RPCA integration). In brief, the integration was performed as follows: i) the highly variable genes were obtained for each patient and their intersection was taken via "sc.pp.highly_variable_genes" function. ii) Then an SCVI model object (which aims to finds common axes of variation in the different patient samples) is created via the "scvi.model.SCVI.setup_anndata", "scvi.model.SCVI" and "vae.train()" functions. iii) Once the training is done, the latent representation of each cell in the dataset is evaluated and added to the dataset via "adata.obsm["X_scVI"] = vae.get_latent_representation()". iv) Finally, the dataset was clustered by running the functions: "sc.pp.neighbors", "sc.tl.leiden" and "sc.tl.umap". v) scANVI was then initialized from the scVI model pre-trained on the dataset via running the functions "lvae = scvi.model.SCANVI.from_scvi_model" and "lvae.train". vi) The latent representation of each cell in the dataset was then retrieved and added to the dataset via "adata.obsm["X_scANVI"] = lvae.get_latent_representation(adata)".

Heatmap of the pairwise Jaccard distance between the ECs annotated via the different integration methods was computed using scclusteval package (Extended Data Figure 5)^3^, Quality control metrics (Modified average silhouette width (ASW)) to compare the different methods of integration/batch correction were performed using scIB package (Supplementary Table 27)^4^.

**Cell cluster annotation**

For cluster annotation, differential expression analysis for each cluster against all other clusters was computed using wilcoxauc function implemented in the github package presto (v1.0.0) (https://github.com/immunogenomics/presto) (FDR values were calculated using the Benjamini–Hochberg method). The resulting positive markers (that passed the threshold of p value < 0.05, and log2FC > 0.25) were used as the top cluster markers for annotation. For the unsorted and sorted samples, EC and PVC cell types (unsorted samples) and EC clusters (sorted samples) were annotated referring to different published papers as well as to canonical PVC and EC markers^5-9^. To find human-specific, mouse-specific and species conserved EC and PVC markers as well as EC subtype markers, we compared our cell type/AV cluster markers to the corresponding ones derived from^5,7,10^.

Heatmap of the pairwise Jaccard distance between the 14 main EC clusters and 44 EC subclusters was computed using scclusteval package^3^. BIOMEX (version 1.0-4) (<https://carmelietlab.sites.vib.be/en/biomex>) was using to perform the cluster similarity analysis (Figure 3m-p) as described in sections 33 and 37 of the package manual.

To predict the cell identity of the pathological brain endothelial cell clusters of the adult and fetal datasets as compared to the adult/control brain endothelial cells, the cell identity classification and label transfer was done using the standard Seurat workflow using the temporal lobe endothelial cells. as the reference dataset. Illustration of the results was generated using Seurat (v.4.0.0), Sankey plots were done using networkD3 (v.0.4).

For patient level analysis, DESeq2 package (v1.30.1) was used to perform pseudobulk differential expression analysis, volcano plots were done using EnhancedVolcano (v1.8.0) package and heatmaps were plotted using pheatmap (v1.0.12).

**Compositional analysis**

To statistically quantify the compositional changes between fetal, adult/control and pathological brain datasets, we used single-cell differential composition analysis (scCODA) package (version 0.1.7) ^11^ (python 3.7).

Moreover, to validate scCODA findings while adjusting for covariates age and sex, we used: 1) tree-aggregated amplicon and single-cell compositional data analysis (tascCODA) (version 0.1.3) (https://github.com/bio-datascience/tascCODA)^12^ which is a fully Bayesian model for tree-aggregated modeling of count data and is a natural extension of the scCODA model. tascCODA enables to determine how disease status, host covariates such as age, sex, jointly influence the compositional counts. For endothelial cell subtype analysis, the formula "proportions ~ Pathology + sex + age" was used in the "ana.CompositionalAnalysisTree" function, the adult/control brain (temporal lobe) was used as a reference.

2) Dirichlet regression was used via DirichletReg R package (version 0.7.0) (https://github.com/maiermarco/DirichletReg)^13^. For endothelial cell subtype analysis, the formula "proportions ~ Pathology + sex + age" was used with alternative parametrization in the "DirichReg" function, where the adult/control brain (temporal lobe) was used as a reference.

3) The propeller method was used via speckle R package (0.99.1) (<https://github.com/phipsonlab/speckle>)^14^, which accommodates complex experimental designs enabling modeling of covariates age and sex. Th design matrix was set to "model.matrix(~ 0 + Pathology + pair + sex +age)" in the "propeller.ttest" function.

4) Single-Cell Interpretable Tensor Decomposition (scITD) (version 1.0.2) (<https://github.com/kharchenkolab/scITD#walkthrough>)^15^, was used to assess if there are interpatient factors are significantly associated with covariates: age, sex, age, MGMT methylation status (for GBMs) and lung brain metastasis subtype. In brief, a project container containing the metadata and counts matrix is set-up using the function "make_new_container". Next the data is formed into a tensor with dimensions donors x genes x cell-types via the "from_tensor" function. Thereafter the Tucker decomposition was run using "run_tucker_ica". Finally the donor score matrix is plotted by running the functions: "get_meta_associations", "plot_donor_matrix" and "container$plots$donor_matrix".

**Bulk RNA-sequencing and analysis**

For bulk RNA-seq analysis the cells were sorted directly into RLT Plus lysis buffer and RNA was extracted using RNeasy Plus Micro Kit (Cat No. 74034, Qiagen, Hilden, Germany). RNA sequencing of endothelial cells was performed by the Functional Genomics Center Zurich. The libraries were prepared following Illumina TruSeq stranded mRNA protocol. The quality of the RNA and final libraries was determined using an Agilent 4200 TapeStation System. The libraries were pooled equimolarly and sequenced in an Illumina NovaSeq sequencer (single-end 100 bp) with a depth of around 20 Mio reads per sample. For mapping and trimming of FASTQ format sequences was performed using Trimmomatic v0.3.3, and sequence quality control was assessed using FastQC. Alignment to the Ensembl Homo_sapiens GRCh38.p10 reference genome (Release_91-2018-02-26) was performed using the STAR aligner. Gene expression values were computed with the function featureCounts from the R package Rsubread. Differential expression was computed using the generalized linear model implemented in the Bioconductor package DESeq2. FDR values were calculated using the Benjamini–Hochberg method. Sorted endothelial cells bulk RNA-seq deconvolution was performed using BayesPrism (version 2.0) (https://github.com/Danko-Lab/BayesPrism)^16^ and EPIC (version 1.1.5) (https://github.com/GfellerLab/EPIC)^17^ R packages (Supplementary Table 26).

**Pathway analysis using GSEA and cytoscape**

Differential expression was computed using the wilcoxauc function implemented in the github package presto (https://github.com/immunogenomics/presto). FDR values were calculated using the Benjamini–Hochberg method.
Pathway analysis was performed on the ranked differential expression gene list (ranked by the formula:"*-LOG10(pvalue)*SIGN(logFC)*") using the Gene Set Enrichment Analysis (GSEA) (classic enrichment statistic) software from the Broad Institute (software.broadinstitute.org/GSEA) (version 4.0.1)^18,19^. A permutation-based *P*-value is computed and corrected for multiple testing to produce a permutation based Benjamini – Hochberg correction false-discovery rate q-value that ranges from 1 (not significant) to 0 (highly significant). The resulting pathways were ranked using NES and FDR q-value, *P*-values were reported in the GSEA output reports.

"Human_GOBP_AllPathways_no_GO_iea_March_01_2021_symbol.gmt" from [http://baderlab.org/GeneSets] was used to identify enriched pathways in GSEA analysis (inclusion criteria are pathways with a minimum size of 15 and a maximum size of 300 genes). The resulting pathways were filtered based on passing the threshold of FDR<0.001 and pvalue<0.05. The resulting highly related pathways were grouped into a themes, labeled by AutoAnnotate (version 1.3) and plotted using Cytoscape (Version 3.7.0) and EnrichmentMap (version 3.3)^20^.

**Mapping to publicly available datasets**

To map our adult/control brain, AVM, GBM and MET datasets to the corresponding publicly available datasets, the cell identity classification and label transfer was done using the standard Seurat workflow; in brief, anchors are found between the reference datasets and our datasets (query datasets) using the "FindTransferAnchors" Seurat function, followed by using the "TransferData" function to classify the query cells based on the reference dataset. The "TransferData" function yields a matrix with predicted IDs and prediction scores, cells with prediction scores less than 0.5 are labelled as unassigned. The results were illustrated using Seurat (v.4.0.0) functions "Dimplot" to plot the reference based annotated UMAP and "FeaturePlot" to plot the prediction score UMAP while networkD3 (v.0.4) function “sankeyNetwork” was used to generate sankey plots.

**Definition of endothelial fetal/adult brain, AV, CNS, peripheral and MHC class II signatures and the BBB dysfunction modules**

Fetal/adult brain EC signature: we defined a human fetal/developmental and adult brain EC signature - revealing properties of the developing and mature human brain vasculature -comprising the top 50 the genes that passed the threshold of at least log2FC>0.25 and *p value*<0.05 enriched in fetal brain ECs compared with adult brain (TL) ECs and vice versa.

Arteriovenous signature: we defined a human AV-signature comprising genes revealing significant expression gradients along the arteriovenous axis in the adult/control brains, only genes that passed the threshold of at least log2FC>0.25 and *p value*<0.05 were used to construct the signature.

CNS and peripheral signatures: we defined a human adult and fetal – endothelial CNS and peripheral signatures comprising the top 50 the genes and at least twofold (log2FC>1.000) and *p value*<0.05 enriched in brain ECs compared with ECs of peripheral organs (heart, kidney, muscle and colon in our fetal dataset) and vice versa. For the adult endothelial CNS signature, adult/control brain ECs were compared to peripheral organ ECs in the dataset of Han et al^8^. For the mouse derived endothelial CNS and peripheral signatures, we used the genes described in Munji et al^21^ upon homology mapping using the function:

"convert_mouse_to_human_symbols" of the NicheNet R package (https://github.com/saeyslab/nichenetr).

MHC class II signature: we defined a human MHC class II signature comprising known MHC class II genes: *CD74*, *HLA-DRB5*, *HLA-DRB1*, *HLA-DQB1*, *HLA-DQB2*, *HLA-DPB1*, *HLA-DOB*, *HLA-DBM*, *HLA-DRA*, *HLA-DQA1*, *HLA-DQA2*, *HLA-DPA1*, *HLA-DOA* and *HLA-DMA*.

Blood-brain barrier (BBB) dysfunction module: The mouse BBB dysfunction module comprised the top 50 genes that are upregulated in CNS ECs upon various disease triggers (e.g. stroke, multiple sclerosis, traumatic brain injury and seizure) in the mouse brain and that shifts CNS ECs into peripheral endothelial cell-like states under these conditions^21^. Genes comprising this signature are implicated in such as cell division, blood vessel development, inflammatory response, wound healing, leukocyte migration and focal adhesion^21^. The human pathological brain, brain tumor, brain vascular malformation and brain AVM EC signatures/dysfunction modules were derived from differential expression analysis (comparing the pathological entity ECs to adult/control brain ECs) computed using the wilcoxauc function implemented in the github package presto. FDR values were calculated using the Benjamini–Hochberg method. The resulting positive markers (that passed the threshold of p value < 0.05, and log2FC > 0.25) were used to define the aforementioned signatures. The human Alzheimer’s disease EC dysfunction module was derived from Yang et al.^22^, the human Huntington’s disease EC dysfunction module from Garcia et al.^23^, and the human brain AVM signature (to which we compared our brain AVM signature) from Winkler et al.^2^

**Pseudospace/pseudotime trajectory analysis – Monocle and TSCAN**

To address pseudospace/pseudotime (AV-specification) trajectory analysis, we performed computational analyses using two published packages for cross validation, notably: Monocle 3 (version 1.0.0) (https://github.com/cole-trapnell-lab/monocle3) ^24,25^ and Tools for Single Cell Analysis^26^ (TSCAN) (1.36.0) (https://github.com/zji90/TSCAN).

Pseudospace/pseudotime analysis was performed using Monocle 3^24,25,27^ and TSCAN^26^ applying default parameters, in fetal, adult/control and pathological brain endothelial cells. Endothelial cells were clustered using the standard Seurat (version 4.0.0) clustering procedure (as detailed above) and cluster markers were used to AV annotate those clusters, which were used as an input into Monocle3 or TSCAN to infer trajectory/lineage/arteriovenous relationships within endothelial cells. SeuratWrappers (v.0.3.0) was used to convert the Seurat objects to cell data set objects, while retaining the Seurat generated UMAP embeddings and cell clustering and then trajectory graph learning and pseudo-time measurement with Monocle3 and TSCAN.

**Pseudotime trajectory analysis - RNA velocity and diffusion map**

To further address trajectory inference, we performed RNA velocity^28-30^ and diffusion map^31^ analyses, which both address the pseudotime but not the pseudospace.

To compute RNA velocity of endothelial cells of the different entities, Velocyto package (version 0.17.17) (https://velocyto.org/velocyto.py/)^28^ was used on the CellRanger output BAM files and the genome annotation (.gtf file) from (<https://support.10xgenomics.com/single-cell-gene-expression/software/pipelines/latest/advanced/references>). For RNA velocity visualization, the generated loom file containing the spliced and unspliced RNA transcripts served as input into the scVelo package (version 0.2.4) (<https://scvelo.readthedocs.io/en/stable/>, python 3.7)^29,32^. Trajectory inference was also analyzed using destiny package (version 3.12.0) (https://bioconductor.org/packages/release/bioc/html/destiny.html) ^31^, the method infers the low-dimensional manifold by estimating the eigenvalues and eigenvectors for the diffusion operator related to the data, In brief, the diffusion map was generated by applying the "DiffusionMap" function on the single cell experiment object.

**Cell-cell communication and ligand-receptor interaction analysis**

Cell-cell (ligand receptor) interaction analysis between endothelial cell (EC) clusters (EC – EC interactions) as well as between endothelial (EC) and perivascular cells (PVCs) (EC – PVC interactions) was performed using two published packages for cross validation, notably: CellPhoneDB (version 3.0.0) ^33^ and Cellchat (version 1.6.1) ^34^ applying default parameters. First, using CellphoneDB (version 3.0.0) ligand-receptor pairing matrix was constructed as follows; only ligands and receptors expressed in at least 10% of the cells in a particular cluster were considered, cluster labels were then permuted randomly 1,000 times to calculate the mean expression values of ligands and receptors, followed by pairwise comparisons between all cell types. The cut-off of expression was set to more that 0.1 and *P*-value to less than 0.05. The number of paired cell-cell interactions was based on the sum of the number of ligand-receptor interactions in each of the cell–cell pairs. Finally, Cytoscape was used to visualize the interaction network as a degree sorted circle layout^20^.

Second, using CellChat (v.1.6.1) we followed the developers’ suggested workflow, briefly applied the pre-processing functions “identifyOverExpressedGenes”, “identifyOverExpressedInteractions”, and “projectData” with standard parameters set. The CellChatDB including the Secreted Signaling pathways, ECM-receptor as well as Cell-Cell contact were analyzed, in addition MHC class-II interactions reported in the CellphoneDB. Moreover, the gene expression data was projected onto experimentally validated protein-protein interaction. The standard package functions as “computeCommunProb”, “computeCommunProbPathway” and “aggregateNet” were used with default parameters. Finally, to determine the ligand-receptor contributions and senders/receivers’ roles in the network the functions “netAnalysis_contribution” and “netAnalysis_signalingRole” was applied on the netP data slot respectively.

We further compared cell–cell communication patterns by computing the Euclidean distance between ligand-receptor pairs of the shared signaling pathways (a measure of the difference between the signaling networks of datasets, see methods e.g. larger Euclidean distance implying larger difference of the communication networks between two datasets in terms of either functional or structure similarity, termed network architecture)^34^. We compared the information flow for each signaling pathway between, which is defined by the sum of communication probability among all pairs of cell groups for a given signaling pathway in the inferred network^34^.

**Human tissue preparation for immunofluorescence and RNAscope**

Freshly resected adult tissue samples of brain pathologies or the neocortical part of temporal lobes of pharmacoresistant epilepsy patients were obtained from the Division of Neurosurgery, Toronto Western Hospital, University Health Network, University of Toronto and the Department of Neurosurgery, Zurich University Hospital, University of Zurich, whereas fetal tissue was obtained from the Research Centre for Women’s and Infants’ Health (RCWIH) BioBank. Sample age (gestational week for fetal tissues), gender and pathology were documented in Supplementary Tables 1,2. Fetal and adult tissues were fixed at 4°C in 4% paraformaldehyde (PFA) for 12 hours and placed into 30% sucrose in PBS solution overnight. The tissues were then embedded in Optimum Cutting temperature compound (Tissue-Tek O.C.T. Compound, #4583) and stored at -80°C. 4% Formalin fixed, cryo-embedded tissues were then processed for immunofluorescence and RNAscope.

**Immunofluorescence**

Formalin fixed, cryo-embedded fetal and adult tissues were cut in 40-µm thick sections, using a cryotome (Leica Cryostat 1720 Digital Cryotome), mounted onto microscope slides, and submitted for single and double staining with the antibodies provided in Supplementary Table 24. Briefly, the sections were: 1) antigen retrieval with 10mM Citric acid, pH 6.0, 0.05% Tween 20 at above 95°C for 10 minutes; 2) blocked with 10% donkey serum 0.3% Triton X-100 in PBS for 90 min at room temperature (RT); 3) incubated overnight at 4°C with primary Abs; 4) washed and incubated with the appropriate secondary Abs, donkey anti-mouse Alexa Fluor 488 (1:1000, Thermo Fisher Scientific, #A-21202), goat anti-mouse Alexa Fluor 647 (1:1000, Thermo Fisher Scientific, #A-21235, donkey anti-Guinea pig Alexa Fluor 488 (1:1000, Jackson Immunoresearch Labs, #706-545-148), donkey anti-rabbit Alexa Fluor 555 (1:1000, Thermo Fisher Scientific, #A-31572), donkey anti-goat Alexa Fluor 488 (1:1000, Thermo Fisher Scientific, #A-11055) for 90 min at RT; 5) to quench the autofluorescence signal, tissue sections were treated with 0.1% Sudan Black B (Thermo Fisher Scientific, Cat: J62268) in 70% ethanol for 30 minutes at room temperature. 6) counterstained with the 4, 6-diamidino-2-phenylindole (DAPI) (diluted 1:20,000; BioLegend, #422801) or Hoechst 33342 Solution (diluted 1:500; Thermo Fisher Scientific #62249). Finally, the sections on glass slides (Fisherbrand, Superfrost Plus Microscope Slides, Fisher Scientific) were mounted in ProLong™ Gold Antifade Mountant (Invitrogen #P36930) and coverslipped with VWR micro cover glass (VWR International). Negative controls were prepared by omitting the primary antibodies and mismatching the secondary antibodies. Sections were examined under Zeiss laser scanning confocal microscope (LSM 880) or Olympus FluoView Laser Scanning Confocal Microscope Olympus IX81 inverted stand. Laser scanning confocal images were taken through the z-axis of the section, with 20x and 40x lenses (LSM 880) or 40X objective lens Plan Apo 40x/1.35 NA oil immersion (IX81). Z-stacks of optical planes (maximum intensity projections) and single optical planes were recorded and analyzed by Zeiss Zen 2.3 software.

**RNAscope**

Formalin-fixed, cryo-embedded adult tissues were cut into 20-µm thick sections, mounted onto microscope slides, and subjected to RNAscope in-situ hybridization using the RNAscope HiPlex kit (324100-UM, ACD, Newark, CA) according to the manufacturer’s instructions. HiPlex probes were designed by ACD (Newark, CA). Briefly, on day one, fixed frozen tissue sections were baked for 30 minutes at 60°C, followed by 15 minutes incubation in pre-chilled 4% PFA at 4°C (secondary fixation). After fixation, tissue sections were dehydrated with subsequent incubations in 50%, 70%, 100% (twice) ethanol for 5 minutes each at room temperature (RT). Target retrieval was performed using 1X target retrieval buffer (Ref 322000; ACD, Newark, CA) and distilled water were heated to above 99°C. Slides were dipped in water for 10 seconds before placing into the 1X target retrieval buffer for 5 minutes. Then, slides were quickly washed in distilled water for 15 seconds. An additional Sudan Black step was added for tissues with high auto-immunofluorescence (AVM, LGG, TL, MEN). Slides were submerged in 0.1% Sudan Black in 70% ethanol for 30 minutes at RT, covered with foil. Then, slides were washed three times with distilled water, once in 100% ethanol and dried at 60°C for 5 minutes. Hydrophobic barrier was drawn around the sections and left to dry overnight. On day two, RNAscope Protease III (Ref 322340; ACD, Newark, CA) was added to cover each section and incubated at 40°C for 15 minutes inside the RNAscope HybEZ II oven. For hybridization, 1X probe mixture was added to each section and incubated at 40°C for 2 hours in the RNAscope HybEZ II Oven. For HiPlex Amp 1-3 hybridization, 1X Amp 1 solution was added to each section and incubated at 40°C for 30 minutes in the RNAscope HybEZ II Oven. Amp hybridization steps were sequentially repeated with Amp 2 and Amp 3 solutions. For HiPlex fluorophore hybridization, corresponding fluorophores (1X HiPlex T1-T4 solution) was added to each section and incubated at 40°C for 15 minutes in the RNAscope HybEZ II Oven. Wash steps with the appropriate buffers were done in between each step as indicated in the user manual. HiPlex Amp and fluorophore solutions were included in the RNAscope HiPlex8 Detection (Ref 324110; ACD, Newark, CA) and HiPlex12 Ancillary Kits (Ref 324120; ACD, Newark, CA). After fluorophore hybridization, DAPI (Ref 320858; ACD, Newark, CA) was added for 30 seconds at RT before being replaced with ProLong Gold Antifade Mountant (Ref P36930; Invitrogen, Waltham, MA). We used the following RNAscope probes: Hs-CD31 (Ref 548451-T3), Hs-ESM1 (Ref 586041-T7), Hs-ACTA2 (Ref 311811-T10), Hs-PLVAP (Ref 437461-T1), Hs-HLA-DPA1 (Ref 821641-T6), Hs-CD74 (Ref 477521-T11). Images were acquired using an Olympus FluoView Laser Scanning Confocal Microscope Olympus IX81 inverted stand; 40X objective lens Plan Apo 40x/1.35 NA oil immersion. Laser wavelengths were 405nm, 473nm, 559nm and 635nm. After the first round of imaging, slides were soaked in 4X SSC buffer (#BP1325-1; Fisher Scientific, Waltham, MA) until the cover slip could be removed easily. Fluorophores were cleaved with 10% cleavage solution (Ref 324130; ACD, Newark, CA) at RT for 15 minutes, followed by two washes with PBST (0.5% Tween 20) (repeated cleavage twice). Procedures for round 2 and 3 fluorophore hybridization were the same as the round 1. After three rounds of imaging, image alignment, merging and processing were performed using the RNAscope HiPlex Image Registration Software following the Image Registration Software User Manual (300065-UM) (ACD, Newark, CA).

Visualization was done using the FV10-ASW 4.2 Viewer and ImageJ^35^. Pseudocolors were used for better visualization.

**Human tissue preparation for Imaging Mass Cytometry (IMC)**

*For human adult/control brain and brain pathologies:*

Following tissue collection, tissues were immersion fixed in 10% neutral-buffered PFA for 24h at room temperature. Following the fixation period, tissues were transferred to a 70% ethanol solution and then placed into a VIP Tissue Tek tissue processor (Sakura Finetek USA, Inc). The tissue underwent increasing concentrations of Ethanol (70%-100%), followed by 3 changes of concentrated Xylene and finally perfused with histology grade molten paraffin wax. Tissues were embedded in Histology-grade paraffin and left to cool on a cold plate before long term storage at room temperature in a dark and dry location.

Sections were cut from paraffin blocks on a microtome set to a thickness of 4 microns and floated in a 42°C water bath of distilled water before transfer to ColorFrost glass slides. The slides were dried at 60°C, and then stored at room temperature until utilization. 10% FFPE tissue sections were then processed for Imaging Mass Cytometry.

*For human fetal brain/CNS tissues:*

Following tissue collection, tissues were immersion fixed in 4% neutral-buffered PFA for 24h at room temperature. Following the fixation period, tissues were transferred to a 70% ethanol solution and stored at 4 degrees until paraffin embedding. Fixed fetal tissue was embedded in IHC-grade paraffin and left to cool on a cold plate before long term storage at room temperature in a dark and dry location.

Sections were cut from paraffin blocks on a microtome set to a thickness of 5 microns and floated in a 40°C water bath of distilled water before transfer to ColorFrost / immunohistochemistry suitable glass slides. The slides were left to dry overnight, and then stored at room temperature until utilization.

To visualize CD31^+^ vessels on serial sections, slides were deparaffinised through xylenes and an alcohol gradient and taken to water. Antigen retrieval was performed using H.I.E.R. (Heat Induced Epitope Retrieval) with citrate buffer (pH 6). Endogenous peroxidase activity was blocked in Bloxall reagent (Agilent) for 10 minutes. Non-specific antibody binding was blocked using Vector ImmPRESS blocking reagent (MJS Biolynx, Cat. # MP-7401) for 20 minutes at room temperature. Sections were then incubated in primary antibody: Rabbit anti-CD31 (Abcam, Cat# ab28364) diluted 1:100 and incubated for 1 hour at room temperature. After washes in TBS-T, sections were incubated in ImmPRESS peroxidase Polymer Anti-Rabbit IgG reagent for 30 minutes. Positive immunoreactive staining was visualised using ImmPACT DAB (Vector Laboratories). Mayer’s hematoxylin was used as a counterstain prior to dehydrating and mounting coverslips. H&E stained slides were stained on an automated stainer following routine protocols. 4% FFPE tissue blocks were selected based on CD31 IHC stained serial sections and sections were then processed for Imaging Mass Cytometry.

**Imaging Mass Cytometry (IMC)** Human fetal CNS, human adult/control brain (temporal lobe) tissues, adult brain tumor tissues, and adult brain vascular malformation tissues were formalin-fixed (4% Paraformaldehyde) and paraffin-embedded, cut in 5µm thick sections and mounted onto microscope slides.

All fetal and adult brain tissue sections were dewaxed in xylene and rehydrated in a graded series of alcohol (100%, 100%, 96%, 90%, 80%, 70% ethanol). For heat-induced epitope retrieval, tissue sections were incubated in Tris-EDTA buffer (pH 9.2) within a decloaking chamber (Biocare Medical) for 30 minutes in 95^o^C. Tissue sections were then cooled and blocked in Tris buffered saline (TBS) containing 0.1% Tween20, 3% bovine serum albumin, and 5% goat serum. Tissue sections were then incubated with antibodies at a dilution of 5ug/ml overnight at 4^o^C. Tissues were washed two times in TBS + 0.1% Tween20 and two times in TBS before incubation with ^191^Ir/^193^Ir for 5 minutes in room temperature, washed again in TBS + 0.1% Tween20 and two times in TBS, and air-dried prior to imaging. Antibody information including metal tag, clone, company, catalog number, and lot number can be found in Supplemental Table 24. As indicated, CD3, AQP1, and numerous HLA-D antibodies (labelled as HLA-oligo-D) were identified by metal-conjugated secondary antibodies in the first round of staining, and all others as primary antibodies in the second overnight stain. Images were acquired using a Hyperion Imaging System (Fluidigm). Regions of interest for fetal brain tissues were selected based on CD31 IHC stained serial sections. All fetal and adult brain tissue sections were laser-ablated in a rasterized pattern at 400Hz. Raw data was converted to TIFF format (<https://github.com/BodenmillerGroup/imctoolkit>) as previously described and scaled for proper visualization of each image using HistoCAT++ software.

**Spatial Transcriptomics - Visualization of MHC class II gene expression on 10X Genomics Visium data**

To examine the spatial MHC class II gene expression, we processed the publicly available Visium data for human adult/control brains (cerebral cortex) and human glioblastoma (<https://www.10xgenomics.com/resources/datasets/adult-human-brain-1-cerebral-cortex-unknown-orientation-stains-anti-gfap-anti-nfh-1-standard-1-1-0>) and (https://www.10xgenomics.com/resources/datasets/human-glioblastoma-whole-transcriptome-analysis-1-standard-1-2-0) using the Seurat v4 workflow . In brief, data was sc-transformed using the "SCTransform" and spatial expression plots were generated using "SpatialFeaturePlot" functions.

**Validation of AV-specification markers in the Human Protein Atlas**

Expression of top marker genes along AV-zonation was examined by assessment of IHC stainings in the Human Protein Atlas (<https://www.proteinatlas.org/>). Novel human (not previously reported in human) and human-specific AV-markers and well as mouse-human conserved AV-markers were examined ^3,36^. Among the available stainings, those with the clearest vessel structures were chosen. Staining in endothelial cells was confirmed through HPA annotation.

**Statistics and reproducibility**

Immunostaining (immunofluorescence), imaging mass cytometry (IMC) and RNAscope validation experiments were repeated independently at least twice with similar results. As indicated in the figure legends, some immunostaining images come from the Human Protein Atlas and are available at https://www.proteinatlas.org/.

**References**

1 Schramm, J. Temporal lobe epilepsy surgery and the quest for optimal extent of resection: a review. *Epilepsia* **49**, 1296-1307, doi:10.1111/j.1528-1167.2008.01604.x (2008).

2 Winkler, E. A. *et al.* A single-cell atlas of the normal and malformed human brain vasculature. *Science* **375**, eabi7377, doi:10.1126/science.abi7377 (2022).

3 Tang, M. *et al.* Evaluating single-cell cluster stability using the Jaccard similarity index. *Bioinformatics* **37**, 2212-2214, doi:10.1093/bioinformatics/btaa956 (2021).

4 Luecken, M. D. *et al.* Benchmarking atlas-level data integration in single-cell genomics. *Nat Methods* **19**, 41-50, doi:10.1038/s41592-021-01336-8 (2022).

5 Vanlandewijck, M. *et al.* A molecular atlas of cell types and zonation in the brain vasculature. *Nature* **554**, 475-480, doi:10.1038/nature25739 (2018).

6 Goveia, J. *et al.* An Integrated Gene Expression Landscape Profiling Approach to Identify Lung Tumor Endothelial Cell Heterogeneity and Angiogenic Candidates. *Cancer Cell* **37**, 21-36.e13, doi:10.1016/j.ccell.2019.12.001 (2020).

7 Kalucka, J. *et al.* Single-Cell Transcriptome Atlas of Murine Endothelial Cells. *Cell* **180**, 764-779.e720, doi:10.1016/j.cell.2020.01.015 (2020).

8 Han, X. *et al.* Construction of a human cell landscape at single-cell level. *Nature* **581**, 303-309, doi:10.1038/s41586-020-2157-4 (2020).

9 Hodge, R. D. *et al.* Conserved cell types with divergent features in human versus mouse cortex. *Nature* **573**, 61-68, doi:10.1038/s41586-019-1506-7 (2019).

10 Schaum, N. *et al.* Single-cell transcriptomics of 20 mouse organs creates a Tabula Muris. *Nature* **562**, 367-372, doi:10.1038/s41586-018-0590-4 (2018).

11 Buttner, M., Ostner, J., Muller, C. L., Theis, F. J. & Schubert, B. scCODA is a Bayesian model for compositional single-cell data analysis. *Nat Commun* **12**, 6876, doi:10.1038/s41467-021-27150-6 (2021).

12 Ostner, J., Carcy, S. & Muller, C. L. tascCODA: Bayesian Tree-Aggregated Analysis of Compositional Amplicon and Single-Cell Data. *Front Genet* **12**, 766405, doi:10.3389/fgene.2021.766405 (2021).

13 Maier, M. J. DirichletReg: Dirichlet Regression for Compositional Data in R. *Research Report Series* (2014).

14 Phipson, B. *et al.* propeller: testing for differences in cell type proportions in single cell data. *Bioinformatics* **38**, 4720-4726, doi:10.1093/bioinformatics/btac582 (2022).

15 Jonathan Mitchel, M. G. G., Richard K. Perez, Evan Biederstedt, Raymund Bueno, View ORCID ProfileChun Jimmie Ye, View ORCID ProfilePeter V. Kharchenko. Tensor decomposition reveals coordinated multicellular patterns of transcriptional variation that distinguish and stratify disease individuals. *biorxiv* (2022).

16 Chu, T., Wang, Z., Pe'er, D. & Danko, C. G. Cell type and gene expression deconvolution with BayesPrism enables Bayesian integrative analysis across bulk and single-cell RNA sequencing in oncology. *Nat Cancer* **3**, 505-517, doi:10.1038/s43018-022-00356-3 (2022).

17 Racle, J. & Gfeller, D. EPIC: A Tool to Estimate the Proportions of Different Cell Types from Bulk Gene Expression Data. *Methods Mol Biol* **2120**, 233-248, doi:10.1007/978-1-0716-0327-7_17 (2020).

18 Subramanian, A. *et al.* Gene set enrichment analysis: A knowledge-based approach for interpreting genome-wide expression profiles. *Proceedings of the National Academy of Sciences* **102**, 15545, doi:10.1073/pnas.0506580102 (2005).

19 Reimand, J. *et al.* Pathway enrichment analysis and visualization of omics data using g:Profiler, GSEA, Cytoscape and EnrichmentMap. *Nature Protocols* **14**, 482-517, doi:10.1038/s41596-018-0103-9 (2019).

20 Shannon, P. *et al.* Cytoscape: a software environment for integrated models of biomolecular interaction networks. *Genome Res* **13**, 2498-2504, doi:10.1101/gr.1239303 (2003).

21 Munji, R. N. *et al.* Profiling the mouse brain endothelial transcriptome in health and disease models reveals a core blood–brain barrier dysfunction module. *Nature Neuroscience* **22**, 1892-1902, doi:10.1038/s41593-019-0497-x (2019).

22 Yang, A. C. *et al.* A human brain vascular atlas reveals diverse mediators of Alzheimer's risk. *Nature* **603**, 885-892, doi:10.1038/s41586-021-04369-3 (2022).

23 Garcia, F. J. *et al.* Single-cell dissection of the human brain vasculature. *Nature* **603**, 893-899, doi:10.1038/s41586-022-04521-7 (2022).

24 Qiu, X. *et al.* Reversed graph embedding resolves complex single-cell trajectories. *Nat Methods* **14**, 979-982, doi:10.1038/nmeth.4402 (2017).

25 Trapnell, C. *et al.* The dynamics and regulators of cell fate decisions are revealed by pseudotemporal ordering of single cells. *Nature Biotechnology* **32**, 381-386, doi:10.1038/nbt.2859 (2014).

26 Zhicheng Ji, H. J. TSCAN: Tools for Single-Cell Analysis. R package version 1.34.0. (2022).

27 Cao, J. *et al.* The single-cell transcriptional landscape of mammalian organogenesis. *Nature* **566**, 496-502, doi:10.1038/s41586-019-0969-x (2019).

28 La Manno, G. *et al.* RNA velocity of single cells. *Nature* **560**, 494-498, doi:10.1038/s41586-018-0414-6 (2018).

29 Bergen, V., Lange, M., Peidli, S., Wolf, F. A. & Theis, F. J. Generalizing RNA velocity to transient cell states through dynamical modeling. *Nat Biotechnol* **38**, 1408-1414, doi:10.1038/s41587-020-0591-3 (2020).

30 Lange, M. *et al.* CellRank for directed single-cell fate mapping. *Nat Methods* **19**, 159-170, doi:10.1038/s41592-021-01346-6 (2022).

31 Angerer, P. *et al.* destiny: diffusion maps for large-scale single-cell data in R. *Bioinformatics* **32**, 1241-1243, doi:10.1093/bioinformatics/btv715 (2016).

32 Wolf, F. A. *et al.* PAGA: graph abstraction reconciles clustering with trajectory inference through a topology preserving map of single cells. *Genome Biol* **20**, 59, doi:10.1186/s13059-019-1663-x (2019).

33 Efremova, M., Vento-Tormo, M., Teichmann, S. A. & Vento-Tormo, R. CellPhoneDB: inferring cell–cell communication from combined expression of multi-subunit ligand–receptor complexes. *Nature Protocols* **15**, 1484-1506, doi:10.1038/s41596-020-0292-x (2020).

34 Jin, S. *et al.* Inference and analysis of cell-cell communication using CellChat. *Nature Communications* **12**, 1088, doi:10.1038/s41467-021-21246-9 (2021).

35 Abramoff, M., Magalhães, P. & Ram, S. J. Image Processing with ImageJ. *Biophotonics International* **11**, 36-42 (2003).

36 Uhlen, M. *et al.* Proteomics. Tissue-based map of the human proteome. *Science* **347**, 1260419, doi:10.1126/science.1260419 (2015).
