# Supplementary material for: Single-cell atlas of the human brain vasculature across development, adulthood and disease
Source: Nature. 2024 Jul 10;632(8025):603–13. doi: 10.1038/s41586-024-07493-y (PMC11324530; doi:10.1038/s41586-024-07493-y)
Supplement: Supplementary file 5 — Supplementary Discussion. [file 41586_2024_7493_MOESM5_ESM.docx]

**Supplementary Discussion**

Our data suggest a paradigm in which developmentally established characteristics and activated genes and pathways of the fetal brain vasculature are silenced in the adult control brain and (re)activated in the vasculature across various brain pathologies termed the “patho-fetal axis” for pathologies and “onco-fetal axis” for tumors[^1^](#_ENREF_1)^,^[^15^](#_ENREF_15)^,^[^21^](#_ENREF_20)^,^[^106^](#_ENREF_105), indicating functional plasticity of the endothelial lineage across developmental and disease states. Molecular profiling of the human cerebrovasculature reveals human-specific characteristics of ECs and PVCs as well as of EC clusters along the arteriovenous axis, suggesting baseline differences in BBB regulation across species[^34-36^](#_ENREF_33).

We uncover the transcriptional basis of the cellular and molecular heterogeneity and similarity in the fetal, adult/control and pathological human brain vasculature thereby enabling us to identify a treasure trove of novel findings that includes properties conserved across pathologies characterizing common ''hallmarks'' of the diseased human brain vasculature: we observe specific alterations of arteriovenous differentiation, as well as dysregulated/aberrant- and reactivated fetal pathways conserved in the diseased vasculature across multiple pathologies[^1^](#_ENREF_1) (reactivated fetal/developmental signaling pathways in disease are shared pathways between development and disease whereas silenced in the adult[^1^](#_ENREF_1)^,^[^21^](#_ENREF_20); an interesting concept requiring further future validation[^1^](#_ENREF_1)^,^[^21^](#_ENREF_20); for instance, it remains to be clarified whether the pathways observed in brain pathologies are reactivated developmental pathways or rather reflect the persistence (for example, the presence since development) of a less differentiated cell type (or even a combination of these two)[^1^](#_ENREF_1).

Pathological ECs display a loss of CNS-specific properties and reveal an upregulation of MHC class II molecules, indicating atypical features of pathological CNS ECs. CNS-specificity and BBB properties of ECs revealed phenotypic zonation along the arteriovenous axis in the fetus and adult mainly at the level of small-caliber vessels. Zonal characteristics also arise in disease states, where CNS ECs take on a peripheral signature at the level of large>small-caliber vessels and angiogenic capillaries. Altered CNS-specific and BBB properties of brain tumor and vascular malformation ECs showed common and different features when compared to ECs of neurodegenerative brain diseases[^3^](#_ENREF_3)^,16,^[^34-36^](#_ENREF_33).

We further identified ECs expressing MHC class II genes mainly in diseased large-caliber vessels, in close proximity to immune cells, suggesting a role for ECs in immune surveillance[^100^](#_ENREF_99). Our work also revealed that upregulation of MHC class II gene expression partially co-occurs with alteration of CNS-specificity and acquisition of a peripheral signature, suggesting these observations might be linked, as recently suggested for immune activation and loss of BBB tight junction protein expression in the mouse brain[^93^](#_ENREF_92). We have thus unveiled a molecular blueprint for zonation and fundamental EC properties (CNS-specificity, MHC class II expression) along the arteriovenous axis at the single-cell level.

Cell-cell interaction analysis predicted strong endothelial-to-perivascular cell crosstalk involving immune-related (including MHC class II, for which we show spatial co-localization and physical proximity between MHC class II expressing ECs and immune cells) and angiogenic pathways, thereby unraveling a central role for the endothelium within developing, adult/control and diseased brain NVU signaling networks. Our findings suggest a cellular and molecular environment within the NVU with notable parallels between the fetal adult and pathological brains, in which ECs increase their crosstalk with other cell types. The strength of our molecular landscape study lies in its broad coverage of fresh human fetal, adult and diseased brain tissues and the focus on sorted ECs. Despite the unprecedented amount of single cell-sequenced brain vascular ECs and PVCs, we recognize the limitations regarding the sample sizes per disease group and developmental fetal stages as well as unintended bias pertaining to surgical tissue sampling.

Considering the organ-specific properties of the brain vasculature[^1^](#_ENREF_1)^,^[^3^](#_ENREF_3)^,^[^38^](#_ENREF_37), it will be exciting to investigate to which degree the fundamental observations in the developing, healthy, and diseased brain vasculature made here (including reactivated fetal pathways, AV-differentiation and EC heterogeneity, alteration of AV-specification and of CNS/organ-specific properties, upregulation of MHC class II receptors, and the central role of ECs in the brain NVU) are broadly applicable beyond the cerebrovasculature. Considering the interplay of organ-specific and general mechanisms guiding vascular growth and differentiation[^1^](#_ENREF_1)^,^[^3^](#_ENREF_3)^,^[^38^](#_ENREF_37)^,^[^107^](#_ENREF_107), we anticipate a mixture between organ-specific and generalized properties of developing, healthy, and diseased blood vessels (and their endothelial- and perivascular cells) across tissues[^108^](#_ENREF_108).

We anticipate that our work will inspire studies with large cohorts to address the specific roles of age, sex and brain-region specificity on the fetal, normal and diseased brain (and peripheral) vasculature, trigger further studies[^1^](#_ENREF_1) to examine the genomic, epigenomic and proteomic single-cell heterogeneity and to resolve the development of the fetal human brain vasculature over time, and propel investigations of molecular subgroups in the different diseases (e.g. IDH1 and MGMT status[^109^](#_ENREF_109)^,^[^110^](#_ENREF_110) in glial brain tumors, different neoplasms in lung cancer metastasis[^111^](#_ENREF_111), KRAS mutation status in AVMs[^18^](#_ENREF_17), and others).

106 Wälchli, T., Farnhammer, F. & Fish, J. E. MicroRNA-Based Regulation of Embryonic Endothelial Cell Heterogeneity at Single-Cell Resolution. *Arterioscler Thromb Vasc Biol* **42**, 343-347, doi:10.1161/ATVBAHA.122.317400 (2022).

107 Nolan, D. J. *et al.* Molecular signatures of tissue-specific microvascular endothelial cell heterogeneity in organ maintenance and regeneration. *Dev Cell* **26**, 204-219, doi:10.1016/j.devcel.2013.06.017 (2013).

108 Trimm, E. & Red-Horse, K. Vascular endothelial cell development and diversity. *Nat Rev Cardiol* **20**, 197-210, doi:10.1038/s41569-022-00770-1 (2023).

109 Neftel, C. *et al.* An Integrative Model of Cellular States, Plasticity, and Genetics for Glioblastoma. *Cell* **178**, 835-849 e821, doi:10.1016/j.cell.2019.06.024 (2019).

110 Richards, L. M. *et al.* Gradient of Developmental and Injury Response transcriptional states defines functional vulnerabilities underpinning glioblastoma heterogeneity. *Nature Cancer* **2**, 157-173, doi:10.1038/s43018-020-00154-9 (2021).

111 Gonzalez, H. *et al.* Cellular architecture of human brain metastases. *Cell* **185**, 729-745 e720, doi:10.1016/j.cell.2021.12.043 (2022).
