# Supplementary material for: Single-cell atlas of the human brain vasculature across development, adulthood and disease
Source: Nature. 2024 Jul 10;632(8025):603–13. doi: 10.1038/s41586-024-07493-y (PMC11324530; doi:10.1038/s41586-024-07493-y)
Supplement: Supplementary file 6 — Supplementary Tables 1–27 and the legends for the Supplementary Tables. [file 41586_2024_7493_MOESM6_ESM.zip › Supplementary Table Guide.docx]

| Supplementary Table 1 | Overview of pathological, age/sex and clinical description of the samples/patients utilized in sc-RNA-seq experiments of FACS sorted CD31^+^/CD45^-^ endothelial cells and unsorted endothelial and perivascular cells |
| --- | --- |
| Supplementary Table 2 | Overview of pathological, age/sex and clinical description of the samples/patients utilized in bulk RNA-seq experiments of FACS sorted CD31^+^/CD45^-^ endothelial cells |
| Supplementary Table 3 | Overview of the number of samples/patients and of the final number of single cells analyzed via scRNA-seq for every fetal, adult and disease entity |
| Supplementary Table 4 | Overview of the number of single-cells analyzed via scRNA-seq before and after quality control (QC) filtering per sample/patient. |
| Supplementary Table 5 | Quality control (QC) metrics per sample/patient:  - mean number of transcripts  - mean number of genes  - mean percentage of mitochondrial genes  - ambient RNA quantification per entity |
| Supplementary Table 6 | Differential expression analysis comparing: - fetal brain endothelial cells (CNS)  vs  - fetal peripheral endothelial cells (non-CNS)  Differential expression was computed using the wilcoxauc function (Wilcoxon rank sum test) implemented in the github package presto (https://github.com/immunogenomics/presto). FDR values were calculated using the Benjamini–Hochberg method. |
| Supplementary Table 7 | Differential expression analysis comparing:  - adult/control brain (=temporal lobe) endothelial cells (AdultTL)  vs  - fetal brain endothelial cells (Fetal CNS EC)  Differential expression was computed using the wilcoxauc function (Wilcoxon rank sum test) implemented in the github package presto (https://github.com/immunogenomics/presto). FDR values were calculated using the Benjamini–Hochberg method. |
| Supplementary Table 8 | Differential expression analysis comparing: - adult/control brain (=temporal lobe) endothelial cells (AdultTL)  vs  - fetal brain endothelial cells (FCNS) vs - fetal periphery endothelial cells (FPERIPH) vs - brain tumor endothelial cells (TUM) vs - brain vascular malformations endothelial cells (MAL)  Differential expression was computed using the wilcoxauc function (Wilcoxon rank sum test) implemented in the github package presto (https://github.com/immunogenomics/presto). FDR values were calculated using the Benjamini–Hochberg method. |
| Supplementary Table 9 | Differential expression analysis comparing: - adult/control brain (=temporal lobe) endothelial cells (TL)  vs  - fetal brain endothelial cells (FETALCNS) vs - fetal periphery endothelial cells (FETALnon-CNS) vs - brain arteriovenous malformations endothelial cells (AVM) vs - glioblastoma endothelial cells (GBM) vs - Lower-grade glioma endothelial cells (LGG) vs - brain metastasis endothelial cells (MET) vs - brain meningioma endothelial cells (MEN)  Differential expression was computed using the wilcoxauc function (Wilcoxon rank sum test) implemented in the github package presto (https://github.com/immunogenomics/presto). FDR values were calculated using the Benjamini–Hochberg method. |

| Supplementary Table 10 | Proportions of the different endothelial subtypes (AV clusters) in the indicated entities analyzed via single cell RNA-seq of CD31+/CD45- FACS sorted endothelial cells:  - All endothelial cells (the overall merge of all brain endothelial cells including fetal brain, adult/control brain, brain tumors (glioblastoma, lower-grade glioma, metastasis and meningioma) and brain vascular malformations (AVM) endothelial cells  - All pathological ECs (the overall merge of all pathological brain endothelial cells including brain tumors (glioblastoma, lower-grade glioma, metastasis and meningioma) and brain vascular malformations (AVM) endothelial cells  -All malformation ECs (the brain arteriovenous malformations (AVM) endothelial cells)  - All tumor ECs (the overall merge of all brain tumors (glioblastoma, lower-grade glioma, metastasis and meningioma) endothelial cells  - Fetal brain ECs (the fetal brain (CNS) endothelial cells)  - adult/control brain ECs (temporal lobe (adult/control brain endothelial cells)  - AVM ECs (the brain arteriovenous malformations endothelial cells)  - LGG ECs (Lower-grade glioma endothelial cells)  - GBM ECs (glioblastoma (high-grade glioma) endothelial cells)  - Metastasis ECs (lung brain metastasis endothelial cells)  - Meningioma ECs (meningioma endothelial cells) |
| --- | --- |
| Supplementary Table 11 | Proportions of the different cell types in the indicated entities analyzed via single cell RNA-seq of unsorted endothelial and perivascular cells:  - All brian cells_unsorted (the overall merge of all brain cells including fetal brain, adult/control brain, brain tumors (glioblastoma, lower-grade glioma, metastasis and meningioma) and brain vascular malformations (AVM) cells  - All pathological brain cells (the overall merge of all pathological brain cells including brain tumors (glioblastoma, lower-grade glioma, metastasis and meningioma) and brain vascular malformations (AVM) cells  -All brain vascular malformation (the brain vascular malformations (AVM) cells)  - All brain tumor cells (the overall merge of all brain tumors (glioblastoma, lower-grade glioma, metastasis and meningioma) cells)  - Fetal brain (the fetal brain (CNS) cells)  - Adult control brain (TL) (temporal lobe (adult/control brain cells)  - Arteriovenous malformations (the brain vascular malformations cells)  - Lower grade glioma (Lower-grade glioma cells)  - Glioblastoma (glioblastoma (high-grade glioma) cells)  - Metastasis (lung brain metastasis cells)  - Meningioma (meningioma cells) |

| Supplementary Table 12 | scCODA compositional analysis comparison for the indicated entities  *sorted EC-TLvsFETAvsPATH* comparison of:  - adult/control brain vs fetal brain endothelial cells - adult/control brain vs pathological brain endothelial cells  *sorted EC-TLvsFETALvsMALvsTUM* comparison of:  - adult/control brain vs fetal brain endothelial cells - adult/control brain vs brain vascular malformations endothelial cells - adult/control brain vs brain tumors endothelial cells  *sorted EC-individual entity TL* comparison of:  - adult/control brain vs brain vascular malformations endothelial cells - adult/control brain vs fetal brain endothelial cells - adult/control brain vs glioblastoma endothelial cells - adult/control brain vs lower-grade glioma endothelial cells - adult/control brain vs meningioma endothelial cells - adult/control brain vs metastasis endothelial cells  *unsorted-TLvsFETAvsPATH*  comparison of:  - adult/control brain vs fetal brain cells - adult/control brain vs pathological brain cells  *unsorted-TLvsFETALvsMALvsTUM*  comparison of:  - adult/control brain vs fetal brain cells - adult/control brain vs brain vascular malformations cells - adult/control brain vs brain tumors cells  *unsorted-individual entityTL*  comparison of:  - adult/control brain vs brain vascular malformations cells - adult/control brain vs fetal brain cells - adult/control brain vs glioblastoma cells - adult/control brain vs lower-grade glioma cells - adult/control brain vs meningioma cells - adult/control brain vs metastasis cells  *TL ECs Males vs females*  comparison of:  - male vs female adult/control brain (temporal lobe) endothelial cells  *AVM ECs Males vs females*  comparison of:  - male vs female brain arteriovenous malformations endothelial cells  *GBM ECs Males vs females*  comparison of:  - male vs female glioblastoma endothelial cells  *MET ECs Males vs females*  comparison of:  - male vs female brain metastasis endothelial cells  *MEN ECs Males vs females*  comparison of:  - male vs female brain meningioma endothelial cells  *LGG ECs Males vs females*  comparison of:  - male vs female lower-grade glioma endothelial cells  *Lung MET LAC vs SCLC* comparison of:  - lung adenocarcinoma brain metastasis (LAC) vs small cell lung cancer brain metastasis (SCLC) endothelial cells  *GBM-MGMT methylated vs non* comparison of: - glioblastoma endothelial cells in MGMT methylated vs MGMT non-methylated patient samples  *TL ECs 15yrs old vs older wo 69*  comparison of: - 15 years old vs all other older adult/control brain (temporal lobe) endothelial cells  *TL ECs 69yrs old vs young wo 15*  comparison of: - 69 years old vs all other younger temporal lobe samples (without the 15 year old sample) adult/control brain (temporal lobe) endothelial cells  *FETAL CNS ages comparison*  comparison of: - fetal brain (CNS) samples’ endothelial cells |
| --- | --- |
| Supplementary Table 13 | tascCODA compositional analysis comparison for the indicated entities  *no regression*  comparison (without regression) of:  - adult/control brain vs brain vascular malformations endothelial cells - adult/control brain vs fetal brain endothelial cells - adult/control brain vs glioblastoma endothelial cells - adult/control brain vs lower-grade glioma endothelial cells - adult/control brain vs meningioma endothelial cells - adult/control brain vs metastasis endothelial cells  *age+sex regression*  comparison (with age+sex regression) of:  - adult/control brain vs brain vascular malformations endothelial cells - adult/control brain vs fetal brain endothelial cells - adult/control brain vs glioblastoma endothelial cells - adult/control brain vs lower-grade glioma endothelial cells - adult/control brain vs meningioma endothelial cells - adult/control brain vs metastasis endothelial cells  *age regression*  comparison (with age regression) of:  - adult/control brain vs brain vascular malformations endothelial cells - adult/control brain vs fetal brain endothelial cells - adult/control brain vs glioblastoma endothelial cells - adult/control brain vs lower-grade glioma endothelial cells - adult/control brain vs meningioma endothelial cells - adult/control brain vs metastasis endothelial cells  *sex regression*  comparison (with sex regression) of:  - adult/control brain vs brain vascular malformations endothelial cells - adult/control brain vs fetal brain endothelial cells - adult/control brain vs glioblastoma endothelial cells - adult/control brain vs lower-grade glioma endothelial cells - adult/control brain vs meningioma endothelial cells - adult/control brain vs metastasis endothelial cells |
| Supplementary Table 14 | DirichReg compositional analysis comparison for the indicated entities (with regression of age and sex)  *sorted ECs-TL vs all PATH* comparison of:  - adult/control brain vs pathological brain endothelial cells  *sorted ECs-TL vs all MAL*  comparison of:  - adult/control brain vs brain vascular malformations endothelial cells  *sorted ECs-TL vs all TUM*  comparison of:  - adult/control brain vs brain tumor endothelial cells  *sorted ECs-TL vs Fetal*  comparison of:  - adult/control brain vs fetal brain endothelial cells  *sorted ECs-TL vs AVM*:  comparison of: - adult/control brain vs brain vascular malformations endothelial cells  *sorted ECs-TL vs LGG*  comparison of:  - adult/control brain vs lower-grade glioma endothelial cells  *sorted ECs-TL vs GBM* comparison of: - adult/control brain vs glioblastoma endothelial cells  *sorted ECs-TL vs MET*  comparison of:  - adult/control brain vs metastasis endothelial cells  *sorted ECs-TL vs MEN* comparison of:  - adult/control brain vs meningioma endothelial cells |
| Supplementary Table 15 | Propeller (<https://github.com/phipsonlab/speckle>, https://doi.org/10.1093/bioinformatics/btac582) compositional analysis comparison for the indicated entities (with: no regression, regression of age and sex, regression of age only, regression of sex only)  *sorted ECs-TL vs all PATH* comparison of:  - adult/control brain vs pathological brain endothelial cells  *sorted ECs-TL vs all MAL*  comparison of:  - adult/control brain vs brain vascular malformations endothelial cells  *sorted ECs-TL vs all TUM*  comparison of:  - adult/control brain vs brain tumor endothelial cells  *sorted ECs-TL vs Fetal*  comparison of:  - adult/control brain vs fetal brain endothelial cells  *sorted ECs-TL vs AVM*:  comparison of: - adult/control brain vs brain vascular malformations endothelial cells  *sorted ECs-TL vs LGG*  comparison of:  - adult/control brain vs lower-grade glioma endothelial cells  *sorted ECs-TL vs GBM* comparison of: - adult/control brain vs glioblastoma endothelial cells  *sorted ECs-TL vs MET*  comparison of:  - adult/control brain vs metastasis endothelial cells  *sorted ECs-TL vs MEN* comparison of:  - adult/control brain vs meningioma endothelial cells  *sorted ECs Males vs Females*  *sorted ECs-TL-Males vs Females*  comparison of:  - male vs female adult/control brain (temporal lobe) endothelial cells  *sorted ECs-AVM-Males vs Females*  comparison of:  - male vs female brain arteriovenous malformations endothelial cells  *sorted ECs-LGG-Males vs Females*  comparison of:  - male vs female lower-grade glioma endothelial cells  *sorted ECs-GBM-Males vs Females*  comparison of:  - male vs female glioblastoma endothelial cells  *sorted ECs-MET-Males vs Females*  comparison of:  - male vs female brain metastasis endothelial cells  *sorted ECs-MEN-Males vs Females*  comparison of:  - male vs female brain meningioma endothelial cells  *sorted ECs-GBM-MGMT- methyvsnon* comparison of: - glioblastoma endothelial cells in MGMT methylated vs MGMT non-methylated patient samples  *sorted ECs-TL-old vs young* comparison of:  - old vs young adult/control brain (temporal lobe) endothelial cells  *sorted ECs-ME-LAC vs other type* comparison of:  - lung adenocarcinoma brain metastasis (LAC) vs other types of lung cancer brain metastasis (SCLC) endothelial cells  Propeller (<https://github.com/phipsonlab/speckle>, https://doi.org/10.1093/bioinformatics/btac582) uses moderated t-tests for statistical analysis for two group comparisons. |
| Supplementary Table 16 | Cacoa compositional analysis comparison for the indicated entities  *sorted ECs-TL-males vs females*  comparison of:  - male vs female adult/control brain (temporal lobe) endothelial cells  *sorted ECs-AVM-males vs females*  comparison of:  - male vs female brain arteriovenous malformations endothelial cells  *sorted ECs-LGG-males vs females*  comparison of:  - male vs female lower-grade glioma endothelial cells  *sorted ECs-GBM-males vs females*  comparison of:  - male vs female glioblastoma endothelial cells  *sorted ECs-MET-males vs females*  comparison of:  - male vs female brain metastasis endothelial cells  *sorted ECs-MEN-males vs females*  comparison of:  - male vs female brain meningioma endothelial cells  *sorted ECs-GBM-MGMT* comparison of: - glioblastoma endothelial cells in MGMT methylated vs MGMT non-methylated patient samples |
| Supplementary Table 17 | Comparison of arteriovenous specification markers between our human (our dataset vs mouse datasets (Kalucka et al Cell 2020 and Vanlandewijck et al Nature 2018) |
| Supplementary Table 18 | Top cluster markers oft he 44 endothelial seurat (sub-)clusters of the overall merge of CD31+/CD45- FACS sorted brain endothelial cells  Differential expression was computed using the wilcoxauc function (Wilcoxon rank sum test) implemented in the github package presto (https://github.com/immunogenomics/presto). FDR values were calculated using the Benjamini–Hochberg method. |
| Supplementary Table 19 | Top cluster markers of the 14 endothelial clusters of the overall merge of CD31+/CD45- FACS sorted brain endothelial cells |
| Supplementary Table 20 | Listing of the: - Fetal brain (CNS) endothelial cells’ signature genes  - Fetal periphery endothelial cells’ signature genes - Adult brain (CNS) endothelial cells’ signature genes - Adult periphery endothelial cells’ signature genes  Differential expression was computed using the wilcoxauc function (Wilcoxon rank sum test) implemented in the github package presto (https://github.com/immunogenomics/presto). FDR values were calculated using the Benjamini–Hochberg method.  - Mouse brain endothelial cells’ signature genes (derived from Munji et al., Nature Neuroscience 2019)  - Mouse peripheral endothelial cells’ signature genes (derived from Munji et al., Nature Neuroscience 2019) - BBB signature defined by Zhao et al., Cell 2015 |
| Supplementary Table 21 | Pathological signatures comparisons as indicated in Supplementary Figure 14 o-s |
| Supplementary Table 22 | MHC class II signature genes |
| Supplementary Table 23 | Antibodies used for immunofluorescence (IF) imaging |
| Supplementary Table 24 | Antibodies and their metal tags used for imaging mass cytometry (IMC) |
| Supplementary Table 25 | Differential expression analysis comparing:  *PATH vs TL* - pathological brain (PATH) vs adult/control brain (TL) endothelial cells  *TUM vs TL* - brain tumor (TUM) vs adult/control brain (TL) endothelial cells  *MAL vs TL* - brain vascular malformations (MAL) vs adult/control brain (TL) endothelial cells |
| Supplementary Table 26 | Bulk RNA-seq deconvolution of FACS sorted CD31+/CD45- endothelial cells using EPIC and BayesPrism packages |
| Supplementary Table 27 | Silhouette score - quality control metrics for the measure of the quality of the integration/batch correction |
